# A diazotrophy-ammoniotrophy dual growth model for the sulfate reducing bacterium *Desulfovibrio vulgaris* var. Hildenborough

**DOI:** 10.1016/j.csbj.2023.05.007

**Published:** 2023-05-07

**Authors:** Romain Darnajoux, Keisuke Inomura, Xinning Zhang

**Affiliations:** a Department of Geosciences, Princeton University, Princeton, NJ 08544, USA; b High Meadow Environmental Institute, Princeton University, Princeton, NJ 08544, USA; c Graduate School of Oceanography, University of Rhode Island, Narragansett, RI 02882, USA

**Keywords:** *Desulfovibrio vulgaris* var. Hildenborough, Biological nitrogen fixation, Anaerobic heterotroph, Benthic sediments biogeochemistry, Quantitative model

## Abstract

Sulfate reducing bacteria (SRB) comprise one of the few prokaryotic groups in which biological nitrogen fixation (BNF) is common. Recent studies have highlighted SRB roles in N cycling, particularly in oligotrophic coastal and benthic environments where they could contribute significantly to N input. Most studies of SRB have focused on sulfur cycling and SRB growth models have primarily aimed at understanding the effects of electron sources, with N usually provided as fixed-N (nitrate, ammonium). Mechanistic links between SRB nitrogen-fixing metabolism and growth are not well understood, particularly in environments where fixed-N fluctuates. Here, we investigate diazotrophic growth of the model sulfate reducer *Desulfovibrio vulgaris* var. Hildenborough under anaerobic heterotrophic conditions and contrasting N availabilities using a simple cellular model with dual ammoniotrophic and diazotrophic modes. The model was calibrated using batch culture experiments with varying initial ammonium concentrations (0–3000 µM) and acetylene reduction assays of BNF activity. The model confirmed the preferential usage of ammonium over BNF for growth and successfully reproduces experimental data, with notably clear bi-phasic growth curves showing an initial ammoniotrophic phase followed by onset of BNF. Our model enables quantification of the energetic cost of each N acquisition strategy and indicates the existence of a BNF-specific limiting phenomenon, not directly linked to micronutrient (Mo, Fe, Ni) concentration, by-products (hydrogen, hydrogen sulfide), or fundamental model metabolic parameters (death rate, electron acceptor stoichiometry). By providing quantitative predictions of environment and metabolism, this study contributes to a better understanding of anaerobic heterotrophic diazotrophs in environments with fluctuating N conditions.

## Highlights

• We create a Diazotrophic-Ammoniotrophic Dual Growth Model (DAD-GM) for anaerobic heterotrophs.
• We reproduce the observed growth of sulfate reducers with fluctuations in [NH_4_^+^].
• Cost of N fixation is likely between 12 and 22 ATP per mol of N fixed.
• Cost of NH_4_^+^-uptake was found to be 2–3 ATP per mol N.
• The model suggests a nitrogen fixation specific limitation phenomenon in pure culture.

## Graphical Abstract

**Figure:**
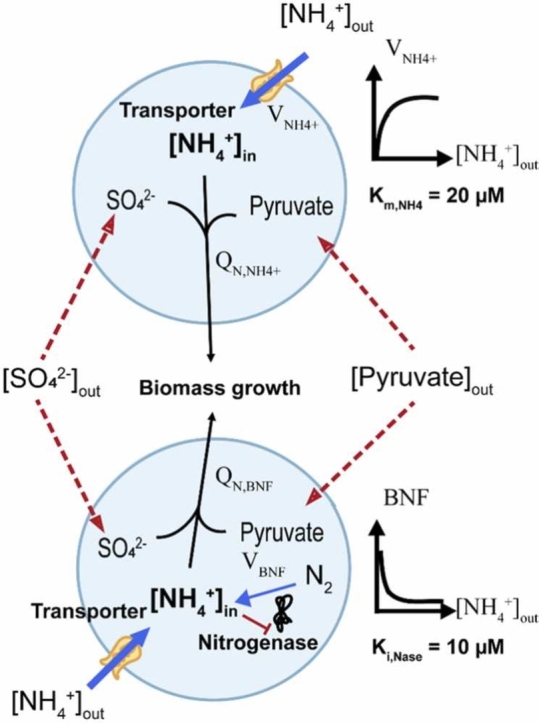

## 1 Introduction

Nitrogen (N) is the fourth most abundant element in biological systems, and its availability often constrains primary production in terrestrial [17], [37], [57] and oceanic ecosystems [18], [55], [60]. A phylogenetically diverse group of prokaryotes have evolved the specialized ability to acquire new N from the large atmospheric N_2_ reservoir using a process termed biological nitrogen fixation (BNF) catalyzed by the enzyme nitrogenase. The ecological advantage provided by BNF has allowed N fixers to successfully colonize a diversity of environments characterized by low N availability where they help support primary production. Over the last two centuries, the development of human societies has substantially impacted N cycling, with reactive forms of N entering natural environments at higher rates, widely alleviating N limitation but often favoring invasive species [48], and contributing to long term greenhouse gas production [38], [7]. A better understanding of how increases in N availability will influence the activity and adaptability of N-fixing organisms, particularly in carbon (C)-rich ecosystems like fresh water and marine sediments, is important to understand their resilience and contribution to ecosystem services in the long term under anthropic pressure.

Sulfate reducing bacteria (SRB), most of which are deltaproteobacteria, have well-accepted roles in benthic biogeochemistry [21], [3], [4], [41], [44] based on their use of sulfate as the terminal electron acceptor for oxidative transformations of organic carbon and hydrogen (H_2_). Widespread findings of diverse SRB-like *nif* genes, which encode for the most ubiquitous form of nitrogenase, within sediments with relatively high fixed-N availability have been surprising, prompting questions on the role and regulating controls on benthic BNF [36], [40], [41].

One fundamental reason why fixed-N is generally thought to be preferred over BNF lies in the higher metabolic cost of BNF [15], [22]. For instance, NH_4_^+^ transport by the most abundant *amt*B transporter could cost only 1 ATP, the cost of one ion transport, or perhaps even no energy expenditure if transport occurs in the deprotonated form, NH_3_ [31], [32], [61]. In contrast, the chemical bond between the two N atoms in N_2_ is among the strongest, and 16 moles of ATP are required for the biochemical conversion of one mole of N_2_ into 2 moles of NH_4_^+^_,_ with the obligate production of 1 mol of H_2_ as a by-product [50], [51]. Based on the direct costs of N acquisition, ammonium is thus possibly ∼ 8 times less costly a source of N than BNF. Additionally, indirect metabolic costs such as biosynthesis and enzyme maintenance in N and related pathways can substantially impact the overall cost of N acquisition for an organism [23]. Further, diazotrophs also commonly possess transporters to access “cheaper” forms of nitrogen such as ammonium (NH_4_^+^) [22], [35].

Yet, several studies in benthic environments have reported the presence of *nif* genes, coding for the canonical Mo-nitrogenase, in areas with relatively high bulk levels of ammonium, which have raised questions on the possible contributions of BNF to N input in the presence of NH_4_^+^ and on the spatio-temporal complexity of benthic N cycling [36]. In support of the traditional conceptualization of BNF as a less-preferred N source, recent findings showed clear downregulation of benthic BNF activity in the presence of ammonium [11], [13]. However, measured NH_4_^+^ thresholds for BNF inhibition in naturally heterogeneous benthic sediments were relatively high (<30 µM NH_4_^+^, [13], 0–20 µM NH_4_^+^, [11]) as compared to cellular thresholds in liquid culture (<2 µM, [11]). This difference suggests that sediment bio-physical complexity (e.g., nutrient diffusion, remineralization, flow heterogeneity) could significantly obscure our ability to observe “true” sensitivities in various sediment types. Similarly, a mechanistic understanding of how in-situ heterogeneity influences biological activities has been limited by our capacities for measuring key phenomena at microbiologically-relevant time and spatial scales. By disentangling biology from physicochemical factors, quantitative growth models could help provide a refined mechanism of physical, chemical and metabolic interactions in real ecosystems [14], [30].

In order to better quantify and predict metabolic differences in nitrogen acquisition by anaerobic benthic diazotrophs, we developed a simple cellular growth model for the anaerobic heterotroph *Desulfovibrio vulgaris* var. Hildenborough (DvH) [25], a well-known sulfate reducer, which resolves both NH_4_^+^ uptake and BNF as N sources. This is the first dual N source model for anaerobic heterotrophic N fixers. We parameterized the model using measurement data from the growth of DvH in batch cultures with increasing initial [NH_4_^+^] ranging from the background (∼10 µM) to 3000 µM and literature values of ammonium transporter enzyme kinetics [10], [35]. Finally, we discuss the implications of our findings for understanding N flows in benthic environments and the potential applications and limitations of the proposed model. By providing quantitative mechanistic predictions of environment and metabolism, this study contributes to a better understanding of anaerobic diazotrophs in spatiotemporally changing sediment and soil environments.

## 2 Material and methods

### 2.1 Diazotrophic – ammoniotrophic dual growth model (DAD-GM)

**Fig. 1:**
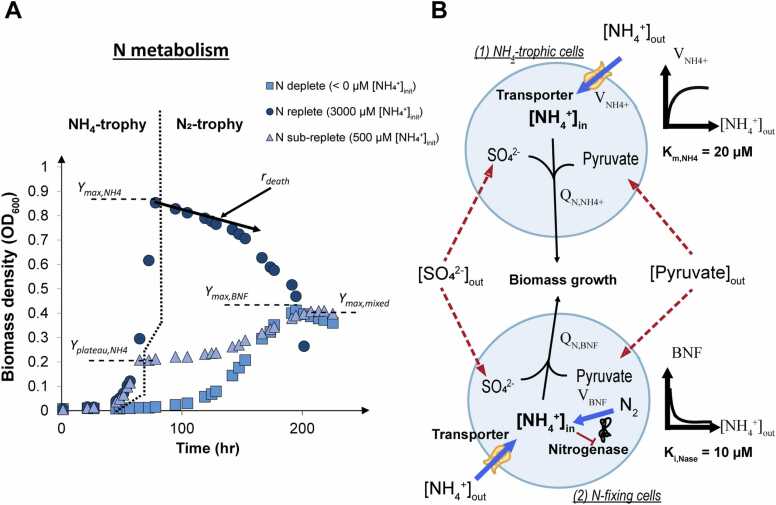
**Summary growth dynamics of*****Dv*****H (modified from**[11]**) and conceptual view of the Diazotrophic-Ammoniotrophic Dual growth model (DAD-GM)**. Panel (A) illustrates the classical characteristics of DvH growth in batch culture under sub-replete initial [NH_4_^+^]. Y_max,i_ refers to maximal biomass yield expressed for a culture of volume of 10 mL as biomass density at 600 nm (OD_600_) under various initial conditions. Panel (B) shows the schematic of the DAD-GM with the main fluxes between the two types of cell considered in the model (ammoniotrophic and diazotrophic). Note that results for cell number are expressed as cell density (OD_600_), to facilitate comparison with laboratory experiments, where volume could change with media sampling.

The DAD-GM is an idealized model that allows a population of bacterial cells to grow on both ammonium and atmospheric dinitrogen as N sources (Fig. 1). In the model, the growth of new biomass (Cell), expressed as ODmL, (i.e., the amount of biomass in 1 milliliter of culture at a density of 1 unit of OD_600_, optical density at 600 nm), is determined by the acquisition of nitrogen (N) by ammonium transporters and nitrogenase enzyme activities following (1), (2). Ammonium transport specific activity ($v_{\mathrm{NH}4}(t)$, expressed in µmol_N_.hr^-1^.ODmL^-1^) follows a typical Michaelis-Menten equation (Eq. 1), with K_M_,_NH4_ (µM) the half-saturation constant of the transporter, [NH_4_^+^](t) the ammonium concentration (in µM) found in the medium at a specific time t (in hr), and $v_{\mathrm{NH}4,\mathrm{MAX}}$, the maximal specific activity of the transporter in µmol_N_.hr^-1^.ODmL^-1^. Similarly, nitrogen fixation specific activity ($v_{\mathrm{BNF}}(t)$, expressed in µmol_N_.hr^-1^.ODmL^-1^) follows a simplified Michaelis-Menten equation (assuming maximal activity) with ammonium as a non-competitive inhibitor of constant K_i,NH4_ (µM), and $v_{\mathrm{BNF},\mathrm{MAX}}$ the maximal specific activity of nitrogenase expressed in µmol_N_.hr^-1^.ODmL^-1^ (Eq. 2). In both cases, enzyme maximal specific activity $v_{\mathrm{NH}4,\mathrm{MAX}}$ and $v_{\mathrm{BNF},\mathrm{MAX}}$ were calibrated experimentally (see Table 2 and below).

(1) $$v_{\mathrm{NH}4+}(t)=v_{\mathrm{NH}4+,\mathrm{MAX}}\times \frac{\left[{\mathrm{NH}}_{4}^{+}\right](t)}{\left[{\mathrm{NH}}_{4}^{+}\right](t)+K_{m,\mathrm{NH}4+}}$$

(2) $$v_{\mathrm{BNF}}(t)=v_{\mathrm{BNF},\mathrm{MAX}}\times \frac{1}{1+\frac{\left[{\mathrm{NH}}_{4}^{+}\right](t)}{K_{i,\mathrm{NH}4+}}}$$

**Table 1:** Mass balance equations used to model the growth of *Dv*H on ammonium (NH_4_^+^) and by biological nitrogen fixation (BNF) following a time (t) increment of dt (i.e., from t to t + dt). Index i refers to either BNF or NH_4_^+^.

Variables	Description (units)	Equations^a^
N_BNF_	Nitrogen (BNF) (µmol)	$dN_{\mathrm{BNF}}(t)={\mathrm{Cell}}_{\mathrm{BNF}}\left(t\right)\times V\times v_{\mathrm{BNF}}(t)\times \mathrm{dt}\times (1-\mathrm{Sat})$
N_NH4_	Nitrogen (NH_4_^+^) (µmol)	$dN_{\mathrm{NH}4}(t)=({\mathrm{Cell}}_{\mathrm{BNF}}\left(t\right)+{\mathrm{Cell}}_{\mathrm{NH}4}\left(t\right)\times V\times v_{\mathrm{NH}4+}(t)\times \mathrm{dt}$
E	Ethylene (µmol)	$\mathrm{dE}(t)={\mathrm{Cell}}_{\mathrm{BNF}}\left(t\right)\times V\times v_{\mathrm{BNF}}(t)\times \mathrm{dt}\times \mathrm{Sat}$
b	Decaying biomass (ODmL)	$b_{i}(t)=r_{\mathrm{death}}\times {\mathrm{Cell}}_{i}(t)\times \mathrm{dt}$
Cell	Cellular biomass (ODmL)	$d{\mathrm{Cell}}_{i}(t)=\frac{dN_{i}(t)}{Q_{N,i}}-b_{i}$(t)
SO4	Sulfate (µmol)	$\mathrm{dSO}4(t)=dN_{i}(t)\times {\left(\frac{\mathrm{dSO}4(t)}{\mathrm{dN}(t)}\right)}_{i}$
Pyr	Pyruvate (µmol)	$\mathrm{dPyr}(t)=\mathrm{dSO}4(t)\times \left(\frac{\mathrm{dPyr}}{\mathrm{dSO}4}\right)$

a The nitrogenase acetylene saturation (Sat, in %), the decay rate (r_death_, in hrs^-1^), and pathway specific nitrogen cellular quota requirement (Q_N,i,_ in mol_N_.ODmL^-1^) are defined in the text (see also Fig. 1 A and 2 B, and Table 2). Enzyme specific activities (v_BNF_ and v_NH4_) are defined in Eqs. 1 and 2. V is the media volume, expressed in mL. Cellular biomass was expressed as OD x mL, or the number of cells found in 1 mL of medium at OD_600_ = 1.

**Table 2:** Growth and metabolic parameters used in DAD-GM.

Model term	Unit	Value used	SD [range]	Description	Source
**r**_**death**_	**hr**^**-1**^	**0.0025**	**0.0001**	Direct estimate from biomass decay after growth completion (Fig. 2B)	This study
**Pyr_SO4_ratio**	**mol**_**Pyr**_**.mol**_**SO4**_^**-1**^	**4**		Empirical value for the incomplete oxidation of pyruvate to acetate	Sim et al. [52]
*Pyr_SO4_ratio*	*mol*_*Pyr*_*.mol*_*SO4*_^*-1*^	*4*	*0.5*	*Calculated from measured values*	*This study*
Pyr_ATP	mol_Pyr_.mol_ATP_^-1^	4		Number of ATP produced per pyruvate use	Carepo et al. [5]
**µ**_**NH4**_	**hr**^**-1**^	**0.105**	**0.005**	Measured growth rate under NH_4_^+^ replete condition ([NH_4_^+^]_init_ =3000 µM)	This study
biomass.mol_N_^-1^	ODml.µmol_N_^-1^	0.353	0.005	Measured as OD yield per [NH_4_^+^]_init_ in a 10 mL culture (Fig. 2D)	This study
**Q**_**N,NH4**_	**µmol**_**N**_**.ODmL**^**-1**^	**2.83**	**0.04**	1/ biomass.mol_N_^-1^	This study
*v*_*max ,NH4*_	µ*mol*_*N*_*.hr*^*-1*^*.ODml*^*-1*^	*0.297*	*0.0148*	Calculated according to Eq. 3	This study
**K**_**M,NH4**_	**µmol**_**N**_**.L**^**-1**^	**20**	**-**	Affinity constant of ammonium transporter, literature value < 20 µmol_N_.L^-1^	Kleiner [35]
Y_NH4_	ODmL	8.2	0.3	Maximal biomass yield (from OD_600_) under replete NH_4_^+^ condition	This study
Pyr_NH4_ratio	mol_Pyr_.mol_NH4_^-1^	13.0	1.4	30 mM of pyruvate uses 2.3 mM of NH_4_^+^	This study
SO4_NH4_ratio	mol_SO4_.mol_NH4_^-1^	3.3	0.3	Calculated as Pyr_NH4_ratio/ Pyr_SO4_ratio	This study
**SO4_NH4_ratio**	**mol**_**SO4**_**.mol**_**NH4**_^**-1**^	**3.2**	**0.3**	Direct estimate from H_2_S measurement during NH_4_^+^-trophy	This study
SO4_OD_NH4	µmol_SO4_.ODmL^-1^	9.7	0.3	H_2_S produced (SO_4_^2-^ reduced) per ODmL during NH_4_^+^-trophy	This study
**v**_**max,BNF**_	**µmol_N_.hr^-1^.ODml^-1^**	**0.042**	**0.018**	N fixation rate for N_2_-trophic culture normalized per biomass and time	This study
**K**_**I,NH4**_	**µmol**_**N**_**.L**^**-1**^	**10**	**[5–17]**	Inhibition constant for BNF, estimated as [NH_4_^+^]_threshold_	Darnajoux et al. [11]
**K**_**M,Ac**_	**%v/v**	**4**	**-**	Michaelis-Menten constant for acetylene for Mo nitrogenase	[12])
**µ**_**BNF,obs**_	**hr**^**-1**^	**0.044**	**0.010**	Diazotrophic growth rate, experimental observation	This study
*Q*_*N,BNF*_	µ*mol*_*N*_*.ODmL*^*-1*^	*0.9*	*[0.6–1.5]*	Calculated as v_max,BNF_ / µ_BNF,obs_	This study
Q_N,BNF_	µmol_N_.ODmL^-1^	1.62	0.14	Estimated from ARA experiments as dNfix/dOD_BNF_ (SI Dataset S1, VBNF Tab)	This study
Y_BNF_	ODmL	3.8	0.2	Maximal biomass yield (from OD_600_) for 30 mM of pyruvate under deplete NH_4_^+^ conditions	This study
*Pyr_BNF_ratio*	*mol*_*Pyr*_*.mol*_*N*_^*-1*^	*88*	*[49–131]*	*Calculated for 30 mM of pyruvate using Y*_*BNF*_*and Q*_*N,BNF*_	*This study*
*SO4_BNF_ratio*	*mol*_*SO4*_*.mol*_*N*_^*-1*^	*22*	*[12.8–33]*	*Calculated as Pyr_BNF_ratio/ Pyr_SO4_ratio*	*This study*
SO4_OD_BNF	µmol_SO4_.ODmL^-1^	18.9	0.6	H_2_S_tot_ produced (SO_4_ reduced) per ODmL during N_2_-trophic growth	This study

Bold values were used as fixed parameters in the DAD-growth model. Italicized values were calculated from the fixed parameters. Parameters appearing twice indicate values from two independent calculations or sources. Biomass was expressed as ODmL, or the number of cells contained in 11 mL of culture at OD600=1. = 1.

The main features of batch culture experiments with *Dv*H under sub-replete initial ammonium concentrations ([NH_4_^+^]_initial_) (<2300 µM, 30 mM of Pyruvate, 10 mM Sulfate, [11]) are summarized in Fig. 1A. Cultures exhibited a clear bi-phasic growth, with the initial growth phase supported by ammonium uptake (first ∼ 75 h.) followed by a second, diazotrophic growth phase. The two growth phases were separated by a lag phase (>24hrs., Fig. 1A), indicating the diazotrophic regime could be modelled independently from the ammoniotrophic regime as a second, separate exponential growth curve. Hence, in our model formulation, the total active cell number (Cell_tot_, expressed in ODmL) can be separated into two cell groups with distinct functional characteristics. One group consists of idealized specialized N-fixing cells (Cell_BNF_, expressed in ODmL), which contain active NH_4_^+^ transporters and a fixed and optimal number of nitrogenase enzyme required to support maximal BNF activity. The second group consists of ammoniotrophic cells (Cell_NH4_, expressed in ODmL), which only possess NH_4_^+^ transporters and do not have diazotrophic capability, (i.e., ${\mathrm{Cell}}_{\mathrm{tot}}={\mathrm{Cell}}_{\mathrm{BNF}}+{\mathrm{Cell}}_{\mathrm{NH}4}$) (Fig. 1B). All cells can uptake NH_4_^+^ and contribute to the increase in the Cell_NH4_ pool, but only N acquired through BNF contributes to the Cell_BNF_ pool (Table 1). This mathematical simplification facilitated the quantification of biomass produced by each N source (ammonium or dinitrogen) and the parameterizations of each growth mode using purely diazotrophic (initial [NH_4_^+^]<10 µM) and ammoniotrophic (initial [NH_4_^+^]>3000 µM) experimental data.

At each time increment (dt), Cell_BNF_ and Cell_NH4_ pools acquire nitrogen according to the pathway described above and time-dependent enzyme-specific activities for nitrogen fixation and ammonium transport (see Table 1). The amount of each specialized biomass type (i.e., Cell_BNF_ and Cell_NH4_) is increased using cell-specific cellular N quotas (Q_N,BNF_ and Q_N,NH4_, both expressed in µmol_N_.ODmL^-1^, for diazotrophic and ammoniotrophic cells, respectively), and the composition (NH_4_^+^, SO_4_^2-^, and pyruvate) of the surrounding medium is then balanced to account for the increase in biomass and by removing stochiometric amounts of sulfate and pyruvate according to measured and theoretical stoichiometries (Fig. 1 and Table 1).

To study the dynamics of N fixation activity following NH_4_^+^ addition, we used acetylene reduction assays (ARA), a common method to measure nitrogenase activity [24]. To allow the model to integrate data from ARA experiments, we accounted for the use of acetylene as a nitrogenase substrate in diazotrophic cells. We assumed total nitrogenase activity was partitioned between acetylene reduction and nitrogen reduction according to nitrogenase acetylene saturation level (Sat = [Ac]/([Ac]+K_M,Ac_), expressed in %, where [Ac] is acetylene headspace concentration in %v/v, and K_M,Ac_ the Michaelis-Menten constant of nitrogenase for acetylene, in %v/v). Hence, during ARA experiments, diazotrophic cells acquire nitrogen to support the increase of the Cell_BNF_ pool at only (1-Sat) % of the measured nitrogenase activity $v_{\mathrm{BNF}}$ (see Table 1). According to measured delays in ammonium inhibition [11], we incorporated in the model a 30 min delay between NH_4_^+^ addition and the occurrence of the transition between diazotrophy and ammoniotrophy.

In a first set of model simulations, the DAD-GM was applied to reproduce batch culture growth data using the initial media conditions of our laboratory experiments (see below) with increasing [NH_4_^+^]_initial_ (0–3000 µM, 30 steps). The DAD-GM was iterated for a total simulated time of 300 h with a time step (dt) of 0.5 h (600 steps). We performed a second type of simulation to investigate growth, BNF activity, and ammonium uptake during the addition of increasing [NH_4_^+^] (0–3000 µM) to actively growing diazotrophic culture under acetylene reduction conditions. To facilitate comparison with laboratory experiments, where volume could change with media sampling, results for cell number are expressed as cell density (OD_600_). The model and figures were created in R studio 2022.07.1 with R version 4.2.1 (See SI RMarkdown Code). All data and code used in this article are available at 10.6084/m9.figshare.22800980.

### 2.2 Growth medium preparation

*Desulfovibrio vulgaris* var. Hildenborough (ATCC 29579, [25]), termed DvH, was grown anaerobically in a minimal diazotrophic medium for sulfate reducers [52] in 27 mL Balch tubes (10 mL medium) with 20 mm butyl rubber septum stoppers (Bellco glass, Vineland, NJ, USA) as described in [11]. Briefly, the medium was prepared using standard anoxic procedures and a glovebox (2% H_2_, 98% N_2_) (Coy Laboratories, Grass Lake, MI, USA). Sulfate (Na_2_SO_4_) was provided as an electron acceptor at a replete concentration (>10 mM). Either pyruvate (30 mM) or lactate (20 mM), used as the electron source, was the growth limiting nutrient in batch cultures. Molybdenum was provided as sodium molybdate dihydrate (Na_2_MoO_4_.2 H_2_O) at 0.2 µM and iron as iron (II) chloride tetrahydrate (FeCl_2_. 4 H_2_O) at 5 µM, a concentration at which we did not observe FeS_(s)_ precipitation.

### 2.3 Batch culture growth experiments and biomass measurements

We conducted growth experiments containing different initial concentrations of ammonium (NH_4_^+^), which ranged from background concentration (<10 µM) to 3000 µM. Ammonium was added as ammonium chloride to the medium from 10- to 100-fold stock solutions prepared in aseptic culture medium to reach the required final initial medium concentration. Growth experiments were initiated at an initial OD_600_ ∼ 0.05 by needle addition of 100–300 mL of a starter culture grown under the same conditions for at least 10 generations. Cells were grown at 30 ^o^C in the dark, with tubes placed obliquely in an orbital shaker at a constant agitation speed of 130 rpm. Culture cell density was followed by turbidimetry at 600 nm using a Thermo Fisher Scientific (Waltham, MA, USA) GENESYS spectrophotometer equipped with a tube holder. Each experiment was performed in at least triplicate. The typical results of our growth experiments are summarized and explained in Fig. 1A.

### 2.4 Acetylene reduction assay experiments

Data from experiments using the Acetylene Reduction Assay (ARA) described in a prior publication [11] were used to calibrate nitrogenase maximal specific activity (v_BNF,__MAX_) and to validate ethylene production, growth, and ammonium uptake under the ARA condition. Briefly, the experiments consist of diazotrophic cultures of DvH in pyruvate (30 mM) or lactate (40 mM) with replete sulfate as the electron acceptor (20 mM). Once cultures reached an OD_660_ ∼ 0.08 ± 0.01, the ARA experiments were started by adding acetylene in the headspace. Since acetylene directly inhibits nitrogen fixation, we used two different acetylene concentrations depending on the experimental needs. During experiments designed for calibrating nitrogenase maximal specific activity, we employed a close to saturating acetylene condition for molybdenum nitrogenase according to available kinetic data (5.9% v/v, [10]) and pyruvate, a fast-growing substrate, to ensure high rates of ethylene production and thus reduced measurement uncertainty. Conversely, we used a lower acetylene concentration (1.7% v/v) in a second type of experiment with lactate, a slower growing substrate, to reduce the acetylene inhibition of nitrogen fixation, ensure maximal cell growth, and allow sufficient time to conduct sampling at frequent timepoints. In all experiments, once we confirmed ethylene production was active (after ∼ 3 h), ammonium was introduced as a single addition into actively fixing cultures (final concentration 10–3000 µM) and headspace aliquots from each culture were sampled for the next 100–120 h and analyzed for their ethylene content (see [11] for additional details).

### 2.5 Calibration of diazotrophic growth phase in the model with Acetylene Reduction Assays

Nitrogenase maximal specific activity (v_BNF,MAX_) was estimated in pyruvate experiments conducted with ∼5.9 %v/v acetylene headspace concentration ([Ac]), assuming an enzyme saturation (Sat) of 60% (with a K_M_,_Ac_ ∼ 0.04 atm, [12]) and a theoretical R ratio (i.e., the ratio of acetylene to dinitrogen reduction rate) of 3.2 for the Mo nitrogenase [2]. Values were averaged across replicates for each timepoint, leaving out conditions in which ammonium was present in the media and the last time point when growth reached stationary phase, for a total of 16 individual cultures and 51 measurements over four timepoints (40 ± 18 nmol_N_.hr^-1^. ODml^-1^, range 25–65 nmol_N_.hr^-1^. ODml^-1^, SI Dataset S1, VBNF tab).

### 2.6 Measurement of sulfate utilization during growth

The quantity of sulfate required during diazotrophic and ammoniotrophic growth in this study was estimated by measuring dissolved hydrogen sulfide concentrations during and at the end of growth experiments using the methylene blue method [8]. At multiple times along the experiment, at the end of visible growth, (i.e., maximal biomass yield), and during the observed decay phase (∼48 hrs. after maximal yield), aliquots of medium were sampled anaerobically using needle and syringe, filter sterilized, and preserved in a 2% zinc acetate solution at 4 ^o^C for< 7 days until measurement. The extinction coefficient was obtained from external calibrations. The calibration (slope=0.028 ± 0.0003 mmol.L^-1^.cm^-1^, range=0.1–6 µM, dilution rate 250X, n = 5) was obtained using sodium sulfide nonahydrate (Sigma Aldrich, Allentown, PA, USA) prepared in a serum bottle sealed with a 22 mm blue butyl septum. A selected crystal of appropriate mass was properly cleaned with MilliQ® water (Sigma Millipore, West Springfield, MA, USA), thoroughly dried using absorbent paper, weighed, and dissolved in a known volume of MilliQ® water. Total H_2_S (H_2_S_tot_) produced (a proxy for total SO_4_^2-^ used during growth) was calculated by correcting HS^-^_aq_ for partition between chemical species (S^2-^, HS^-^, and H_2_S, pKa_1_ =7) and neglecting dissolved H_2_S_(g)_ using Henry’s constant in water Hcc (water/air) = 0.0036 [47], at 30 ^o^C and pH 7.1 (final correction factor applied: HS^-^_aq_ / H_2_S_tot_= 0.44). For each replicate, the highest H_2_S_tot_ value measured was used to calculate the mean value of the condition.

## 3 Calculation

### 3.1 Model parametrization using experimental data

We estimate the key parameters of the model from experimental data using regression analyses between biomass production and sulfate usage (Fig. 2A), biomass decay in relation to biomass density (Fig. 2B), and sulfate and nitrogen utilization during growth (Fig. 2C&D) for cultures containing increasing initial ammonium concentration ([NH_4_^+^]_init_). Sulfate usage for growth was 18.9 and 9.7 SO_4_^2-^.ODmL^-1^ for BNF- and NH_4_^+^-derived biomass, respectively (Fig. 2A and Table 2). We estimate r_death_ (in hr^-1^), the rate at which the biomass was removed from the media due to senescence or death (see Fig. 1A and Table 1), assuming a decay rate directly proportional to cellular density (r_death_=0.0025 hr^-1^, Fig. 2B).

**Fig. 2:**
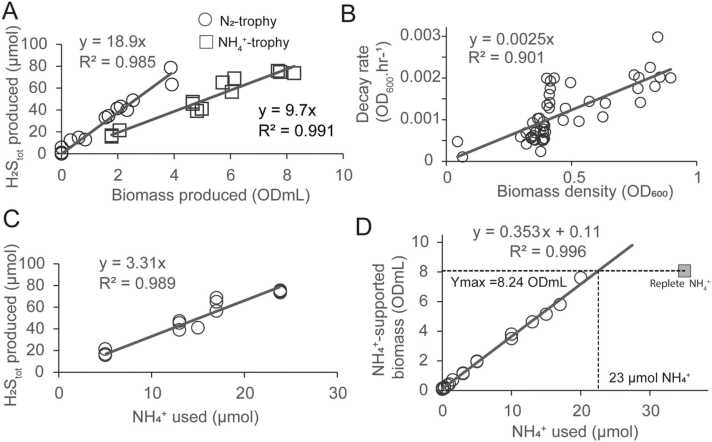
**Calibration of DAD-GM growth parameters using DvH batch culture data.** Panel (A) shows sulfate usage measured as H_2_S_tot_ per biomass produced for diazotrophy (black circle) and ammoniotrophy (grey square) along growth for two replicates. Panel (B) shows the relationship between the decay rate (calculated from the regression of biomass with time during the decay phase) and maximum biomass reached during growth. Panel (C) shows the relationship between sulfate and ammonium usage at the first growth plateau, ie., at the end of ammoniotrophic phase (see Fig. 1A). Panel (D) shows the relationship between biomass at the ammoniotrophic growth plateau and initial ammonium present in the medium. The horizontal dotted-line indicates maximal biomass reached under replete condition of NH_4_^+^ and its intersection with the regression line indicates stochiometric use of ammonium under limiting 30 mM pyruvate condition (vertical dotted line). All data are available in SI Dataset S1.

We estimated ammonium and sulfate stoichiometry (3.31 + 0.1 mol_SO4_.mol_NH4_^-1^) from the regression of [NH_4_^+^]_init_ versus maximum SO_4_^2-^ reduced (as H_2_S_tot_ equivalents, see Fig. 2C). Pyruvate to ammonium stoichiometry was calculated to be 13.0 + 1.4 mol_Pyr_.mol_NH4_^-1^, based on the ∼ 2.3 mM NH_4_^+^ required for stoichiometric balance under our growth-limiting initial 30 mM pyruvate conditions (i.e., line intersection in Fig. 2D).

The nitrogen growth quota for ammoniotrophic-derived biomass (Q_N,NH4_) was estimated as the slope between the biomass yield from ammoniotrophic growth (first growth plateau, see Fig. 1A) and [NH_4_^+^]_init_ (e.g., Q_N_,_NH4_= 1/0.353 = 2.8 µmol_N_.ODmL^-1^, Fig. 2D). Maximal specific ammonium transport rate (V_NH4,Max_) was then calculated using the measured growth rates of NH_4_^+^ replete cultures (µ_NH4_=0.105 hr^-1^, [NH_4_^+^]_init_ =3000 µM, n = 3, Table 2) and our estimate for Q_N,NH4_, with measured growth rates corrected for the decay rate (r_death_), according to Droop’s cell quota relationship (Eq. 3, [16]) applied to ammoniotrophy.

(3) $$V_{i,\max}=Q_{i,\mathrm{measured}}\times \left(\mu_{i,\mathrm{measured}}+r_{\mathrm{death}}\right)$$

Nitrogen biomass quota for N-fixing cells (Q_N,BNF_) was calculated using diazotrophic growth rate (µ_BNF_=0.044 hr^-1^, [NH_4_^+^] _init_ ∼ 0 µM, n = 3, Table 2) and average nitrogenase specific activity (v_BNF,max_ = 42 +22 nmol_N_.hr^-1^.ODmL^-1^, range 25–65 nmol_N_.hr^-1^.ODmL^-1^, Table 2, SI Dataset S1_VBNF tab) according to Eq. (3) applied to diazotrophic growth and was found to be Q_N,BNF_ = 0.9 ± 0.5 µmol_N_.ODmL^-1^ (range 0.6–1.4, Table 2, SI Dataset S1, VBNF tab).

### 3.2 Validation of calibration parameters

We confirmed the validity of the model parameters and output using independently measured variables as well as theoretical and literature values. Our independent estimates for SO_4_^2-^:NH_4_^+^ use (3.31 ± 0.1 mol_SO4_.mol_NH4_^-1^, based on direct H_2_S measurements, Fig. 2C) and Pyr:NH_4_^+^ (13.0 ± 1.4 mol_Pyr_.mol_NH4_^-1^, derived from the growth data for 30 mM Pyruvate with 2.3 mM NH_4_^+^ used at stoichiometry, Fig. 2D) produce a calculated ratio of 4.0 ± 0.5 mol_Pyr_.mol_SO4_^-1^ (Table 2) in full agreement with the stoichiometry of 4 mol_Pyr_.mol_SO4_^-1^ expected for the incomplete oxidation of pyruvate to acetate during growth of *Dv*H with pyruvate as the electron donor [52]. Similarly, we used our estimates for biomass-specific SO_4_^2-^ usage (18.9 and 9.7 µmol_SO4_. ODmL^-1^, based on direct H_2_S measurement during growth, Fig. 2A) and the maximal biomass yield constrained by stoichiometrically-limiting [Pyr]_init_ ∼ 30 mM (3.8 vs. 8.3 ODmL, Table 2) to re-calculate [Pyr]_init_ and associated uncertainties (28.7 ± 1.8 and 31.9 ± 1.5 mM, see SI Supplementary Material Methods S1) for diazotrophic and ammoniotrophic growth, respectively. The resulting calculated values are again in good agreement with the theoretical concentration used in experiments, with a maximal error of ∼ 10% between the two sets of values (i.e., 32 and 29 mM calculated vs. 30 mM pyruvate theoretical). Altogether, these results validate our assumptions on H_2_S speciation used to calculate SO_4_^-^ usage data.

We directly estimated Q_N,BNF_ from the ARA experiments, by dividing the total amount of N fixed during the experiments in each individual culture (dmol_N_, assuming 40%, or 1–60% saturation, for enzyme activity toward N fixation, SI Dataset S1, QNBNF tab) by the total biomass created corrected for ammonium contribution (dODmL). The resulting value was at the higher end of the calculated estimate using v_BNF,max_ (1.62 + 0.14 vs. a range of 0.6–1.5 umol_N_. ODmL^-1^, see Table S1, SI Dataset S1, QNBNF tab).

Finally, we estimate the amount of growth inhibition to DvH due to use of the 5.9%v/v acetylene in ARA experiments by calculating dln(OD)/dt for every timepoint and each individual culture, removing the first timepoint and conditions under which ammonium was present. The average growth rate under 5.9%v/v acetylene led to a value of 0.018 ± 0.007 hr^-1^ (SI Dataset S1, Mu_ARA tab), or about ∼ 41% of the diazotrophic growth rate under non-ARA conditions. This result supports our assumption of a 60:40 partition of nitrogenase total activity between acetylene reduction and nitrogen fixation and the validity of the K_M,Ac_ value used here (i.e., K_M,Ac_ = 4%v/v, Table 2).

### 3.3 Evaluation of model goodness-of-fit

**Fig. 3:**
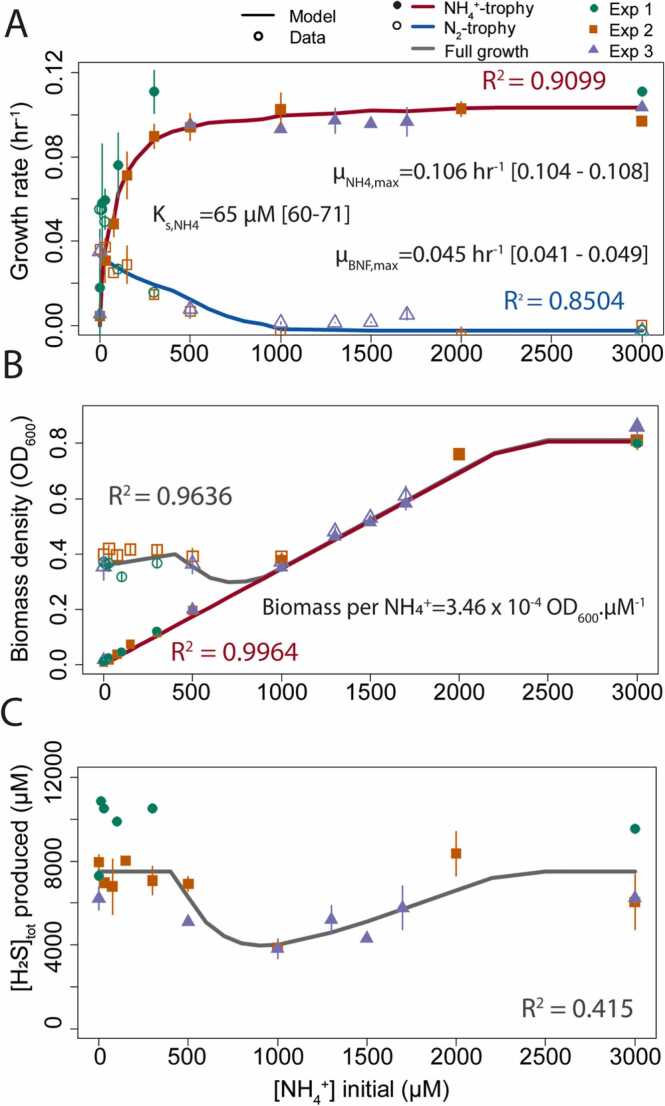
**Evaluation of modeled and experimentally observed growth rates, biomass yields, and maximal [H**_**2**_**S]**_**tot**_**produced for batch culture of DvH grown with varying [NH**_**4**_^**+**^**]**_**init**_**.**Experimental data were acquired from three independent replicates conducted under similar conditions (30 mM Pyruvate and 10 mM Sulfate) with increasing [NH_4_^+^]_init_. Panel (A) shows the evolution of the growth rate for diazotrophic and ammoniotrophic phases, Panel (B) shows the maximum biomass obtained at the first growth plateau (i.e., under ammoniotrophic condition) and at the end of growth, and panel (C) shows maximum [H_2_S]_tot_ concentration produced during growth, a proxy of sulfate usage. Values and uncertainty associated with experimental maximal growth rate (µ_NH4,max_ and µ_BNF,max_), Monod constant for NH_4_^+^ (Ks,_NH4_), and nitrogen specific biomass yield (Biomass.mol_N_^-1^) obtained from regression analyses of experimental data are shown. Error bars are SD.

**Fig. 4:**
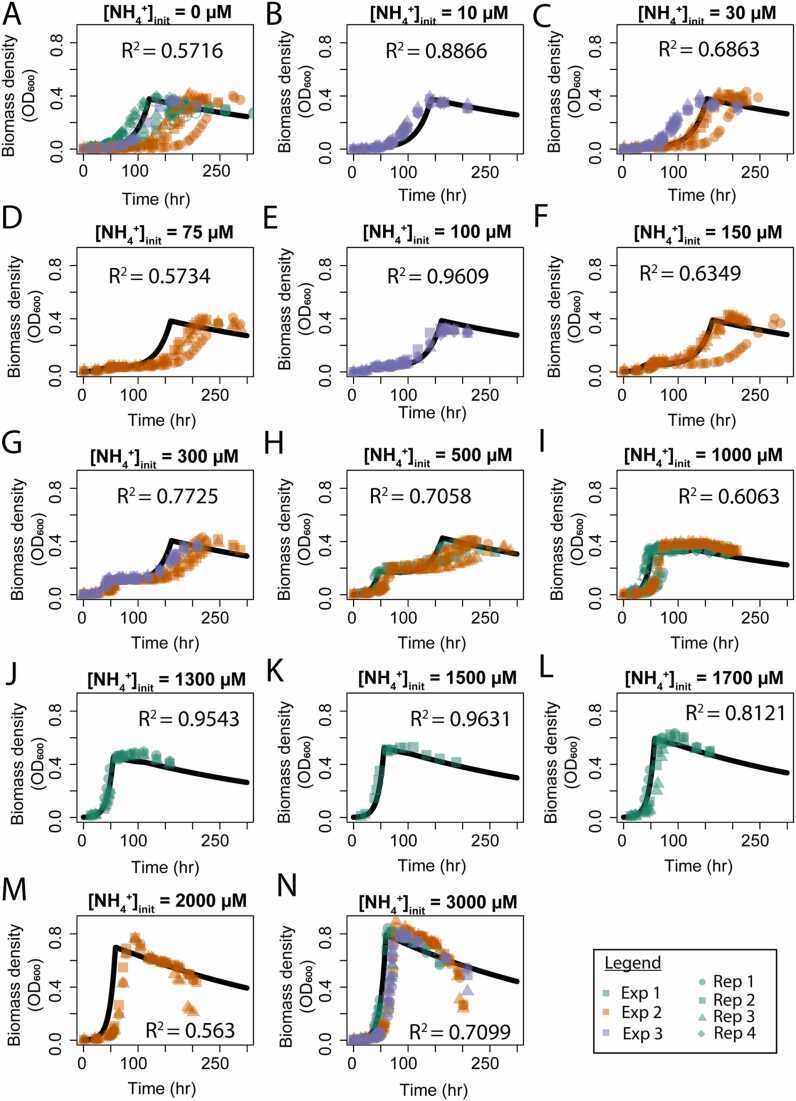
**Evaluation of DAD**-**GMsimulated and experimentally observed growth curves for DvH** from three independent experiments (Exp 1–3, minimum 3 technical replicates for each condition within experiments) with variable initial ammonium concentration (0–3000 µM).

**Fig. 5:**
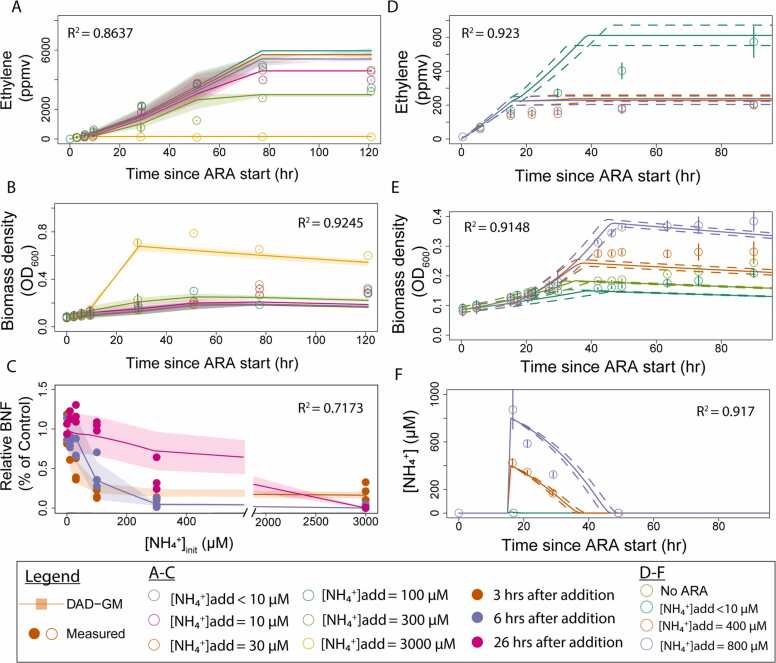
**Evaluation of DAD-GM model for ammonium additions to diazotrophically grown DvH (**A-C) were experiments conducted on pyruvate media (30 mM, n = 3, [Acetylene]=5.9%v/v) and (D-F) were conducted on lactate media (20 mM, n = 3, [Acetylene]=1.7%v/v). (A) and (D) show ethylene concentration in the headspace, (B) and (E) show biomass of *Dv*H. (F) shows the evolution of [NH_4_^+^] in media. Panel (C) shows BNF contribution to biomass growth, as calculated in [10]. In panel (E), “No ARA” condition refers to growth without acetylene in the headspace. Uncertainty bands (filled and dotted) represent model output for the range of initial biomass amounts used in the laboratory experiments (OD_600_ = 0.07–0.09).

To assess the performance of the DAD-GM model, we evaluated the goodness of fit using a linear regression of the model output with experimental data for each [NH_4_^+^]_init_ condition (Fig. 3) and for each measurement time (Fig. 4&5). For each figure and each variable, we report the goodness-of-fit as the adjusted R-squared of the regression (R^2^).

## 4 Results

### 4.1 DAD-GM simulations of DvH growth under varying levels of ammonium repleteness

To evaluate DAD Growth Model performance, we first compared modeled and measured growth rates, biomass yields (reported as maximal biomass density in 10 mL cultures), and maximum H_2_S_tot_ produced in batch cultures of DvH at increasing [NH_4_^+^]_init_ (Fig. 3). Modeled growth rates for the ammoniotrophic and diazotrophic phase were calculated using an approach similar to that applied to laboratory data (i.e., linear regression of the logarithm of the total active cell, Fig. 3A), and we extracted the maximal biomass yield for ammoniotrophic growth (i.e., in relation to the first growth plateau, see Fig. 1A) and complete growth (maximum of Cell_tot_) (Fig. 3B).

Maximum produced [H_2_S]_tot_ in the model was also compared to measured values at the end of visible growth (i.e., maximal biomass yield) and in multiple cases 48 h later (Fig. 3C). The model and experimental data for growth rate and final and NH_4_ plateau biomass yield showed close agreement with each other (R^2^>0.85), and modeled [H_2_S]_tot_ values reproduce well the average measured [H_2_S]_tot_, albeit demonstrating higher variability (R^2^ = 0.41).

Assuming the growth experiment was started with fully competent N_2_-trophic cells at an initial biomass density (set to 0.002 OD_600_ (i.e., for 10 mL culture, Cell_BNF,0_ = 0.02 ODmL and Cell_NH4,0_ =0 ODmL), simulations yielded a good fit between the measured and modeled diazotrophic and ammoniotrophic growth rate for [NH_4_^+^]_init_ ranging from 0 to 3000 µM (Fig. 3A, R_2_>0.85). Changing values of K_M,NH4_ between 0 and 20 µM, within the range of published K_M_ values for diazotrophs (0–20 µM, [35]), produced similar results. These K_M_ values are of similar magnitude to the measured threshold value for nitrogenase inhibition found by [10] for *Dv*H in pure culture and in slurry incubation ([NH_4_^+^]_threshold_ ∼10 µM) and used as K_i,NH4_ used in this study (Table 2). Moreover, the Monod constant for ammonium K_s,NH4_, estimated using non-linear regression of our simulated growth rate data, was found to be K_s,NH4_ = 32 µM ± 4 µM, slightly higher than the preset value of K_M,NH4_ of 20 µM within the model (see Fig. 3A).

In addition to the good performance of the model for quantifying cellular parameters and medium final composition (R^2^>0.85 for growth rate,>0.95 for biomass density, and ∼ 0.4 for [H_2_S]_tot_), the DAD Growth Model closely reproduces (0.56 <R^2^<0.96) biomass increase with time for increasing [NH_4_^+^]_init_ across three independent replicates and using the exact same starting condition (i.e., the same initial biomass quantity of ammoniotrophic and diazotrophic cells). Good visual agreement between modeled and measured data is particularly evident during ammoniotrophic growth phases (see Fig. 4 H-N), but there are some disparities for the diazotrophic growth phases, particularly at low initial [NH_4_^+^], which we mostly attributed to fluctuation in the length of the first (see Fig. 4M) or second lag-phase between NH_4_^+^- trophic and N_2_-trophic growth (see Fig. 4 A-G for [NH_4_^+^]_init_ 0–300 µM). Importantly, our model was able to reproduce the observed stationary phase before decay for [NH_4_^+^]_init_ between 1000 and 1500 µM (see Fig. 4 I&J).

### 4.2 DAD-GM simulations of DvH diazotrophic growth response to ammonium addition

To further evaluate model capability, we tested whether the DAD-GM could reproduce growth dynamics after ammonium addition to diazotrophically growing DvH, as recorded using ARA experiments in which ammonium was added to growing cultures at various concentrations (0–3000 µM, Fig. 5).

The modeled ethylene production (Fig. 5A, R^2^ =0.86), growth curves (Fig. 5B, R^2^ =0.92), and dynamics of N fixation inhibition (Fig. 5C, R^2^ =0.71) generally match the measured data from pyruvate experiments used to calibrate V_BNF_ ([11]). We also applied the same DAD-GM to evaluate a similar experiment using lactate instead of pyruvate as the electron donor (40 mM), and a change in the electron donor: sulfate ratio to fit the stoichiometry of lactate (2:1 instead of 4:1, [43]), and the growth rate of the diazotrophic and ammoniotrophic phase (µ_BNF,lac_=0.022 hr^-1^ and µ_NH4,lac_=0.044 hr^-1^). In both the modeled and measured data, ethylene production, culture growth and ammonium consumption showed similar patterns (Fig. 5 D, E &F, R^2^>0.9). Of note, there was a 5–10 hrs delay in modeled and measured ethylene production (Fig. 5D). Conversely, the model shows a slower and delayed NH_4_^+^ uptake than our measurements for the 800 µM NH_4_^+^ addition experiment (Fig. 5F). In both cases, the delay was not observed in biomass data (compare Fig. 5 D, E, and F). In addition, contrary to the model assumption, there was no visible decay phase in the lactate condition (Fig. 5E).

### 4.3 DAD-GM identification of N fixation specific growth limitation

To be able to model the decrease in maximum H_2_S_tot_ produced in experiments with [NH_4_^+^]_init_ between 500 and 1500 µM (Fig. 3C), it was necessary to arbitrary impose a limiting step preventing BNF activity in the model. Indeed, under nitrogen deplete conditions, measurements indicate cultures appear to stop growing once total biomass (i.e., ammonium and BNF- supported) reached a density of OD_600_ ∼ 0.4 (compare Fig. 3B and C and see Fig. 4). We could not identified the metabolic process underpinning this limitation. It was thus introduced in the model as a condition on the total amount of dead biomass: “no BNF activity once total dead biomass density is over x OD_600_”, and was tuned independently in simulations of experiments with and without acetylene to achieve the best fit between model and data (0.03 and 0.065 OD_dead_ with and without acetylene, respectively).

## 5 Discussion

### 5.1 Metabolic perspectives on cost of nitrogen acquisition: NH_4_^+^ versus N fixation

The measured experimental data, supported by the overall good goodness-of-fit of the DAD-GM (R^2^>0.85 for growth rates,>0.95 for biomass density yields, ∼ 0.4 for [H_2_S]_tot,_>0.56 for growth curves,>0.85 for NH_4_^+^ addition experiments), enable comparison of the metabolic cost of diazotrophy vs. ammoniotrophy in DvH (Table 3). Estimates of ATP cost per unit of biomass can be calculated using direct measurements of sulfate usage per biomass produced (18.9 and 9.7 µmol_SO4_. ODmL^-1^, Table 3) for BNF and NH_4_ -supported growth, respectively. Assuming 1 mol of SO_4_^2-^ used per mol ATP produced [5], diazotrophy-derived biomass requires twice as much ATP as NH_4_^+^-derived biomass (18.9 vs. 9.7 µmol_ATP_. ODmL^-1^). Assuming 5.5 × 10^8^ cell.ODmL^-1^ (as found at 600 nm for *Desulfovibrio desulfuricans*, [59]), ∼ 20 billion and 10 billion ATP molecules are required for one cell made under diazotrophic and ammoniotrophic conditions, respectively. In comparison, *Escherichia coli*, a non-diazotrophic organism of similar shape but smaller than DvH, requires ∼12–20 billion ATP to synthesize one cell (range for various growth conditions, [53]), indicating DvH could have a relatively lower biomass cost than this well-known model organism.

**Table 3:** Comparison of metabolic costs and growth outcomes between ammoniotrophic (NH4) and diazotrophic (BNF) growth of DvH, an anaerobic heterotrophic diazotroph.

Parameters	Unit	NH4		BNF	SD	Ratio (NH4/BNF)	Method
ATP/Biomass	µmol_ATP_. ODmL^-1^	9.7	0.3	18.9	0.6	0.5	Measured
ATP/N	mol_ATP_.mol_N_^-1^	3.3	0.3	21 (12–32)	3	0.15	Calculated
µ (corrected)^#^	Hr^-1^	0.114	0.005	0.044	0.01	2.6	Measured
Y_Max_(corrected)^#^	(ODmL)	10.6	0.3	4.0	0.2	2.1	Measured
Q_N_	µmol_N_. ODmL^-1^	2.8		0.9 (0.6–1.6)		3.1	Measured

^#^ Values corrected for biomass decay.

When the overall metabolic cost was reported per mol of N using the N biomass requirement (Q_N,BNF_ = 0.9 and Q_N,NH4_ = 2.8 mol_N_.ODmL^-1^, Table 2), we calculated an average cost for BNF ∼ 21 ± 3 mol_ATP_.mol_N-BNF_^-1^ (range between 13 and 32 mol_ATP_.mol_N-BNF_^-1^), almost three times larger than the direct biochemical cost of 8 mol_ATP_.mol_N-BNF_^-1^ for nitrogen fixation (i.e., 16 mol ATP per mol of N_2(g)_ reduced, [50]). Direct and indirect costs associated with BNF growth, of a similar order of magnitude, have been found in cyanobacteria and aerobic heterotrophs, due to large energy expenditure associated with oxygen protection mechanisms [23], [27]. The indirect cost for BNF under anaerobic conditions is similarly quite substantial and cannot be due to oxygen, but likely illustrates fundamental indirect costs associated with the synthesis of complex enzymatic machinery required to undertake BNF, such as the higher N content of nitrogenase enzyme versus typical ammonium transporters (∼ 5:1 N:protein_BNF/NH4_), other enzymes required for nitrogenase co-factors maturation and nitrogenase regulation, or the requirement for essential metal uptake (Mo, Fe) [1], which could become more costly under anaerobic and sulfidic conditions due to the lower solubility of some metal chemical forms.

The value for Q_N,BNF_ used to estimate these costs, calculated from growth rate and nitrogen specific activity ranging from 0.6 to 1.5 µmol_N_.ODmL^-1^, is likely an underestimate of the true net cellular N quota for N_2_-trophic derived biomass. First, another estimate using accumulated N-fix and total biomass increase during the entire ARA experiment yields a slightly higher value at 1.6 µmol_N_.ODmL^-1^, with a corresponding metabolic cost of 12 mol_ATP_.mol_N-BNF_. Second, both estimates for Q_N,BNF_ were obtained under strong N limitation due to the 60% inhibition of N fixation by acetylene, as evidenced by the lower observed growth rates during the experiments (0.018 v. 0.044 hr^-1^). So while our parameters allow good *in silico* reproduction of the ARA experiments, it is likely that cells decrease their N cellular quota to help cope with nitrogenase interactions with non-N_2_ substrate. In addition, our model does not consider fluctuations in biomass per cell or cell size between the different conditions, which could result from a lower N quota (i.e., change in cell. ODmL^-1^ values). Finally, electron flux to nitrogenase was assumed to be separate between N reduction and AR in a constant sum, but as two binding sites for acetylene exist [33], it is likely that the sum of assumed BNF and ARA rates is not equal to total BNF activity in the absence of acetylene. In addition, the competition between acetylene and nitrogen is dependent on the ratio of the two sub-units of nitrogenase (nitrogenase reductase and dinitrogenase), which could vary *in vivo* [12]. Hence, it is likely that the actual Q_N,BNF_ is at the higher end of our estimate (<1 µmol_N_.ODmL^-1^) and that cost of BNF is< 20 mol_ATP_.mol_N_. Notwithstanding these study’s limitations, our results still indicate a significant indirect cost of 4–12 additional ATP.mol_N_^-1^ for BNF under anaerobic conditions.

Our estimate for Q_N,NH4_ (2.83 ± 0.04 µmol_N_.ODmL^-1^) is very close to the value found in the literature (2.95 µmol_N_.ODmL^-1^, [42]). The net cost of ammonium uptake is 3.3 ± 0.3 mol_ATP_.mol_N-NH4_^-1^, three times higher than the direct cost of ion transport through the membrane [31]. The mechanism of NH_4_^+^ transport through the cell membrane has been highly debated over the last 20 years [32], [34], [58], [61]. One possibility would be that NH_4_^+^ is transported as a charged ammonium ion requiring the cell to pump out one ion at the cost of 1 ATP to retain the electrochemical potential of the cell. The other possibility is that NH_4_^+^ transport occurs in an electro-neutral form as ammonia (NH_3_), with possibly no ATP cost [32]. Assuming a cost of 1 ATP for NH_4_^+^ uptake, the remaining indirect metabolic cost for biosynthesis and maintenance of ammoniotrophic cells would be between 2 and 3 ATP per mol of biomass N, remaining lower than our lowest estimate for the indirect cost of BNF (4 ATP.mol_N_^-1^).

We attribute the two-fold difference between the ratio of ATP cost and observed growth rate for the two acquisition strategies (mol_ATP_.mol_N,__BNF_/mol_ATP_.mol_N,__NH4_ = 7 vs. µ_NH4_/ µ_BNF_ = 2.7, Table 3) to the difference in cellular N content between the two strategies (Q_N_ =0.9 vs 2.8 mol_N_.ODmL^-1^for BNF and NH_4_, respectively, ratio NH_4_/BNF = 3, Table 3). This stochiometric flexibility, in which BNF has a lower Q_N_ (most likely between 1 and 2 mol_N_. ODmL^-1^) would also explain the relatively higher maximal biomass yield ratio of diazotrophy vs. ammoniotrophy when compared to the ratio of growth rate and overall cost of BNF (Table 3). A calibration factor of 5.5 x10^8^ cell.ODmL^-1^(as estimated at 600 nm for *Desulfovibrio desulfuricans*, [59]) would yield values of 2 and 5 fmol_N_.cell^-1^ for N_2_-trophy and NH_4_-trophy, respectively. These values are in the range of Q_N_ found in the literature for pure culture non-diazotrophic organisms (2–7 fmol_N_.cell^-1^, [19], [56]), but are 3–4 times lower than values found for another model N fixer, *Azotobacter vinelandii*, grown under aerobic diazotrophic conditions (∼17 fmol_N_.cell^-1^, [1]). However, we note that *Azotobacter vinelandii* growth rate is> 5 times faster than *Dv*H under similar temperature conditions (0.04 vs. 0.2 hr^-1^ at 30 and 25 ^o^C, respectively, [1]). Hence, the high cellular quota of *A. vinelandii* can be attributed to the higher energy yield from oxygenic respiration (4–20 time more energy than sulfate respiration). This indicates that oxygenic respiration could allow *A. vinelandii,* an organism of similar size and shape to DvH*,* to afford higher N biomass content, even considering the large amount of energy spent in O_2_ protection strategies, such as wasteful respiration or production of a low permeability alginate layer [27], [46].

### 5.2 Model performance and limitations

Our model is able to resolve the main qualitative and quantitative features of heterotrophic anaerobic growth (e.g., growth rates, biomass yield, ammonium consumption) under a wide range of [NH_4_^+^]_init_ conditions using only a single set of initial parameters. The DAD-GM adds to the body of diazotrophic models that cover a variety of metabolisms, from aerobic heterotrophs to phototrophs [29], [30], [28], [45]. One of the key simplifying features of the DAD-GM is that its parameterization relies on activity measurements (e.g., nitrogen uptake or nitrogen fixation, sulfide production), which are easier to obtain than growth rate in environmental samples and would allow the model framework to be adapted to function in more chemically and physically complex conditions (e.g., sediment, soil cores).

The primary disparity between our modeled and measured data occurs in the diazotrophic regime of the model, particularly when reproducing growth curve dynamics. Modeled diazotrophic growth is generally faster than observations (see Fig. 4 A, D, and G), which show delayed or flattened growth. In addition, growth rates, biomass density yields, and decay rates in laboratory experiments were generally more variable during the diazotrophic phase than for ammoniotrophy (see standard deviations in Table 2, Fig. 3B, and Fig. 4 A,C,D, and F). There are several explanations that could contribute to these phenomena. Firstly, while all cells would be able to use ammonium at the beginning of the experiment, the initial amount of active nitrogenase enzymes in cells could fluctuate. Our measurements suggest there could be generally less of an initial active nitrogenase pool than what we arbitrary assumed for the model (Cell_BNF,0_ = 0.002). Thus, a better temporal fit between experimental data and model output could be obtained by adjusting Cell_BNF,0_ parameters for each experiment, or even for each replicate. Other explanations could stem from nitrogenases extreme sensitivity to oxygen [20] or possible competition between sulfate and oxygen as final electron acceptor [25], both of which could slow down some cultures and result in lower maximal biomass yields due to the associated metabolic cost of protective measures [27] (see SI Supplementary Material Fig. S1).

There is evidence of decoupling between N acquisition and biomass build up, as seen during the 5–10 hrs delay between measured and modelled [NH_4_^+^] uptake following addition that does not have much effect on the biomass growth (compare Fig. 5 E and F). This could be explained by the existence of a legacy intracellular N pool, which would support current growth. This phenomenon would warrant further characterization before being incorporated into the model.

### 5.3 Nature of the putative BNF-limiting phenomenon and its environmental significance

The plateau of maximum biomass density at OD_600_ = 0.38–0.4 obtained for [NH_4_^+^]_init_ between 0 and 1000 µM was an interesting and unexpected feature of our experimental results (Fig. 3B). Specifically, at [NH_4_^+^]_init_ = 1000 µM, and using the 2-fold measured difference in maximal biomass yield between ammoniotrophy and diazotrophy as found under purely diazotrophic and ammoniotrophic conditions (0.38 and 0.83 OD_600_, respectively, Table 2), a biomass density of OD_600_ ∼ 0.6 would be expected. However, the consistent findings of a lower OD_600_ yield indicate complexities associated with the dual growth mode induced by sub-replete NH_4_^+^ conditions. Notably, this biomass plateau occured at the same time as a decrease in maximal H_2_S_tot_ production for [NH_4_^+^]_init_ between 300 and 2000 µM (Fig. 3C), reaching its lowest value for [NH_4_^+^]_init_ = 1000 µM, indicating that not all pyruvate and sulfate were used under these conditions. We changed model parameters to better understand the processes involved in the yield plateau at sub-replete NH_4_^+^. While we were able to reproduce the maximal biomass yield plateau by changing the magnitude of biomass decay, it was not possible to reproduce the measured decreases in maximal H_2_S_tot_ concentration without the introduction of an arbitrary imposed limiting step specific to BNF that we only allowed to operate after a certain amount of biomass had been produced, or a certain amount of the existing biomass had died (compare SI Supplementary Material Fig. S2, Fig. S3, and Fig. 3C). While a change in the Pyr: SO_4_^2-^ ratio could have explained our results, we found a significant decrease in acetate, yielded from the incomplete oxydation of pyruvate, between [NH_4_^+^]_init_ = 0 and 1500 µM and a significant increase between 1500 and 3000 µM (SI Supplementary Material Fig S4). This indicates that stoichiometric flexibility, if present, could not account for all the observed features of our data (i.e., biomass yield plateau and maximal [H_2_S]_tot_ decrease), and points to the existence of a phenomenon that would specifically prevent diazotrophic growth in batch culture.

The existence of a limiting step that would only influence BNF and not NH_4_^+^ acquisition under SO_4_^2-^ and organic carbon replete conditions could have important implications for N cycling in the natural environment. It was thus important to better understand the nature of this limiting step. Trace metals Fe and Mo are required for nitrogenase synthesis and have complex chemistries under sulfidic conditions. We thus conducted additional laboratory experiments to test how initial or growth-induced sulfidic conditions could influence metal availability and help explain the BNF-only limitation. We first tested the effect of a two-fold increase in Fe and Mo with [NH_4_^+^]_init_ of 0 and 1000 µM. We tested similarly a two-fold increase in Ni, required for hydrogenase, and the removal of the initial background of 2% v/v H_2_, a direct inhibitor of BNF, using 100% N_2_ (SI Supplementary Material Fig. S5). In another experiment, we tested the addition of an initial H_2_S concentration of 4 mM (as Na_2_S), a strong metal complexing agent under anaerobic conditions [54], and the substitution of Fe^+2^ for a chelated and presumably less prone to precipitate form of Fe at the same concentration (Fe-EDTA, 2.5 µM) (SI Supplementary Material Fig. S6). We found no improvements in maximal measured yields or in growth rates at [NH_4_]_init_ = 0 µM, indicating neither direct metal availabilities nor the high concentration of H_2_S found at the end of growth or the inhibition of nitrogenase by H_2_ were obviously involved in this BNF limitation. In addition, in case of nutrient limitation, metabolic flexibility would generally allow growth to continue at a slower rate in a linear fashion (i.e., each subsequent doubling of cell halves the intracellular quantity of the limiting nutrient and reduces instantaneous growth rate), which we did not observed (Fig. 4). Toxicity from H_2_S could also be ruled out, as diazotrophy was able to support biomass production in N depleted cultures up to the stochiometric 7.5 mM H_2_S required to fully consume the initial 30 mM of pyruvate, and H_2_S produced at [NH_4_^+^]_init_ of 1000 µM was lower than this maximal (4 mM, Fig. 3C). Other potential explanations include continuous, slow diazotrophic growth or a long lag-phase overlaid by a faster decay phase. However, sequential measurement of H_2_S for [NH_4_^+^]_init_ of 1000 µM up to 100 hrs after the culture reached its maximal biomass yield showed very little increase in H_2_S production, and the maximal measured H_2_S value was used in Fig. 3C. As we were able to model the dip in H_2_S by imposing a constraint on total decayed biomass, we suggest that this phenomenon is either linked to the direct production of an inhibiting substance by DvH, or the indirect interaction of some substances (e.g., metals, metabolites) with unknown forms of organic matter [26], [9]. Further experiments are required to decipher the exact nature of BNF-specific limitation under the tested culture conditions.

### 5.4 Temporal and spatial aspects of nitrogen growth modes

The long transition time between the end of ammoniotrophic growth for ammonium adapted cells and the start of an exponential diazotrophic phase contrasts with the extremely fast resumption of BNF activity in BNF-adapted cultures upon depletion of any added NH_4_^+^, both in pure cultures and in benthic sediment slurries [11] These results indicate that the initial transition from ammoniotrophy to diazotrophy occurs under extreme N limitation, in which the build up of the nitrogenase enzyme pool could be itself strongly limited by the acquisition of new N. This interpretation is further supported by the rather sharp termination of the ammoniotrophic growth phase, without much of a slow-down before the complete exhaustion of NH_4_^+^ (see Fig. 4), the fast ammonium uptake following addition (see Fig. 5F), and the generally high affinity of the NH_4_^+^ transporter in the literature [35] and required in our model to fit our data (K_M,NH4_<20 µM). Accordingly, this however suggests that DvH is unable to sense ammonium depletion to onset nitrogenase synthesis prior to reaching extremely low [NH_4_^+^] (<1 µM). Under extreme N deplete condition, the second diazotrophic exponential phase can only be explained as either (i) the exponential self-reproduction from newly-fixed N of the initial pool of nitrogenase (i.e., from the inoculation) carried over by each cell following growth dilution during the ammoniotrophic phase, or (ii) by the replication of the first cells able to synthesize new nitrogenase enzyme (i.e., cell specialization), which would then have a strong competitive advantage to take over the population of cells. Thus, cells that could retain a pool of nitrogenase enzymes in a fluctuating environment in which [NH_4_^+^] remains low (0–20 µM) can strongly benefit from their initial resource investment. Such a phenomenon likely contributes to results from phenotypic heterogeneity experiments carried on with N fixers, which shows that single-cell BNF rates prior to N depletion correlates with their growth rates post limitation [49]. Conversely, constant exposure to high N loadings followed by N depletion would prevent *Dv*H from quickly shifting to diazotrophic growth in natural ecosystems.

The above considerations support segregating the total cells into ammoniotrophic and diazotrophic-competent cells in the model to efficiently reproduce macroscopic population level results. Indeed, for a unit of biomass created from both NH_4_^+^ uptake and BNF, it is not possible at the timescale at which experiments take place to macroscopically distinguish the biomass being created sequentially (i.e., first NH_4_^+^ uptake, then BNF), simultaneously in two different cells, or simultaneously in the same cells. This simple model would be a good candidate to include in more complex large-scale biogeochemical models, and is easily adaptable for spatially explicit models to help further our understanding of aspects related to marine biogeochemistry (e.g., particulate biogeochemistry and niche expansion, [6], [39]) or to microbial ecology (e.g., metabolic heterogeneity within a population at the single cell level, [49], [62]).

## 6 Conclusion

The Diazotrophic-Ammoniotrophic Dual Growth Model was able to accurately reproduce the growth dynamics of the anaerobic heterotroph sulfate reducer *Desulfovibrio vulgaris* Hildenbourough and its response to ammonium addition with two different substrates (pyruvate and lactate), using the same single set of parameters, and the additional adjustment of one parameter related to a diazotrophic-specific growth limitation and of the substrate sulfate usage stoichiometry. Our model was able to accurately reproduce the data of laboratory experiments, from growth rate, maximal biomass yield, sulfate consumption, and growth dynamics, as well as headspace ethylene increase and ammonium consumption during ARA experiments. In the future, the DAD-GM, which is built on simple metabolic relationships for ammoniotrophy and diazotrophy at the cellular level, represents an ideal model to study the behavior of N fixers under fluctuating environments in which ammonium may not be fully replete (e.g., due to diffusion of ammonium in sediment, or low frequency of fresh media supply in chemostat), and could easily be adapted to a spatially explicit or agent based models to investigate the micro-scale phenomena in physically and chemically complex environments. Additionally, our results demonstrate the existence of a putative limiting phenomenon that selectively hinders the activity of nitrogenase but not ammonium uptake under laboratory conditions. Should a similar phenomenon occur in nature, the potential of benthic ecosystems to obtain new nitrogen under N limitation could be reduced.

## CRediT authorship contribution statement

**Romain Darnajoux:** Conceptualization, Data curation, Formal analysis, Investigation, Methodology, Software, Validation, Visualization, Roles/Writing – original draft, Writing – review & editing. **Keisuke Inomura:** Supervision, Validation, Writing – review & editing. **Xinning Zhang:** Conceptualization, Funding acquisition, Methodology, Resources, Project administration, Supervision, Validation, Visualization, Writing – review & editing.

## Declaration of Competing Interest

The authors declare no competitive interests.

## Appendix A Supplementary material

SI Dataset S1

.

SI R Markdown Code

.

SI Supplementary Material

## References

[1] Bellenger J.-P., Wichard T., Xu Y., Kraepiel A.M.L. (2011). Essential metals for nitrogen fixation in a free-living N_2_-fixing bacterium: chelation, homeostasis and high use efficiency. Environ Microbiol.

[2] Bellenger J.-P., Xu Y., Zhang X., Morel F.M.M., Kraepiel A.M.L. (2014). Possible contribution of alternative nitrogenases to nitrogen fixation by asymbiotic N_2_-fixing bacteria in soils. Soil Biol Biochem.

[3] Bertics V.J., Sohm J.A., Treude T., Chow C.E.T., Capone D.G., Fuhrman J.A. (2010). Burrowing deeper into benthic nitrogen cycling: the impact of bioturbation on nitrogen fixation coupled to sulfate reduction. Mar Ecol Prog Ser.

[4] Capone D.G., Blackburn T.H., Sorensen J. (1988).

[5] Carepo M., Baptista J.F., Pamplona A., Fauque G., Moura J.J.G., Reis M.A.M. (2002). Hydrogen metabolism in Desulfovibrio desulfuricans strain New Jersey (NCIMB 8313)—comparative study with D. vulgaris and D. gigas species. Anaerobe.

[6] Chakraborty S., Andersen K.H., Visser A.W., Inomura K., Follows M.J., Riemann L. (2021). Quantifying nitrogen fixation by heterotrophic bacteria in sinking marine particles. Nat Commun.

[7] Chen M., Chang L., Zhang J., Guo F., Vymazal J., He Q. (2020). Global nitrogen input on wetland ecosystem: the driving mechanism of soil labile carbon and nitrogen on greenhouse gas emissions. Environ Sci Ecotechnol.

[8] Cline J.D. (1969). Spectrophotometric determination of hydrogen sulfide in natural waters. Limnol Oceanogr.

[9] Dahl T.W., Chappaz A., Hoek J., McKenzie C.J., Svane S., Canfield D.E. (2017). Evidence of molybdenum association with particulate organic matter under sulfidic conditions. Geobiology.

[10] Darnajoux R., Bradley R., Bellenger J.P. (2022). In vivo temperature dependency of molybdenum and vanadium nitrogenase activity in the heterocystous cyanobacteria anabaena variabilis. Environ Sci Technol.

[11] Darnajoux R., Reji L., Zhang X.R., Luxem K.E., Zhang X. (2022). Ammonium sensitivity of biological nitrogen fixation by anaerobic diazotrophs in cultures and benthic marine sediments. J Geophys Res: Biogeosci.

[12] Davis L.C., Wang Y.L. (1980). In vivo and in vitro kinetics of nitrogenase. J Bacteriol.

[13] Dekas A.E., Fike D.A., Chadwick G.L., Green-Saxena A., Fortney J., Connon S.A. (2018). Widespread nitrogen fixation in sediments from diverse deep-sea sites of elevated carbon loading. Environ Microbiol.

[14] Deutsch C., Penn J.L., Verberk W.C.E.P., Inomura K., Endress M.G., Payne J.L. (2022). Impact of warming on aquatic body sizes explained by metabolic scaling from microbes to macrofauna. Proc Natl Acad Sci USA.

[15] Dixon R., Kahn D. (2004). Genetic regulation of biological nitrogen fixation. Nat Rev Microbiol.

[16] Droop M.R. (1973). Some thoughts on nutrient limitation in algae. J Phycol.

[17] Du E., Terrer C., Pellegrini A.F.A., Ahlström A., van Lissa C.J., Zhao X. (2020). Global patterns of terrestrial nitrogen and phosphorus limitation. Nat Geosci.

[18] Elser J.J., Bracken M.E.S., Cleland E.E., Gruner D.S., Harpole W.S., Hillebrand H. (2007). Global analysis of nitrogen and phosphorus limitation of primary producers in freshwater, marine and terrestrial ecosystems. Ecol Lett.

[19] Fagerbakke K.M., Heldal M., Norland S. (1996). Content of carbon, nitrogen, oxygen, sulfur and phosphorus in native aquatic and cultured bacteria. Aquat Microb Ecol.

[20] Gallon J.R. (1981). The oxygen sensitivity of nitrogenase: a problem for biochemists and micro-organisms. Trends Biochem Sci.

[21] Gandy E.L., Yoch D.C. (1988). Relationship between nitrogen-fixing sulfate reducers and fermenters in salt marsh sediments and roots of Spar. Appl Environ Microbiol.

[22] Glibert P.M., Wilkerson F.P., Dugdale R.C., Raven J.A., Dupont C.L., Leavitt P.R. (2016). Pluses and minuses of ammonium and nitrate uptake and assimilation by phytoplankton and implications for productivity and community composition, with emphasis on nitrogen-enriched conditions. Limnol Oceanogr.

[23] Großkopf T., LaRoche J. (2012). Direct and indirect costs of dinitrogen fixation in Crocosphaera watsonii WH8501 and possible implications for the nitrogen cycle. Front Microbiol.

[24] Hardy R.W.F., Holsten R.D., Jackson E.K., Burns R.C. (1968). The acetylene - ethylene assay for N_2_ fixation: laboratory and field evaluation. Plant Physiol.

[25] Heidelberg J.F., Seshadri R., Haveman S.A., Hemme C.L., Paulsen I.T., Kolonay J.F. (2004). The genome sequence of the anaerobic, sulfate-reducing bacterium Desulfovibrio vulgaris Hildenborough. Nat Biotechnol.

[26] Helz G.R., Vorlicek T.P. (2019). Precipitation of molybdenum from euxinic waters and the role of organic matter. Chem Geol.

[27] Inomura K., Bragg J., Follows M.J. (2017). A quantitative analysis of the direct and indirect costs of nitrogen fixation: A model based on *Azotobacter vinelandii*. ISME J.

[28] Inomura K., Masuda T., Gauglitz J.M. (2019). Active nitrogen fixation by Crocosphaera expands their niche despite the presence of ammonium – a case study. Sci Rep.

[29] Inomura K., Bragg J., Riemann L., Follows M.J. (2018). A quantitative model of nitrogen fixation in the presence of ammonium. PLoS One.

[30] Inomura K., Deutsch C., Masuda T., Prášil O., Follows M.J. (2020). Quantitative models of nitrogen-fixing organisms. Comput Struct Biotechnol J.

[31] Javelle A., Severi E., Thornton J., Merrick M. (2004). Ammonium sensing in Escherichia coli: Role of the ammonium transporter Amtb and AmtB-GlnK complex formation. J Biol Chem.

[32] Javelle A., Thomas G., Marini A.M., Krämer R., Merrick M. (2005). In vivo functional characterization of the Escherichia coli ammonium channel AmtB: Evidence for metabolic coupling of AmtB to glutamine synthetase. Biochem J.

[33] Kästrier J., Blöchl P.E. (2005). Model for acetylene reduction by nitrogenase derived from density functional theory. Inorg Chem.

[34] Khademi S., O’Connell J., Remis J., Robles-Colmenares Y., Miercke L.J.W., Stroud R.M. (2004). Mechanism of ammonia transport by Amt/MEP/Rh: structure of AmtB at 135 Å. Science.

[35] Kleiner D. (1985). Bacterial ammonium transport. FEMS Microbiol Lett.

[36] Knapp A.N. (2012). The sensitivity of marine N2 fixation to dissolved inorganic nitrogen. Front Microbiol.

[37] LeBauer D.S., Treseder K.K. (2008). Nitrogen limitation of net primary productivity in terrestrial ecosystems is globally distributed. Ecology.

[38] Li Y., Shang J., Zhang C., Zhang W., Niu L., Wang L. (2021). The role of freshwater eutrophication in greenhouse gas emissions: a review. Sci Total Environ.

[39] Masuda T., Inomura K., Takahata N., Shiozaki T., Sano Y., Deutsch C. (2020). Heterogeneous nitrogen fixation rates confer energetic advantage and expanded ecological niche of unicellular diazotroph populations. Commun Biol.

[40] Newell S.E., McCarthy M.J., Gardner W.S., Fulweiler R.W. (2016). Sediment nitrogen fixation: a call for re-evaluating coastal N budgets. Estuaries Coasts.

[41] Newell S.E., Pritchard K.R., Foster S.Q., Fulweiler R.W. (2016). Molecular evidence for sediment nitrogen fixation in a temperate New England estuary. PeerJ.

[42] Noguera D.R., Brusseau G.A., Rittmann B.E., Stahl D.A. (1998). A unified model describing the role of hydrogen in the growth of Desulfovibrio vulgaris under different environmental conditions. *Biotechnol Bioeng*.

[43] Okabe S., Nielsen P.H., Characklis W.G. (1992). Factors affecting microbial sulfate reduction by Desulfovibrio desulfuricans in continuous culture: Limiting nutrients and sulfide concentration. Biotechnol Bioeng.

[44] Oremland R.S., Taylor B.F. (1978). Sulfate reduction and methanogenesis in marine sediments. Geochim Et Cosmochim Acta.

[45] Pahlow M., Dietze H., Oschlies A. (2013). Optimality-based model of phytoplankton growth and diazotrophy. Mar Ecol Prog Ser.

[46] Sabra W., Zeng A.P., Lunsdorf H., Deckwer W.D. (2000). Effect of oxygen on formation and structure of Azotobacter vinelandii alginate and its role in protecting nitrogenase. Appl Environ Microbiol.

[47] Sander R. (2015). Compilation of Henry’s law constants (version 4.0) for water as solvent. Atmos Chem Phys.

[48] Scherer-Lorenzen M., Venterink H.O., Buschmann H. (2008). Nitrogen enrichment and plant invasions: the importance of nitrogen-fixing plants and anthropogenic eutrophication. Biol Invasions.

[49] Schreiber F., Littmann S., Lavik G., Escrig S., Meibom A., Kuypers M.M.M. (2016). Phenotypic heterogeneity driven by nutrient limitation promotes growth in fluctuating environments. Nat Microbiol.

[50] Seefeldt L.C., Hoffman B.M., Dean D.R. (2009). Mechanism of Mo-dependant nitrogenase. Annu Rev Biochem.

[51] Seefeldt L.C., Yang Z.Y., Lukoyanov D.A., Harris D.F., Dean D.R., Raugei S. (2020). Reduction of substrates by nitrogenases. Chem Rev.

[52] Sim M.S., Wang D.T., Zane G.M., Wall J.D., Bosak T., Ono S. (2013). Fractionation of sulfur isotopes by Desulfovibrio vulgaris mutants lacking hydrogenases or type I tetraheme cytochrome c3. Front Microbiol.

[53] Stouthamer A.H., Bettenhaussen C.W. (1977). A continuous culture study of an ATPase-negative mutant of Escherichia coli. Arch Microbiol.

[54] Vorlicek T.P., Helz G.R., Chappaz A., Vue P., Vezina A., Hunter W. (2018). Molybdenum burial mechanism in sulfidic sediments: iron-sulfide pathway. ACS Earth Space Chem.

[55] Voss M., Bange H.W., Dippner J.W., Middelburg J.J., Montoya J.P., Ward B. (2013). The marine nitrogen cycle: Recent discoveries, uncertaintiesand the potential relevance of climate change. Philos Trans R Soc B: Biol Sci.

[56] Vrede K., Heldal M., Norland S., Bratbak G. (2002). Elemental composition (C, N, P) and cell volume of exponentially growing and nutrient-limited bacterioplankton. Appl Environ Microbiol.

[57] Wieder W.R., Cleveland C.C., Smith W.K., Todd-Brown K. (2015). Future productivity and carbon storage limited by terrestrial nutrient availability. *Nat Geosci*.

[58] Williamson G., Tamburrino G., Bizior A., Boeckstaens M., Mirandela G.D., Bage M. (2020). A two-lane mechanism for selective biological ammonium transport. ELife.

[59] Wood J.L., Osman A., Wade S.A. (2019). An efficient, cost-effective method for determining the growth rate of sulfate-reducing bacteria using spectrophotometry. MethodsX.

[60] Zhang X., Ward B.B., Sigman D.M. (2020). Global nitrogen cycle: critical enzymes, organisms, and processes for nitrogen budgets and dynamics. Chem Rev.

[61] Zheng L., Kostrewa D., Bernèche S., Winkler F.K., Li X.D. (2004). The mechanism of ammonia transport based on the crystal structure of AmtB of Escherichia coli. Proc Natl Acad Sci USA.

[62] Zimmermann M., Escrig S., Lavik G., Kuypers M.M.M., Meibom A., Ackermann M. (2018). Substrate and electron donor limitation induce phenotypic heterogeneity in different metabolic activities in a green sulphur bacterium. Environ Microbiol Rep.

