# Supplementary material for: A diazotrophy-ammoniotrophy dual growth model for the sulfate reducing bacterium Desulfovibrio vulgaris var. Hildenborough
Source: Comput Struct Biotechnol J. 2023 May 7;21:3136–48. doi: 10.1016/j.csbj.2023.05.007 (PMC10244686; doi:10.1016/j.csbj.2023.05.007)
Supplement: Supplementary file 2 — SI R Markdown Code [file mmc2.pdf]

Darnajoux_2023_DAD-cellular model_complete

Romain Darnajoux

2023-01-31

R Markdown

This document is a repository of all the code and figure design for the article "A Diazotrophy-Ammoniotrophy Dual Growth Model for the sulfate reducing bacteria Desulfovibrio vulgaris var. Hildenborough" by Darnajoux R., Inomura, K., & Zhang. X., accepted for publication in the journal Computational and Structural Biochemistry Journal

This document regroup all the code for the function required to use the Diazotrophic-Ammoniotrophic Dual Cellular Model applied to the model Sulfate Reducing Bacteria Desulfovibrio vulgaris var. Hildenborough grown under Pyruvate and Lactate as a electron source and the validation data set from batch culture growth experiment.

The final figure from this document were edited from these raw figure using Adobe Illustrator before inclusion in the article.

Communication can be addressed to Romain Darnajoux

(romaind(at)princeton(dot)edu or romain(dot)darnajoux(@)hotmail(dot)fr)

##PACKAGE UPLOAD

```
require(readr)
require(nlstools)
require(nlme)
require(RColorBrewer)
require(scales)
```

##Graphic PARAMETERS

```
col=c(brewer.pal(n = 8, name = "Dark2"), brewer.pal(n = 12, name = "Set3"))
```

##DATA FILES LOADING

```
DATA <- read_delim("20220921_DATA.csv",
                    delim = ";", escape_double = FALSE,
                   col_types = cols(SD_OD_NH4 = col_double(),
                   GR_BNF = col_double()), trim_ws = TRUE)

DATA$EXP=as.factor(DATA$EXP)
# summary(DATA)
```

```r
DATA_GROWTH_1 <- read_delim("2022_DATA_GROWTH.csv",
                            delim = ";", escape_double = FALSE, trim_ws = TRUE)
DATA_GROWTH_1=as.data.frame(DATA_GROWTH_1)

DATA_GROWTH_LIM <- read_delim("2022_DATA_GROWTH_LIM.csv",
                              delim = ";", escape_double = FALSE, trim_ws = TRUE)
DATA_GROWTH_LIM=as.data.frame(DATA_GROWTH_LIM)

DATA_GROWTH_ARA <- read_delim("2022_DATA_GROWTH_ARA.csv",
                              delim = ";", escape_double = FALSE, trim_ws = TRUE)
DATA_GROWTH_ARA=as.data.frame(DATA_GROWTH_ARA)

DATA_GROWTH=list(DATA_GROWTH_LIM, DATA_GROWTH_1, DATA_GROWTH_ARA)

DATA_ADD_DVH <- read_csv("20200506_GROWTH_DvH.csv")

DATA_ADD_DVH$CONDITION=as.factor(DATA_ADD_DVH$CONDITION)

DATA_ADD_DVH_FIG <- read_delim("2023_SRB2019_DATA_FIG1C.csv",
                        delim = ";", escape_double = FALSE, trim_ws = TRUE)
 DATA_ADD_DVH_FIG$CONDITION=as.factor(DATA_ADD_DVH_FIG$CONDITION)
 DATA_ADD_DVH_FIG$CONDITION <- factor(DATA_ADD_DVH_FIG$CONDITION,
        levels=c('CTRL', 'ADD_10', 'ADD_30', 'ADD_100', 'ADD_300', 'ADD_3000'))

# summary(DATA_ADD_DVH)
```

##MODEL CODES##

####FREE PARAMETERS (ARBITRARY DECIDED AND EXPLANATION)

```r
 volume_HS=17
##All experiment where conducted in 27ml Boltch tubes with 10% media.

 KmAC=4
 ###kPa, Slighlty higher than measurement in vivo in cyanobacteria (2.5kPa).
 ### Within literature value (1-8%).
 R_ratio=3.2  ###Theoritical electron ratio per mole of substrate 16e-/5e-,
 ##high uncertainty in the natural environment.

  ##Timing_inhibition= time before ARA start to decrease significantly growth.
 ##Can account for the delay to used up stored N

 ###Timing_switch= Time required to switch from  diazotrophy to ammoniotrophy.
 #Likely reflect the rate of uptake of N until its show significant intracellular increase.

###Lim_nut=
 ##unknown physiological limitation to BNF only, linked in the model to dead biomass.


 ###Adjusted per experiment
 ###Growth rate adjusted for Pyruvate or Lactate
 ###Lim_growth= arbitrary selected to prevent BNF to occurs at OD>0.4.
 ##Twice lower with ARA than natural growth, possibly due to lower QN
 #(low Qn mean more cell per N. So it is likely the lim nut is link
```

```
 ## to metabolic activity or by-product).
```

####DEFAULT METABOLIC AND CELLULAR PARAMETERS OF MODEL ###

```
# metabolism files default
metabolism=data.frame(
  v_BNF=0.041,    ###umol.hr-1.ODml-1 ## Measured on ARA experiment
  cellpermlOD=5.5*10^8 ,  ## LITERATURE
  massOD=0.31, #mg.ODmL-1## LITERATURE
  ODmLperN=0.0003534*1000,  # MEASURED:0.000376
  NperODMl=1/0.0003534/1000,
  mu_NH4=0.105, ## Measured on culture 3 independant experiments
  mu_BNF=0.044, ## Measured on culture 3 independent experiments
  QBNF=0,# Calculated from v measurement
  QNH4=2.8,#2.95, from Noguera et al
  rdeath=0.00243, ## Measured on culture
  Km_NH4=20,##Verified a posteriori
  Ki_NH4=10,# Litterature Darnajoux et al 2022

  BNF_SO4_ratio=21, ##Calculated as QN ~18.9/QN_BNF

  NH4_SO4_ratio=3.31,# measured

  Pyr_SO4_ratio=4,  ##LITERATURE,

  Pyr_ATP=4,  #LItterature assumption, 1 SO4 lead to 1ATP

  Conc_0=7,#7, Initial ammonium-N equivalentin media ~ <10uM

  lim_growth=0.070, #Adjustable parameter

  Timing_switch=0.5, ##Hr, Timing to switch from apparent BNF to NH4-trophy
  #after NH4+ addition, from Darnajoux et al 2022

  delay_inhibition=0 ##Hr,
  #Delay for Acetylene to onset ethylene production, not used
)

metabolism$mu_BNF=metabolism$mu_BNF+metabolism$rdeath
###Adjust apparent growth rate with death rate
metabolism$mu_NH4=metabolism$mu_NH4+metabolism$rdeath
###Adjust apparent growth rate with death rate

metabolism=cbind(metabolism, v_NH4=(metabolism$mu_NH4)*metabolism$QNH4)
##Estimated V_NH4 from data
metabolism$QBNF=metabolism$v_BNF/(metabolism$mu_BNF)
metabolism$BNF_SO4_ratio=18.9/metabolism$QBNF ###
```

###Diazotrophic-Ammoniotrophic Dual Cellular Complete Model (DAD-CM)

```
DvH_cell_add<-function(media=media, metabolism=metabolism){

  ##initial enzymatic parameters
```

```
instant_mu=0
###ARA
ACC=media$acetylene
KmAC=KmAC #2.5#
dEt=0
ppmEt=0
R_ratio=R_ratio
Timinginhibition=media$Timing_inhibition

##BNF
dNfix=0
dOD_BNF=0
lim_nut=media$lim_nut
lim_growth=metabolism$lim_growth
v_BNF_instant=0

Timing_switch_2=media$Timing_switch_2

##NH4

dNH4=0
dOD_NH4=0
v_NH4_instant=0

##ENERGY
dSO4=0
dPyr=0
dSO4_BNF=0
dPyr_BNF=0

##MODEL INITATIATION
time_increment=0.5

##Cell
OD_NH4=media$OD_NH4
OD_BNF=media$OD_BNF
OD_dead=media$OD_dead
OD_cell=media$OD_cell
OD_cell_active=media$OD_cell_active

##Media

conc_SO4_ext=media$conc_SO4_ext
conc_NH4_ext= media$conc_NH4_ext# 500 #
conc_Pyr_ext=media$conc_Pyr_ext
conc_H2S_ext=media$conc_H2S_ext



volume=media$volume
volume_HS=media$volume_HS
```

```
# Ammoniotrophic phase
##(BNF cell can uptake but don't change the total BNF cell
##(BNF capacity is diluted))



if(Timing_switch_2>=metabolism$Timing_switch){

  if(media$conc_NH4_ext<=1 ){
  Timing_switch_2=0
}


v_NH4_instant=metabolism$v_NH4*(1/(1+metabolism$Km_NH4/conc_NH4_ext))
dNH4=(OD_NH4+OD_BNF)*v_NH4_instant*volume*time_increment  #umol N
dOD_NH4=1/metabolism$QNH4/volume*dNH4

instant_mu=v_NH4_instant/metabolism$QNH4


##control ammonium
if(!(((conc_NH4_ext*volume/1000)-dNH4) > 0)) {
## Condition to stop growing if ammonium left is unsufficient
  ##and to use the remaining ammonium.

  dNH4=conc_NH4_ext*volume/1000
  dOD_NH4=dNH4/metabolism$QNH4/volume
}

dSO4=dNH4*metabolism$NH4_SO4_ratio


##control Sulfate
if(!(((conc_SO4_ext*volume/1000)-dSO4) > 0)) {
  ## Condition to stop growing if sulfate left unsufficient
  ##and to use the remaining ammonium.

  dSO4=conc_SO4_ext*volume/1000
  dNH4=metabolism$NH4_SO4_ratio*dSO4
  dOD_NH4=dNH4/metabolism$QNH4/volume

}

dPyr=dSO4*metabolism$Pyr_SO4_ratio

##control pyruvate
if(!(((conc_Pyr_ext*volume/1000)-dPyr) > 0)) {
  ## Condition to stop growing if pyruvate left unsufficient
  ##and to use the remaining ammonium.

  dPyr=conc_Pyr_ext*volume/1000
  dSO4=dPyr/metabolism$Pyr_SO4_ratio
  dNH4=dSO4/metabolism$NH4_SO4_ratio
```

```
  dOD_NH4=dNH4/metabolism$QNH4/volume

}
}
#Diazotrophic phase




if(lim_nut<lim_growth){   ## Condition to use BNF when OD less than 0.4
v_BNF_instant=metabolism$v_BNF*(1/(1+conc_NH4_ext/metabolism$Ki_NH4))

if (Timinginhibition<metabolism$delay_inhibition){
    dNfix=v_BNF_instant*OD_BNF*volume*time_increment  #umol N
    dEt=0

} else {
  dNfix=v_BNF_instant*OD_BNF*volume*time_increment*(1-(ACC/(ACC+KmAC)))

  dEt=v_BNF_instant/2*R_ratio*volume*OD_BNF*time_increment*ACC/(ACC+KmAC)
  ####nmol Et

  }
 ##
  #umol N
dOD_BNF=1/metabolism$QBNF/volume*dNfix #

ppmEt=dEt*8.314*303.15/101500*1000/volume_HS*1000


# dOD_NH4[i]=0
# dNH4[i]=0

dSO4_BNF=dNfix*metabolism$BNF_SO4_ratio/(1-(ACC/(ACC+KmAC)))
###Cost of acetylene taken into account here.


##control Sulfate
if(!(((conc_SO4_ext*volume/1000)-dSO4_BNF) > 0)) {
  ## Condition to stop growing if sulfate left unsufficient
  ## and to use the remaining ammonium.

  dSO4_BNF=conc_SO4_ext*volume/1000
  dNfix=metabolism$BNF_SO4_ratio*dSO4_BNF
  dOD_BNF=dNfix/metabolism$QBNF/volume

}

dPyr_BNF=dSO4_BNF*metabolism$Pyr_SO4_ratio
##control pyruvate
if(!(((conc_Pyr_ext*volume/1000)-dPyr_BNF) > 0)) {
  ## Condition to stop growing if pyruvate left unsufficient
  ## and to use the remaining ammonium.
```

```
    dPyr_BNF=conc_Pyr_ext*volume/1000
    dSO4_BNF=dPyr_BNF/metabolism$Pyr_SO4_ratio
    dNfix=dSO4_BNF/metabolism$BNF_SO4_ratio
    dOD_BNF=dNfix/metabolism$QBNF/volume

  }


}

  #media change
   dOD_dead_BNF=metabolism$rdeath*OD_BNF*time_increment
   dOD_dead_NH4=metabolism$rdeath*OD_NH4*time_increment
   dOD_dead=dOD_dead_BNF+dOD_dead_NH4
   OD_dead=OD_dead+dOD_dead_BNF+dOD_dead_NH4

    OD_NH4=OD_NH4+dOD_NH4-dOD_dead_NH4
    OD_BNF=OD_BNF+dOD_BNF-dOD_dead_BNF


    # OD_BNF=OD_BNF-0.0019*OD_BNF^2-0.0012*OD_BNF
    # OD_NH4=OD_NH4-0.0019*OD_NH4^2-0.0012*OD_NH4

    if(ACC>0){
      Timinginhibition=Timinginhibition+time_increment
    }

    if(conc_NH4_ext>1 ){
      Timing_switch_2=Timing_switch_2+time_increment
    }

   output=data.frame(
     conc_SO4_ext=((conc_SO4_ext*volume/1000)-(dSO4_BNF+dSO4))/volume*1000,
     conc_NH4_ext=((conc_NH4_ext*volume/1000)-dNH4)/volume*1000,
     conc_Pyr_ext=((conc_Pyr_ext*volume/1000)-(dPyr+dPyr_BNF))/volume*1000,
     conc_H2S_ext=((conc_H2S_ext*volume/1000)+(dSO4_BNF+dSO4))/volume*1000,

     volume=media$volume, ### milliliter
     volume_HS=media$volume_HS,
     #cell
     OD_NH4=OD_NH4,
     OD_BNF=OD_BNF,
     OD_dead=OD_dead,
     OD_cell=OD_BNF+OD_NH4+OD_dead,
     OD_cell_active=OD_BNF+OD_NH4
     )

   output=cbind(output,
                cell_active_percent=(OD_BNF+OD_NH4)/OD_cell,
                cell_BNF_percent=OD_BNF/(OD_BNF+OD_NH4),
                v_NH4=v_NH4_instant,
                v_BNF=v_BNF_instant*(1-ACC/(ACC+KmAC)),
                lim_nut=lim_nut+dOD_dead_NH4+dOD_dead_BNF,
```

```
                HS_Et=media$HS_Et+ppmEt,
                acetylene=media$acetylene-(ppmEt/10000),
                Timing_switch_2=Timing_switch_2,
                Timing_inhibition=Timinginhibition)
  return(output)
}
```

###CODE FOR REPRODUCING AMMONIUM ADDITION EXPERIMENT DURING ARA###

```
N_Addition_dvH=function(media=media,
                        metabolism=metabolism,
                        NH4_start=10,
                        EXP_ADD=EXP_ADD,
                        graph=FALSE){

  addition=EXP_ADD$addition
  OD_start=EXP_ADD$OD_start
  acetylene=EXP_ADD$acetylene
  length_EXP=EXP_ADD$EXP_length
  delay=EXP_ADD$delay

    ##MODEL INITATIATION
  i=1
  j=1
  stop=600
  time=0
  time_increment=0.5
  INJ=0
  INJARA=0
  Tzero=10000


  # delay=delay/time_increment
  length_EXP=length_EXP/time_increment

  if (!(is.na(NH4_start))){
    media$conc_NH4_ext=NH4_start
  }

  output=data.frame(Time=as.double(time), media)


  while   ( i < stop) {
    time=time+time_increment
    output=rbind(output,
                 cbind(Time=time, DvH_cell_add(media=output[i,],
                                               metabolism=metabolism)))


    ##START ARA

    if(INJARA==0){
      if((output$OD_cell_active[i]>OD_start)==TRUE){
```

```
        output$acetylene[i+1]=acetylene
        output$HS_Et[i+1]=2*acetylene/10
        INJARA=1
        Tzero=time
        stop=i+length_EXP
      }
    }


    ###Addition of ammonium at selected OD
    if(INJ==0){
   if(((time-Tzero)>delay)==TRUE){
     output$conc_NH4_ext[i+1]=addition
     INJ=1
   }
    }



     i=i+1
  }




  ##Graphic parameters

  if (graph==T){

    par(mfrow=c(3,3))
    #
    #Cell
    plot(OD_NH4~Time,
         data=output,
         xlim=c(Tzero,Tzero+100),
         ylim=c(0, 1.2*max(OD_NH4)),
         main="Biomass_NH4")

    plot(OD_BNF~Time,
         data=output,xlim=c(Tzero,Tzero+100),
         ylim=c(0, 1.2*max(OD_BNF)), main="Biomass_BNF")

    plot(OD_cell_active~Time,
         data=output, xlim=c(Tzero,Tzero+100),
         ylim=c(0, 1.2*max(OD_cell_active)), main="Biomass_Tot")

    plot(v_NH4~Time, data=output,
         xlim=c(Tzero,Tzero+100),ylim=c(0, 1.2*max(v_NH4)),
         main="Growth_rate_NH4")
    plot(v_BNF~Time, data=output,
         xlim=c(Tzero,Tzero+100),ylim=c(0, 1.2*max(v_BNF)),
         main="BNF_rate")
```

```
    #Media
    plot(conc_NH4_ext~Time, data=output,
         xlim=c(Tzero,Tzero+100),ylim=c(0, 1.2*max(conc_NH4_ext)),
         main="conc_NH4_ext")

    plot(conc_H2S_ext~Time, data=output,
         xlim=c(Tzero,Tzero+100),ylim=c(0, 1.2*max(conc_SO4_ext)),
         main="conc_H2S_ext")

    plot(HS_Et~Time, data=output,
         xlim=c(Tzero,Tzero+100),ylim=c(Tzero, 1.2*max(HS_Et)),
         main="conc_Ethylene_HS")

    plot(acetylene~Time, data=output,
         xlim=c(Tzero,Tzero+100), ylim=c(0, 1.2*max(acetylene)),
         main="conc_Acetyelene_HS")

  }


  return(output)





  }
```

###CODE FOR BATCH CULTURE SIMULATION

```
Culture_lim=function(media=media, metabolism=metabolism,
                     NH4=NA, graph=FALSE, method="batch"){

  ##MODEL INITATIATION
  i=1
  j=1
  stop=600
  time=0
  time_increment=0.5


   if (!(is.na(NH4))){
    media$conc_NH4_ext=NH4
   }

  if(media$OD_NH4==0){
    media$OD_NH4=media$OD_NH4+0.0000001

  }



  if(method=="batch"){
```

```
  output=data.frame(Time=as.double(time), media)

while   ( i < stop ) {
  time=time+time_increment
output=rbind(output, cbind(Time=time,
                           DvH_cell_lim(output[i,],
                                        metabolism=metabolism)))
 i=i+1
}
}


if (method=="addition"){

  output=data.frame(Time=as.double(time), media)

  while   ( i < stop) {
    time=time+time_increment
    output=rbind(output, cbind(Time=time,
                           DvH_cell_add(output[i,],
                                        metabolism=metabolism)))
    i=i+1
  }


}

##Graphic parameters

if (graph==T){


  #Cell
  plot(OD_NH4~Time, data=output,
       ylim=c(0, 1.2*max(OD_NH4)), main="Biomass_NH4")

  plot(OD_BNF~Time, data=output,
       ylim=c(0, 1.2*max(OD_BNF)), main="Biomass_BNF")

  plot(OD_cell_active~Time, data=output,
       ylim=c(0, 1.2*max(OD_cell_active)), main="Biomass_Tot")

  plot(v_NH4~Time, data=output,
       ylim=c(0, 1.2*max(v_NH4)), main="Growth_rate_NH4")

  plot(v_BNF~Time, data=output,
       ylim=c(0, 1.2*max(v_BNF)), main="BNF_rate")


  #Media
  plot(conc_NH4_ext~Time, data=output,
       ylim=c(0, 1.2*max(conc_NH4_ext)), main="conc_NH4_ext")
```

```
    plot(conc_Pyr_ext~Time, data=output,
         ylim=c(0, 1.2*max(conc_Pyr_ext)), main="conc_Pyr_ext")

    plot(conc_SO4_ext~Time, data=output,
         ylim=c(0, 1.2*max(conc_SO4_ext)), main="conc_SO4_ext")

    plot(conc_H2S_ext~Time, data=output,
         ylim=c(0, 1.2*max(conc_SO4_ext)), main="conc_H2S_ext")

  }

  return(output)
  }
```

###METHOD TO ACQUIRE GROWTH RATE FROM OUTPUT FILES

```
#####(BASED ON SECONDARY DERIVATIVES OF PURE BNF/NH4 ONLY CELL BIOMASS)

growthrate=function(data=data, graph=FALSE){


result=data


attach(result, warn.conflicts = F)
tim_NH4=0
tim=0
tim_BNF=0
### Ammoniotrophic growth
  #(left to separate the exact timing using the pure NH4 and BNF growth,
#then apply timing to the total OD) break as the stop.

dODdT=0
t=result$Time
###Function to extract slope from simulation data
OD_tot=result$OD_NH4


###identified breaks in plot using derivative

for (i in 1:length(t)){
    dODdT[i]=(OD_tot[i+1]-OD_tot[i])/(t[i+1]-t[i])

}
       # plot(dODdT~t)

dODdT2=0
for (i in 1:length(t)){
  dODdT2[i]=(dODdT[i+1]-dODdT[i])/(t[i+1]-t[i])

}
      # plot(dODdT2~t)
```

```
if(graph==TRUE){
  plot(log(OD_cell_active)~Time, data=result,
       main=paste(a,result$conc_NH4_ext[1],""))
}



if(!(result$conc_NH4_ext[1]<11)){



tim_NH4=t[na.exclude(dODdT2==min(dODdT2[(dODdT2<0)&!is.na(dODdT2)]))]
tim=mean(tim_NH4)


lmNH4_true=lm(log(OD_NH4)~Time,
              data=result[result$Time<min(tim) & result$Time >5,])

  lmNH4_app=lm(log(OD_cell_active)~Time,
               data=result[result$Time<min(tim) & result$Time>5,])

  if(graph==TRUE){
    lines(result$Time[result$Time<min(tim) & result$Time > 5],
          predict(lm(log(OD_cell_active)~Time,
                     data=result[result$Time<min(tim) & result$Time>5,])),
          col="red")
  }

  mu_NH4_true=as.double(lmNH4_true$coefficients[2])

  mu_NH4_app=as.double(lmNH4_app$coefficients[2])

} else {

  lmNH4_true=lm(log(OD_NH4)~Time,
                data=result[result$Time<30 & result$Time>0,])
  mu_NH4_true=as.double(lmNH4_true$coefficients[2])

  lmNH4_app=lm(log(OD_cell_active)~Time,
               data=result[result$Time<10 & result$Time>0,])
  mu_NH4_app=as.double(lmNH4_app$coefficients[2])

   }

dODdT=0
###Function to extract slope from simulation data for BNF only
OD_tot=result$OD_BNF


###identified breaks in plot using derivative

for (i in 1:length(t)){
  dODdT[i]=(OD_tot[i+1]-OD_tot[i])/(t[i+1]-t[i])
```

```
}
 # plot(dODdT~t)

dODdT2=0
for (i in 1:length(t)){
  dODdT2[i]=(dODdT[i+1]-dODdT[i])/(t[i+1]-t[i])

}
  # plot(dODdT2~t)

tim_BNF=t[(dODdT2<0)&!is.na(dODdT2)]
tim=tim_BNF

window_reg=100
 if(result$conc_NH4_ext[1]<1500) {
  lmBNF_true=lm(log(OD_BNF)~Time,
  data=result[result$Time < min(tim_BNF) & result$Time > min (tim_BNF)-50 ,])

  lmBNF_app=lm(log(OD_cell_active)~Time,
  data=result[result$Time < min(tim_BNF) & result$Time > min (tim_BNF)-50 ,])

  if(graph==TRUE){
    lines(result$Time[result$Time < min(tim_BNF) & result$Time > min (tim_BNF)-50],
    predict(lm(log(OD_cell_active)~Time,
    data=result[result$Time < min(tim_BNF) & result$Time > min (tim_BNF)-50,])),
    col="red")
  }

} else {
  lmBNF_true=lm(log(OD_BNF)~Time,
              data=result[result$Time> max (tim_NH4) ,])
  lmBNF_app=lm(log(OD_cell_active)~Time,
             data=result[result$Time> max (tim_NH4) ,])

  if(graph==TRUE){
   lines(result$Time[result$Time> max (tim_NH4)],
         predict(lm(log(OD_cell_active)~Time,
                data=result[result$Time> max (tim_NH4),])),
         col="red")
  }

}


  #### Table construction

mu_BNF_app=as.double(lmBNF_app$coefficients[2])
mu_BNF_true=as.double(lmBNF_true$coefficients[2])

result3=data.frame(mu_NH4_true,
                   mu_BNF_true,
                   mu_NH4_app,
                   mu_BNF_app)
```

```
return(result3)


}
```

##MODEL SIMULATION AND VALIDATION##

###EXPERIENCE LACTATE ARA ADDITION ####MEDIA AND HEASPACE COMPOSITON INTIALIZATION

```
####Media
constant=0#0.00753

media=data.frame(
  conc_SO4_ext=20000,
  conc_NH4_ext=0,
  conc_Pyr_ext=40000,
  conc_H2S_ext=0,


  volume=10,
  volume_HS=volume_HS,### milliliter

  #work fine cell   alive= 0.1 BNF= 0.8
  OD_NH4=0.000,###0.0005

  OD_BNF=0.002, ###0.005

  OD_cell=0.002 ####0.005


)

media=cbind(media[,1:8],
            OD_dead=media$OD_cell-media$OD_NH4-media$OD_BNF,
            OD_cell=media[,9],
            OD_cell_active=(media$OD_NH4+media$OD_BNF))

media=cbind(media[,1:11],
            cell_active_percent=media$OD_cell_active/media$OD_cell,
            cell_BNF_percent=media$OD_BNF/media$OD_cell_active,
            v_NH4=0, v_BNF=0, lim_nut=0, HS_Et=0, acetylene=0,
            Timing_switch_2=0, Timing_inhibition=0)
```

####METABOLIC AND CELLULAR PARAMETERS SPECIFIC MODIFICATION

```
 metabolism$mu_NH4=0.045
  metabolism$mu_BNF=0.022
metabolism$Pyr_SO4_ratio=2
    metabolism$Pyr_ATP=2
metabolism$lim_growth=0.02
```

```
metabolism$mu_BNF=metabolism$mu_BNF+metabolism$rdeath
metabolism$mu_NH4=metabolism$mu_NH4+metabolism$rdeath

metabolism$v_NH4=(metabolism$mu_NH4+constant)*metabolism$QNH4
metabolism$v_BNF=(metabolism$mu_BNF+constant)*metabolism$QBNF
```

####CODE FOR LACTACTE EXPERIMENT FIGURE COMPARISON

```
# par(mfrow=c(4,3))

conc=c(10,400, 800)
ODstart=c(0.085,0.08,0.08, 0.085)

####UNCERTAINTY
conc=c(10,10,10,400,400,400,800,800,800)
ODstart=c(0.075,0.085,0.095,0.075,
          0.085,0.095,0.075,0.085,
          0.095,0.075,0.085,0.095)

timing_sampling=c(seq(0,100, 1))


dat=list(data.frame())
tab=list(data.frame())
a="growth_"
b="Addition_"

EXP_ADD=data.frame(addition=0,
                   OD_start=0.085,
                   acetylene=1.7,
                   delay=15, EXP_length=100)

for (i in 1:length(conc)){
  EXP_ADD$addition=conc[i]
  EXP_ADD$OD_start=ODstart[i]
  dat[[i]]=N_Addition_dvH(media=media,
                          metabolism=metabolism,
                          NH4_start=0,
                          EXP_ADD=EXP_ADD, graph=FALSE)


  names(dat)[i]=paste(a,conc[i],"")
  start=as.double(min(which(dat[[i]]$acetylene > 0)))



tab1=data.frame(Time=as.double(0),
                HS_Et=as.double(dat[[i]]$HS_Et[start]),
                OD=as.double(dat[[i]]$OD_cell_active[start]),
                NH4=as.double(dat[[i]]$conc_NH4_ext[start]),
                Condition=as.factor(paste(b,conc[i],"")))

   for (j in 2:length(timing_sampling)){
```

```
  stop=start+timing_sampling[j]/0.5
  tab1=rbind(tab1,
             cbind.data.frame(Time=timing_sampling[j],
            HS_Et=dat[[i]]$HS_Et[stop],
             OD=as.double(dat[[i]]$OD_cell_active[stop]),
             NH4=as.double(dat[[i]]$conc_NH4_ext[stop]),
               Condition=paste(b,conc[i],"") ))
    }

tab[[i]]=tab1
names(tab)[i]=paste(b,conc[i],"")


}

 # pdf("2023_Figure5B.pdf", height = 11, width=7)

par(mfrow=c(3,1))
COMPARE=TRUE
BAND=F
#######PANEL A


###GROWTH
if(BAND==F){
plot(HS_Et~Time, pch=16, data=tab[[1]],
     cex=3, col=col[1],lwd=2,
     xlim=c(0,92),type="l", ylim=c(0,700),
     cex.lab=2, cex.axis=2)

for (i in 2: length(tab)){
  lines(HS_Et~Time, data=tab[[i]],
        pch=16,cex=2, lwd=2, col=col[i])

}

}


if(BAND==T){
######UNCERTAINTY BAND

plot(HS_Et~Time, data=tab[[2]],
     col=col[1], xlim=c(0,92),type="l",
     ylim=c(0,700), lwd=2, cex=3,
     xlab="Time since ARA start)",
     ylab="Headspace_Ethylene (ppm)",
     cex.lab=2, cex.axis=2)

polygon(c(tab[[2]]$Time,rev(tab[[2]]$Time)),
        c(tab[[1]]$HS_Et,rev(tab[[3]]$HS_Et)),
        col=alpha(col[1], alpha=0.2),border=NA)
```

```
for (i in 2:3){
    lines(HS_Et~Time, data=tab[[i*3-1]],
          col=col[i], xlim=c(0,92),type="l",
          ylim=c(0,700), lwd=2, cex=2,
          xlab="Time since ARA start)",
          ylab="Headspace_Ethylene (ppm)",
          cex.lab=2, cex.axis=2)

  polygon(c(tab[[i*3-1]]$Time,
            rev(tab[[i*3-1]]$Time)),
          c(tab[[i*3-2]]$HS_Et,rev(tab[[i*3]]$HS_Et)),
          col=alpha(col[i], alpha=0.2),border=NA)

}

}

if(COMPARE==TRUE){
  points(ETHYLENE~TIME_EXP, data=DATA_ADD_DVH,
         pch=21, col=col[DATA_ADD_DVH$CONDITION], cex=3)

arrows(DATA_ADD_DVH$TIME_EXP,
       DATA_ADD_DVH$ETHYLENE-DATA_ADD_DVH$SDEt,
       DATA_ADD_DVH$TIME_EXP,
       DATA_ADD_DVH$ETHYLENE+DATA_ADD_DVH$SDEt,
       code=0,col=col[DATA_ADD_DVH$CONDITION],lwd = 2 )


}



###########PANEL B
###No ARA Growth control

dat0=N_Addition_dvH(media=media,
                    metabolism=metabolism,
                    NH4_start=0,
                    EXP_ADD=data.frame(addition=0,
                                       OD_start=0.075,
                                       acetylene=0, delay=15,
                                       EXP_length=100) ,
                    graph=FALSE)

start=as.double(min(which(dat0$OD_cell_active > ODstart[10])))
tab0=data.frame(Time=as.double(0),
                HS_Et=as.double(dat0$HS_Et[start]),
                OD=as.double(dat0$OD_cell_active[start]),
                NH4=as.double(dat0$conc_NH4_ext[start]),
                Condition=as.factor("No AC"))
```

```
for (j in 2:length(timing_sampling)){

  stop=start+timing_sampling[j]/0.5
  tab0=rbind(tab0,
             cbind.data.frame(Time=timing_sampling[j],
                      HS_Et=dat0$HS_Et[stop],
                      OD=as.double(dat0$OD_cell_active[stop]),
                      NH4=as.double(dat0$conc_NH4_ext[stop]),
                      Condition="No AC" ))
}

ctrl_low=tab0

dat0=N_Addition_dvH(media=media,
                    metabolism=metabolism,
                    NH4_start=0,
                    EXP_ADD=data.frame(addition=0,
                                       OD_start=0.085,
                                       acetylene=0,
                                       delay=15,
                                       EXP_length=100) ,
                    graph=FALSE)

start=as.double(min(which(dat0$OD_cell_active > ODstart[11])))
tab0=data.frame(Time=as.double(0),
                HS_Et=as.double(dat0$HS_Et[start]),
                OD=as.double(dat0$OD_cell_active[start]),
                NH4=as.double(dat0$conc_NH4_ext[start]),
                Condition=as.factor("No AC"))


for (j in 2:length(timing_sampling)){

  stop=start+timing_sampling[j]/0.5
  tab0=rbind(tab0,
             cbind.data.frame(Time=timing_sampling[j],
               HS_Et=dat0$HS_Et[stop],
               OD=as.double(dat0$OD_cell_active[stop]),
               NH4=as.double(dat0$conc_NH4_ext[stop]),
              Condition="No AC" ))
}
ctrl_mean=tab0

dat0=N_Addition_dvH(media=media,
                    metabolism=metabolism,
                    NH4_start=0,
                    EXP_ADD=data.frame(addition=0,
                                       OD_start=0.095,
                                       acetylene=0,
                                       delay=15,
                                       EXP_length=100) ,
                    graph=FALSE)
```

```
start=as.double(min(which(dat0$OD_cell_active > ODstart[12])))
tab0=data.frame(Time=as.double(0),
                HS_Et=as.double(dat0$HS_Et[start]),
                OD=as.double(dat0$OD_cell_active[start]),
                NH4=as.double(dat0$conc_NH4_ext[start]),
                Condition=as.factor("No AC"))

for (j in 2:length(timing_sampling)){

  stop=start+timing_sampling[j]/0.5
  tab0=rbind(tab0,cbind.data.frame(Time=timing_sampling[j],
                    HS_Et=dat0$HS_Et[stop],
                    OD=as.double(dat0$OD_cell_active[stop]),
                    NH4=as.double(dat0$conc_NH4_ext[stop]),
                    Condition="No AC" ))
}

ctrl_high=tab0


if(BAND==F){
    plot(OD~Time, pch=16,
         data=tab[[1]], cex=3,
         col=col[1],type="l", lwd=2,
         xlim=c(0,92), ylim=c(0,0.4),
         cex.lab=2, cex.axis=2)

for (i in 2: length(tab)){
  lines(OD~Time, data=tab[[i]],
        pch=16,cex=2, col=col[i],
        lwd=2, xlim=c(0,92), ylim=c(0,0.4))

}
 lines(OD~Time, data=ctrl_high,
       cex=2, xlim=c(0,285), ylim=c(0,1),
       pch=16, lwd=2, col=col[4],
       xlim=c(0,92), ylim=c(0,0.4))

  lines(OD~Time, data=ctrl_mean,
        cex=2, xlim=c(0,285), ylim=c(0,1),
        pch=16, lwd=2, col=col[4], xlim=c(0,92),
        ylim=c(0,0.4))

   lines(OD~Time, data=ctrl_low,
         cex=2, xlim=c(0,285), ylim=c(0,1),
         pch=16, lwd=2, col=col[4],
         xlim=c(0,92), ylim=c(0,0.4))

 if(COMPARE==TRUE){
 points(OD600~TIME_EXP,
        data=DATA_ADD_DVH, pch=21,
        col=col[DATA_ADD_DVH$CONDITION],
        cex=3)
```

```
  arrows(DATA_ADD_DVH$TIME_EXP,
         DATA_ADD_DVH$OD600-DATA_ADD_DVH$SD,
         DATA_ADD_DVH$TIME_EXP,DATA_ADD_DVH$OD600+DATA_ADD_DVH$SD,
         code=0,col=col[DATA_ADD_DVH$CONDITION],lwd = 2 )
  }

}

if(BAND==T){
  ######UNCERTAINTY BAND
  # attach(tab[[1]], warn.conflicts = F)
  plot(OD~Time, pch=16, data=tab[[2]],
       cex=3, col=col[1],type="l",
       lwd=2, xlim=c(0,92), ylim=c(0,0.4),
       xlab="Time since ARA start)",
       ylab="OD600", cex.lab=2, cex.axis=2)
  polygon(c(tab[[2]]$Time,rev(tab[[2]]$Time)),
          c(tab[[1]]$OD,rev(tab[[3]]$OD)),
          col=alpha(col[1], alpha=0.2),border=NA)



  for (i in 2:3){
    lines(OD~Time, data=tab[[i*3-1]],
          col=col[i],xlim=c(0,92),
          ylim=c(0,0.4),type="l",
          lwd=2, cex=2, xlab="Time since ARA start)",
          ylab="OD600", cex.lab=2, cex.axis=2)
    polygon(c(tab[[i*3-1]]$Time,rev(tab[[i*3-1]]$Time)),
            c(tab[[i*3-2]]$OD,rev(tab[[i*3]]$OD)),
            col=alpha(col[i], alpha=0.2),border=NA)

  }

  ###Ctrl No ARA
  lines(OD~Time, data=ctrl_mean,
        col=col[5],xlim=c(0,92),
        ylim=c(0,0.4),type="l",
        lwd=2, cex=2, xlab="Time since ARA start)",
        ylab="OD600", cex.lab=2, cex.axis=2)
  polygon(c(ctrl_mean$Time,rev(ctrl_mean$Time)),
          c(ctrl_low$OD,rev(ctrl_high$OD)),
          col=alpha(col[5], alpha=0.2),border=NA)
}


  if(COMPARE==TRUE){
    points(OD600~TIME_EXP,
           data=DATA_ADD_DVH, pch=21,
           col=col[c(1:3,5)][DATA_ADD_DVH$CONDITION],
           cex=3)
    arrows(DATA_ADD_DVH$TIME_EXP,
           DATA_ADD_DVH$OD600-DATA_ADD_DVH$SD,
```

```
          DATA_ADD_DVH$TIME_EXP,
          DATA_ADD_DVH$OD600+DATA_ADD_DVH$SD,
          code=0,col=col[c(1:3,5)][DATA_ADD_DVH$CONDITION],
          lwd = 2 )
 }


 ######PANEL C

if(BAND==F){

plot(NH4~Time, data=tab[[length(tab)]],
     pch=16, lwd=2, col=col[length(tab)], type="l",
     cex=3, xlim=c(0,92), ylim=c(0,1000), cex.lab=2,
     cex.axis=2)

for (i in 1: (length(tab)-1)){
  lines(NH4~Time, data=tab[[i]],
        pch=16,cex=2, lwd=2, col=col[i],
        xlim=c(0,92), ylim=c(0,1000))

}

if(COMPARE==TRUE){
points(NH4~TIME_EXP,
       data=DATA_ADD_DVH,
       pch=21, col=col[DATA_ADD_DVH$CONDITION],
       cex=3)
arrows(DATA_ADD_DVH$TIME_EXP,
       DATA_ADD_DVH$NH4-DATA_ADD_DVH$SDN,
       DATA_ADD_DVH$TIME_EXP,DATA_ADD_DVH$NH4+DATA_ADD_DVH$SDN,
       code=0,col=col[DATA_ADD_DVH$CONDITION],lwd = 2 )
}

legend(60,1000,names(tab), pch=16, col=col)
}
```

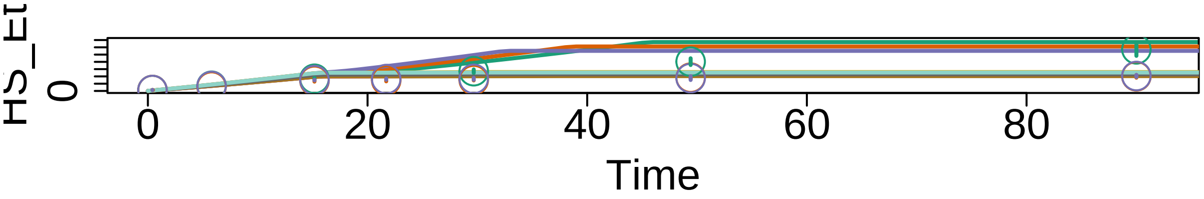

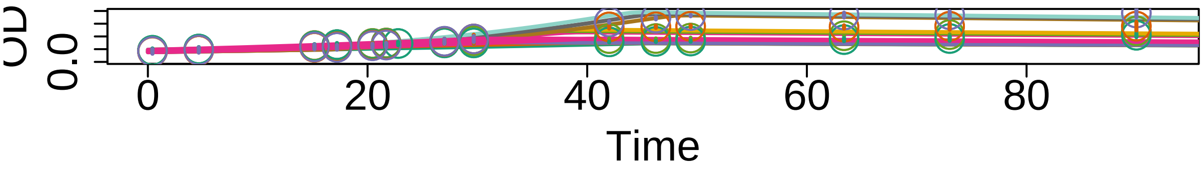

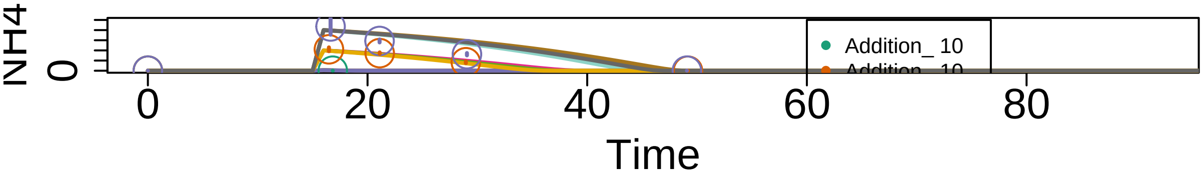


```
if(BAND==T){
######UNCERTAINTY BAND

plot(NH4~Time, data=tab[[2]],
     col=col[1], xlim=c(0,92), ylim=c(0,1000),
     type="l", lwd=2, cex=3, xlab="Time since ARA start)",
     ylab="[NH4+] in media (uM)", cex.lab=2, cex.axis=2)
polygon(c(tab[[2]]$Time,rev(tab[[2]]$Time)),
        c(tab[[1]]$NH4,rev(tab[[3]]$NH4)),
        col=alpha(col[1], alpha=0.2),border=NA)



for (i in 2:3){
  lines(NH4~Time, data=tab[[i*3-1]],
        col=col[i], type="l", xlim=c(0,92),
        ylim=c(0,1000), lwd=2, cex=2,
        xlab="Time since ARA start)", ylab="OD600",
        cex.lab=2, cex.axis=2)

  polygon(c(tab[[i*3-1]]$Time,rev(tab[[i*3-1]]$Time)),
          c(tab[[i*3-2]]$NH4,rev(tab[[i*3]]$NH4)),
          col=alpha(col[i], alpha=0.2),border=NA)

}
```

```r
if(COMPARE==TRUE){
  points(NH4~TIME_EXP, data=DATA_ADD_DVH,
         pch=21, col=col[DATA_ADD_DVH$CONDITION], cex=3)
  arrows(DATA_ADD_DVH$TIME_EXP,
         DATA_ADD_DVH$NH4-DATA_ADD_DVH$SDN,
         DATA_ADD_DVH$TIME_EXP,
         DATA_ADD_DVH$NH4+DATA_ADD_DVH$SDN,
         code=0,col=col[DATA_ADD_DVH$CONDITION],lwd = 2 )
}

legend(60,1000,c("No add", "+400uM", "+800uM"),
       pch=16, col=col[1:3], cex=2)
}

 # dev.off()
```

####FITTING ESTIMATE

```r
###MESSQ for DATA LAC addition


###Ethylene

MESQ_LAC=data.frame(NH4=as.double(),exp=as.double(),
                    theo=as.double())


for(k in 1:(length(attributes(DATA_ADD_DVH$CONDITION)$levels)-1)){

   data_lac=DATA_ADD_DVH[which(DATA_ADD_DVH$CONDITION
            ==attributes(DATA_ADD_DVH$CONDITION)$levels[k]),]

for (i in 1:nrow(data_lac)){

  MESQ_LAC_x=data.frame(NH4=as.double(),
                        exp=as.double(),
                        theo=as.double())

  if(!(is.na(data_lac$ETHYLENE[i]))){

  MESQ_LAC_x[1,1]=data_lac$TIME_EXP[i]
  MESQ_LAC_x[1,2]=tab[[k*3-1]]$HS_Et[which(
    round(tab[[k*3-1]]$Time)
    ==round(data_lac$TIME_EXP[i]))]
  MESQ_LAC_x[1,3]=data_lac$ETHYLENE[i]

 MESQ_LAC=rbind.data.frame(MESQ_LAC,MESQ_LAC_x)
 MESQ_LAC_x=0

  }

  }
}
```

```
plot(MESQ_LAC$exp~MESQ_LAC$theo,
     main="Predict vs Measured Maximal Biomass Yield",
     xlab="Measured values", ylab="Modeled values")


abline(0,1, col="red")
```

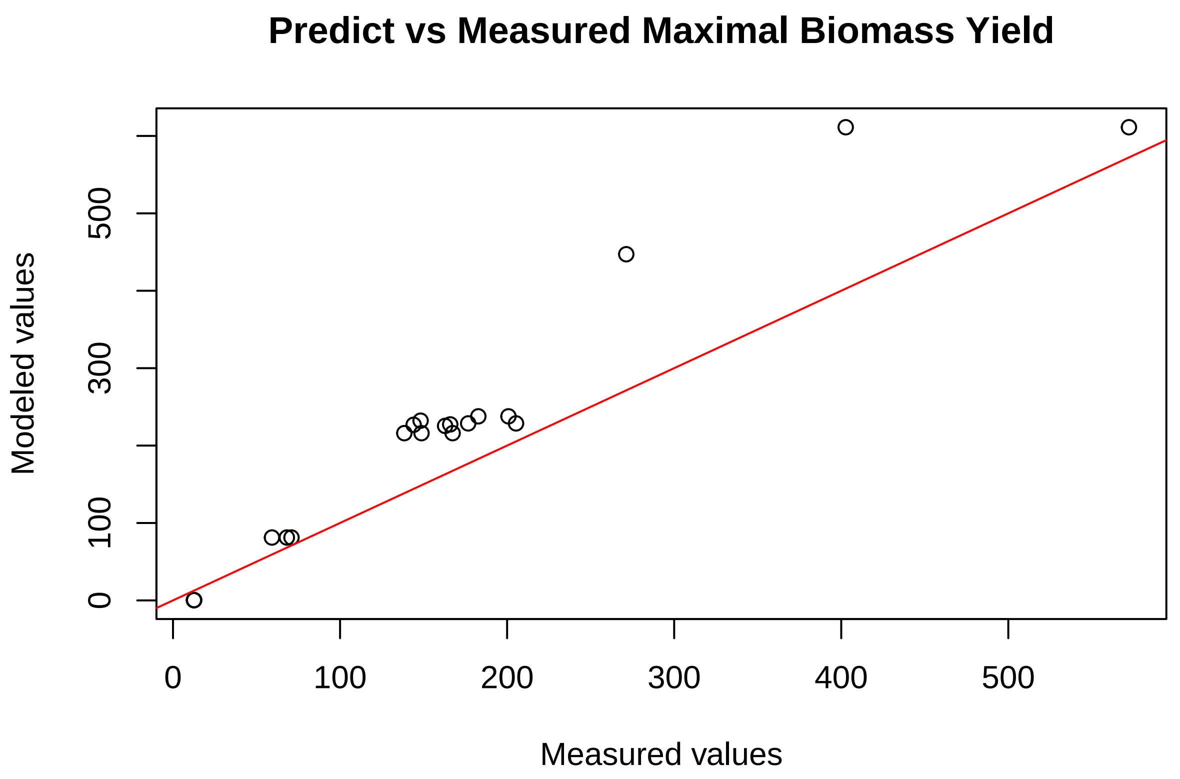


```
print("ETHYLENE")
```

```
## [1] "ETHYLENE"
```

```
##Is the regrssion good? Adj-R2=
summary(lm((MESQ_LAC$exp)~MESQ_LAC$theo))
```

```
##
## Call:
## lm(formula = (MESQ_LAC$exp) ~ MESQ_LAC$theo)
##
## Residuals:
##      Min       1Q   Median       3Q      Max
## -113.161  -26.472   -5.286   20.530   97.289
##
## Coefficients:
```

```
##                  Estimate Std. Error t value Pr(>|t|)
## (Intercept)     14.07307   17.30897   0.813    0.427
## MESQ_LAC$theo    1.24134    0.08205  15.129 1.12e-11 ***
## ---
## Signif. codes:  0 '***' 0.001 '**' 0.01 '*' 0.05 '.' 0.1 ' ' 1
##
## Residual standard error: 47.68 on 18 degrees of freedom
## Multiple R-squared:  0.9271, Adjusted R-squared:  0.923
## F-statistic: 228.9 on 1 and 18 DF,  p-value: 1.116e-11
```

```
##Is prediction different from measured (is measured~prediction a 1:1 line)
##p value>0.05 and slope value if not.
plot((MESQ_LAC$exp-MESQ_LAC$theo)~MESQ_LAC$theo)
```

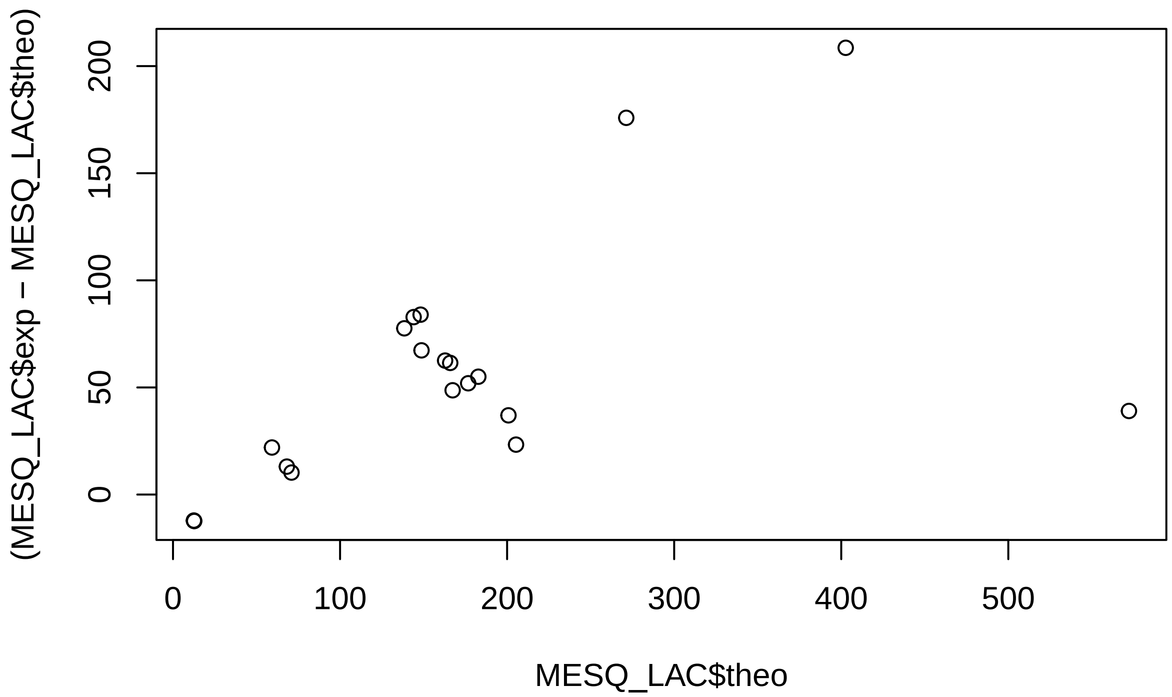


```
summary(lm((MESQ_LAC$exp-MESQ_LAC$theo)~MESQ_LAC$theo))
```

```
##
## Call:
## lm(formula = (MESQ_LAC$exp - MESQ_LAC$theo) ~ MESQ_LAC$theo)
##
## Residuals:
##      Min       1Q   Median       3Q      Max
## -113.161  -26.472   -5.286   20.530   97.289
##
## Coefficients:
```

```
##                Estimate Std. Error t value Pr(>|t|)
## (Intercept)    14.07307   17.30897   0.813  0.42681
## MESQ_LAC$theo  0.24134    0.08205   2.941  0.00873 **
## ---
## Signif. codes:  0 '***' 0.001 '**' 0.01 '*' 0.05 '.' 0.1 ' ' 1
##
## Residual standard error: 47.68 on 18 degrees of freedom
## Multiple R-squared:  0.3246, Adjusted R-squared:  0.2871
## F-statistic: 8.652 on 1 and 18 DF,  p-value: 0.008726
```

```
###OD_LAC

MESQ_LAC=data.frame(NH4=as.double(),exp=as.double(),
                    theo=as.double())

# k=1
# i=1

for(k in 1:(length(attributes(DATA_ADD_DVH$CONDITION)$levels)-1)){

   data_lac=DATA_ADD_DVH[which(DATA_ADD_DVH$CONDITION
        ==attributes(DATA_ADD_DVH$CONDITION)$levels[k]),]

for (i in 1:nrow(data_lac)){

  MESQ_LAC_x=data.frame(NH4=as.double(),exp=as.double(),
                        theo=as.double())

  if(!(is.na(data_lac$OD600[i]))){
    if(!(is.na(data_lac$TIME_EXP[i]))){

  MESQ_LAC_x[1,1]=data_lac$TIME_EXP[i]
  MESQ_LAC_x[1,2]=tab[[k*3-1]]$OD[which(round(tab[[k*3-1]]$Time)
                          ==round(data_lac$TIME_EXP[i]))]
  MESQ_LAC_x[1,3]=data_lac$OD600[i]

 MESQ_LAC=rbind.data.frame(MESQ_LAC,MESQ_LAC_x)
 MESQ_LAC_x=0

    }
  }

  }
}

data_lac=DATA_ADD_DVH[which(DATA_ADD_DVH$CONDITION
              ==attributes(DATA_ADD_DVH$CONDITION)$levels[4]),]

for (i in 1:nrow(data_lac)){

  MESQ_LAC_x=data.frame(NH4=as.double(),
                        exp=as.double(),
                        theo=as.double())
```

```
  if(!(is.na(data_lac$OD600[i]))){
    if(!(is.na(data_lac$TIME_EXP[i]))){

  MESQ_LAC_x[1,1]=data_lac$TIME_EXP[i]
  MESQ_LAC_x[1,2]=ctrl_mean$OD[which(round(ctrl_mean$Time)
                      ==round(data_lac$TIME_EXP[i]))]
  MESQ_LAC_x[1,3]=data_lac$OD600[i]

 MESQ_LAC=rbind.data.frame(MESQ_LAC,MESQ_LAC_x)
 MESQ_LAC_x=0

    }
  }

  }


plot(MESQ_LAC$exp~MESQ_LAC$theo,
     main="Predict vs Measured Maximal Biomass Yield",
     xlab="Measured values", ylab="Modeled values")


abline(0,1, col="red")
```

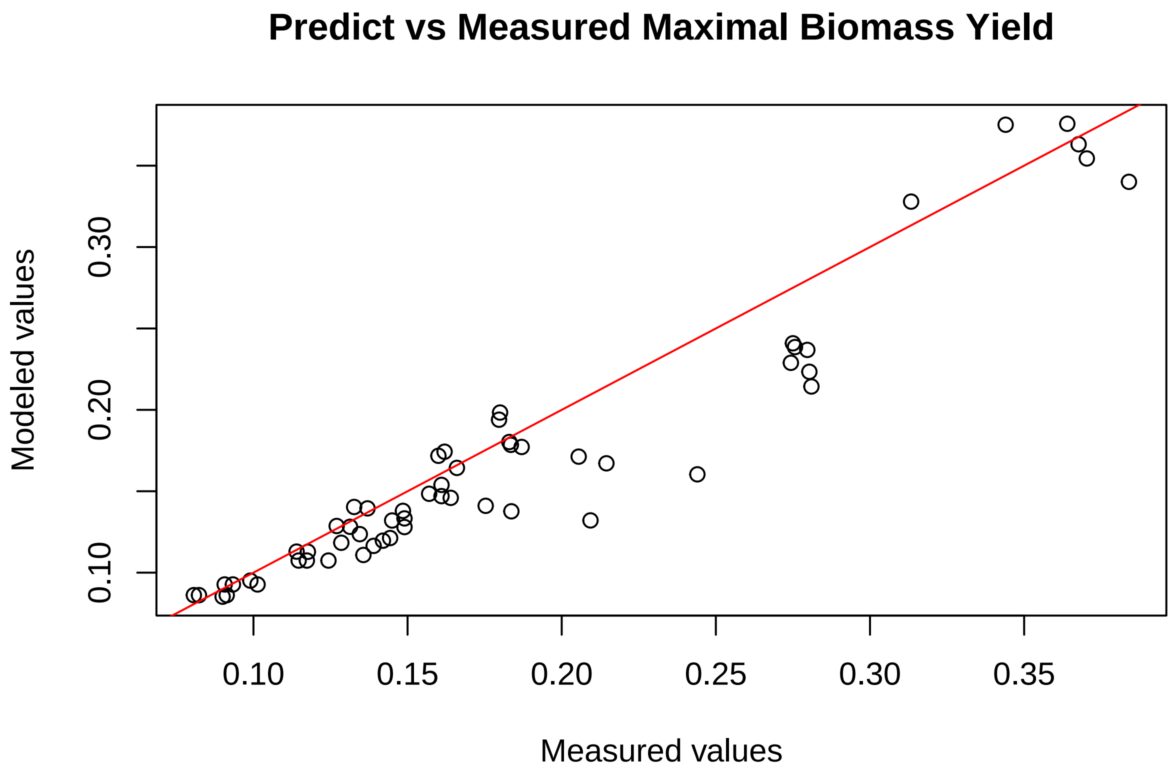

```r
print("BIOMASS_LACTATE")
```

```
## [1] "BIOMASS_LACTATE"
```

```r
##Is the regrssion good? Adj-R2=
summary(lm((MESQ_LAC$exp)~MESQ_LAC$theo))
```

```
##
## Call:
## lm(formula = (MESQ_LAC$exp) ~ MESQ_LAC$theo)
##
## Residuals:
##       Min        1Q    Median        3Q       Max
## -0.063668 -0.011440  0.001925  0.011781  0.059411
##
## Coefficients:
##               Estimate Std. Error t value Pr(>|t|)
## (Intercept)   0.0003958  0.0074120   0.053    0.958
## MESQ_LAC$theo 0.9166503  0.0373593  24.536   <2e-16 ***
## ---
## Signif. codes:  0 '***' 0.001 '**' 0.01 '*' 0.05 '.' 0.1 ' ' 1
##
## Residual standard error: 0.02259 on 55 degrees of freedom
## Multiple R-squared:  0.9163, Adjusted R-squared:  0.9148
## F-statistic:   602 on 1 and 55 DF,  p-value: < 2.2e-16
```

```r
##Is prediction different from measured (is measured~prediction a 1:1 line)
##, p value>0.05 and slope value if not.
plot((MESQ_LAC$exp-MESQ_LAC$theo)~MESQ_LAC$theo)
```

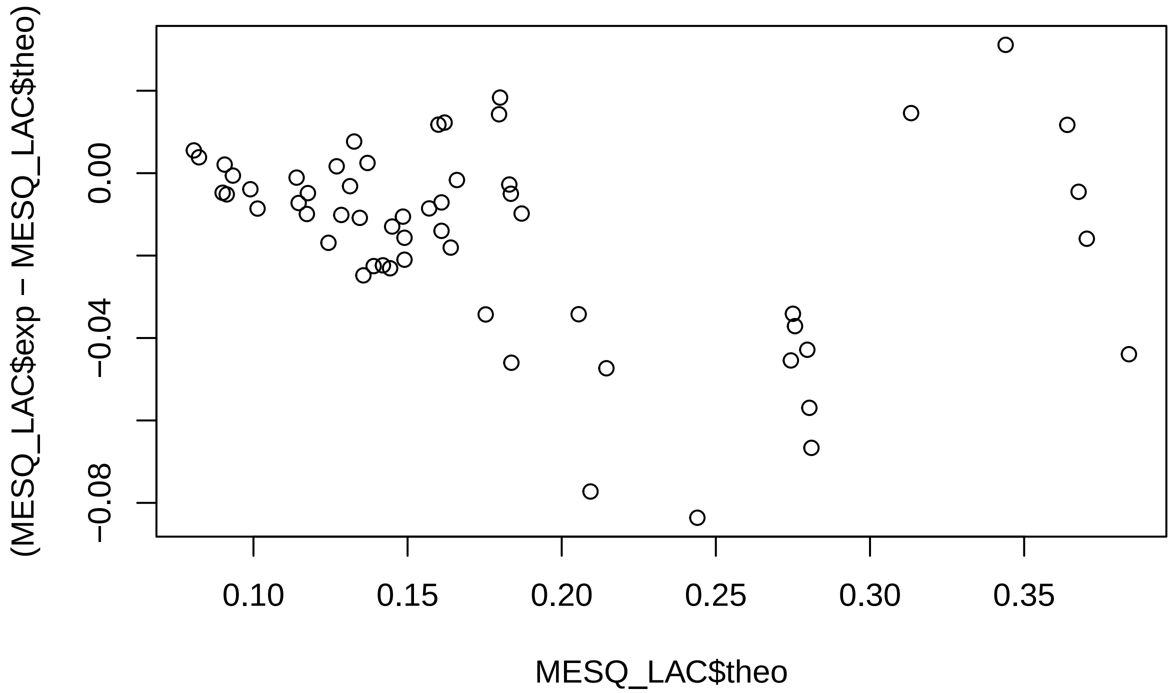


```
summary(lm((MESQ_LAC$exp-MESQ_LAC$theo)~MESQ_LAC$theo))
```

```
##
## Call:
## lm(formula = (MESQ_LAC$exp - MESQ_LAC$theo) ~ MESQ_LAC$theo)
##
## Residuals:
##       Min        1Q    Median        3Q       Max
## -0.063668 -0.011440  0.001925  0.011781  0.059411
##
## Coefficients:
##                 Estimate Std. Error t value Pr(>|t|)
## (Intercept)    0.0003958  0.0074120   0.053   0.9576
## MESQ_LAC$theo -0.0833497  0.0373593  -2.231   0.0298 *
## ---
## Signif. codes:  0 '***' 0.001 '**' 0.01 '*' 0.05 '.' 0.1 ' ' 1
##
## Residual standard error: 0.02259 on 55 degrees of freedom
## Multiple R-squared:  0.08299,    Adjusted R-squared:  0.06632
## F-statistic: 4.977 on 1 and 55 DF,  p-value: 0.02978
```

```
###NH4_LAC

MESQ_LAC=data.frame(NH4=as.double(),exp=as.double(), theo=as.double())
```

```r
# k=4
# i=1

for(k in 1:(length(attributes(DATA_ADD_DVH$CONDITION)$levels)-1)){

   data_lac=DATA_ADD_DVH[which(DATA_ADD_DVH$CONDITION
                  ==attributes(DATA_ADD_DVH$CONDITION)$levels[k]),]

for (i in 1:nrow(data_lac)){

  MESQ_LAC_x=data.frame(NH4=as.double(),
                        exp=as.double(),
                        theo=as.double())

  if(!(is.na(data_lac$NH4[i]))){

  MESQ_LAC_x[1,1]=data_lac$TIME_EXP[i]
  MESQ_LAC_x[1,2]=tab[[k*3-1]]$NH4[which(
    round(tab[[k*3-1]]$Time)
    ==round(data_lac$TIME_EXP[i]))]
  MESQ_LAC_x[1,3]=data_lac$NH4[i]

 MESQ_LAC=rbind.data.frame(MESQ_LAC,MESQ_LAC_x)
 MESQ_LAC_x=0

  }

  }
}

plot(MESQ_LAC$exp~MESQ_LAC$theo,
     main="Predict vs Measured Maximal Biomass Yield",
     xlab="Measured values", ylab="Modeled values")


abline(0,1, col="red")
```

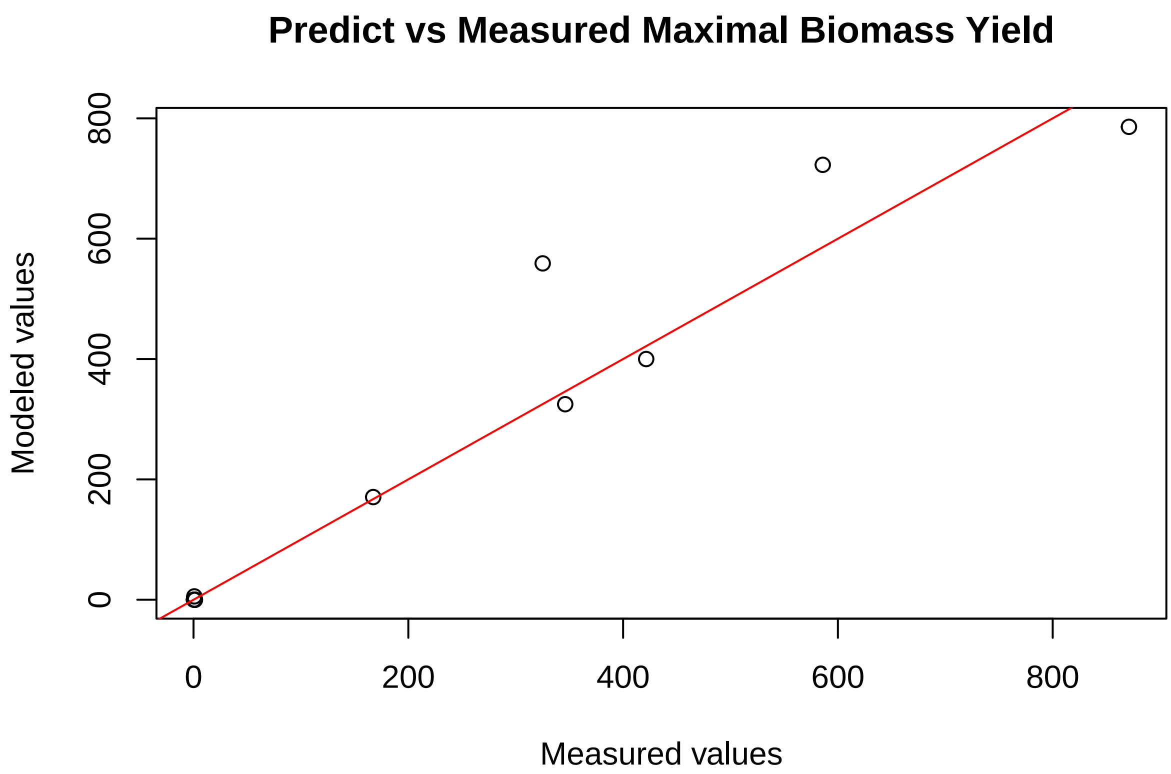


```r
print("AMMONIUM_LACTATE")
```

```
## [1] "AMMONIUM_LACTATE"
```

```r
##Is the regrssion good? Adj-R2=
summary(lm((MESQ_LAC$exp)~MESQ_LAC$theo))
```

```
##
## Call:
## lm(formula = (MESQ_LAC$exp) ~ MESQ_LAC$theo)
##
## Residuals:
##     Min      1Q  Median      3Q     Max
## -112.96  -25.14  -19.08  -15.67  212.24
##
## Coefficients:
##                Estimate Std. Error t value Pr(>|t|)
## (Intercept)    17.93748   32.66438   0.549    0.595
## MESQ_LAC$theo  1.01133    0.09139  11.066 6.23e-07 ***
## ---
## Signif. codes:  0 '***' 0.001 '**' 0.01 '*' 0.05 '.' 0.1 ' ' 1
##
## Residual standard error: 87.43 on 10 degrees of freedom
## Multiple R-squared:  0.9245, Adjusted R-squared:  0.917
## F-statistic: 122.5 on 1 and 10 DF,  p-value: 6.234e-07
```

```r
##Is prediction different from measured (is measured~prediction a 1:1 line)
##, p value>0.05 and slope value if not.
plot((MESQ_LAC$exp-MESQ_LAC$theo)~MESQ_LAC$theo)
```

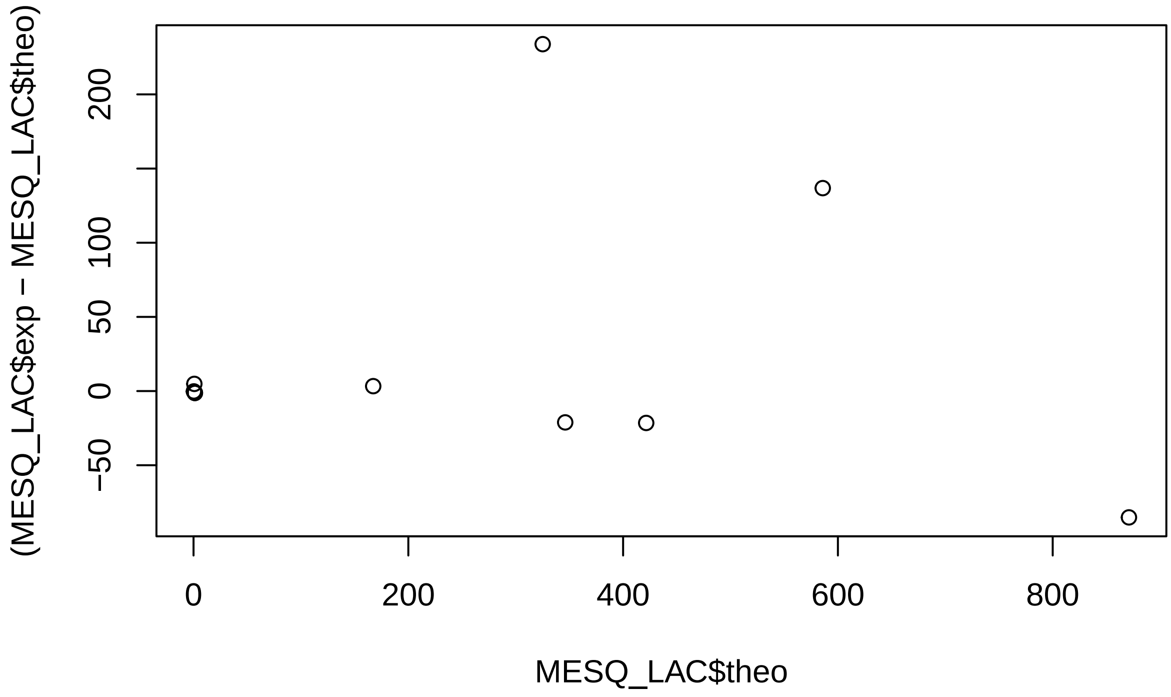


```r
summary(lm((MESQ_LAC$exp-MESQ_LAC$theo)~MESQ_LAC$theo))
```

```
##
## Call:
## lm(formula = (MESQ_LAC$exp - MESQ_LAC$theo) ~ MESQ_LAC$theo)
##
## Residuals:
##     Min      1Q  Median      3Q     Max
## -112.96  -25.14  -19.08  -15.67  212.24
##
## Coefficients:
##                Estimate Std. Error t value Pr(>|t|)
## (Intercept)    17.93748   32.66438   0.549    0.595
## MESQ_LAC$theo   0.01133    0.09139   0.124    0.904
##
## Residual standard error: 87.43 on 10 degrees of freedom
## Multiple R-squared:  0.001534,   Adjusted R-squared:  -0.09831
## F-statistic: 0.01536 on 1 and 10 DF,  p-value: 0.9038
```

###EXP CALIBRATION PYRUVATE ARA###

####MEDIA AND HEASPACE COMPOSITON INTIALIZATION

```
####Media

media=data.frame(
  conc_SO4_ext=20000,
  conc_NH4_ext=0,
  conc_Pyr_ext=30000,
  conc_H2S_ext=0,


  volume=10,
  volume_HS=volume_HS,### milliliter

  #work fine cell   alive= 0.1 BNF= 0.8
  OD_NH4=0.000,###0.0005

  OD_BNF=0.002, ###0.005

  OD_cell=0.002 ####0.005


)

media=cbind(media[,1:8],
            OD_dead=media$OD_cell-media$OD_NH4-media$OD_BNF,
            OD_cell=media[,9],
            OD_cell_active=(media$OD_NH4+media$OD_BNF))

media=cbind(media[,1:11],
            cell_active_percent=media$OD_cell_active/media$OD_cell,
            cell_BNF_percent=media$OD_BNF/media$OD_cell_active,
            v_NH4=0, v_BNF=0, lim_nut=0, HS_Et=0, acetylene=0,
            Timing_switch_2=0, Timing_inhibition=0)
```

####METABOLIC AND CELLULAR PARAMETERS

```
metabolism$mu_NH4=0.105
  metabolism$mu_BNF=0.044
metabolism$Pyr_SO4_ratio=4
    metabolism$Pyr_ATP=4
metabolism$lim_growth=0.03



metabolism$mu_BNF=metabolism$mu_BNF+metabolism$rdeath
metabolism$mu_NH4=metabolism$mu_NH4+metabolism$rdeath

metabolism$v_NH4=(metabolism$mu_NH4+constant)*metabolism$QNH4
metabolism$v_BNF=(metabolism$mu_BNF+constant)*metabolism$QBNF
```

####MODEL SIMULATION AND VALIDATION LABORATORY DATA##

####FIGURE_ADDITION EXP FIG1C&D DARNAJOUX_2022_SRBpaper###

```r
EXP_ADD=data.frame(addition=0,
                   OD_start=0.090,
                   acetylene=5.9, delay=3,
                   EXP_length=130)

conc=c(0,0,0,10,10,10,30,30,30,
       100,100,100,300,300,300,
       3000,3000,3000)

timing_sampling=c(0,3,6,9,29,51,77,121)

ODstart=c(0.062, 0.076, 0.099,0.062, 0.076,
          0.099,0.062, 0.076, 0.099,
          0.062, 0.076, 0.099,0.062,
          0.076, 0.099,0.062, 0.076, 0.099,
          0.062, 0.076, 0.099,0.062, 0.076, 0.099 )
##Average OD start of each replicate for
##the experiment 0.062, 0.076, 0.099

dat=list(data.frame())
tab=list(data.frame())
a="growth_"
b="Addition_"


for (i in 1:length(conc)){
  EXP_ADD$addition=conc[i]
  EXP_ADD$OD_start=ODstart[i]
  dat[[i]]=N_Addition_dvH(media=media,
                          metabolism=metabolism,
                          NH4_start=0, EXP_ADD=EXP_ADD,
                          graph=FALSE)

  names(dat)[i]=paste(a,conc[i],"")
  start=as.double(min(which(dat[[i]]$acetylene > 0)))

  tab1=data.frame(Time=as.double(0),
                  HS_Et=as.double(dat[[i]]$HS_Et[start]),
                  OD=as.double(dat[[i]]$OD_cell_active[start]),
                  NH4=as.double(dat[[i]]$conc_NH4_ext[start]),
                  Condition=as.factor(paste(b,conc[i],"")))

  for (j in 2:length(timing_sampling)){

    stop=start+timing_sampling[j]/0.5
    tab1=rbind(tab1,
               cbind.data.frame(Time=timing_sampling[j],
                 HS_Et=dat[[i]]$HS_Et[stop],
                 OD=as.double(dat[[i]]$OD_cell_active[stop]),
                 NH4=as.double(dat[[i]]$conc_NH4_ext[stop]),
                 Condition=paste(b,conc[i],"") ))
  }
```

```
  tab[[i]]=tab1

}


###############FIGURE ETHYLEN BIOMASS AMMONIUM
# pdf("2023_Figure5A.pdf", height = 11, width=7)

par(mfrow=c(3,1))
COMPARE=TRUE
BAND=T

#######PANEL A



xlim1=c(0,120)
ylim1=c(0,8000)
ylim2=c(0,1)
ylim3=c(0,3200)
###GROWTH
if(BAND==F){
  plot(HS_Et~Time, pch=16,
       data=tab[[1]], cex=3, col=col[1],
       lwd=2, type="l", xlim=xlim1,ylim=ylim1)

  for (i in 2: length(tab)){
    lines(HS_Et~Time, data=tab[[i]],
          pch=16,cex=2, lwd=2, col=col[i],
          xlim=xlim1,ylim=ylim2)

  }

}


if(BAND==T){
  ######UNCERTAINTY BAND
  # attach(tab[[1]], warn.conflicts = F)
  plot(HS_Et~Time, data=tab[[2]],  col=col[1],
       xlim=xlim1, type="l", ylim=ylim1, lwd=2,
       cex=3, xlab="Time since ARA start)",
       ylab="Headspace_Ethylene (ppm)",
       cex.lab=2, cex.axis=2)

  polygon(c(tab[[2]]$Time,rev(tab[[2]]$Time)),
          c(tab[[1]]$HS_Et,rev(tab[[3]]$HS_Et)),
          col=alpha(col[1], alpha=0.2),border=NA)



  for (i in 2:(length(conc)/3)){
    lines(HS_Et~Time, data=tab[[i*3-1]],
```

```
          col=col[i], type="l", xlim=xlim1,ylim=ylim1,
          lwd=2, cex=2, xlab="Time since ARA start)",
          ylab="Headspace_Ethylene (ppm)", cex.lab=2,
          cex.axis=2)

    polygon(c(tab[[i*3-1]]$Time,rev(tab[[i*3-1]]$Time)),
            c(tab[[i*3-2]]$HS_Et,rev(tab[[i*3]]$HS_Et)),
            col=alpha(col[i], alpha=0.2),border=NA)

  }

}

if(COMPARE==TRUE){
  points(ETHYLENE~TIME_EXP,
         data=DATA_ADD_DVH_FIG,
         pch=21, col=col[DATA_ADD_DVH_FIG$CONDITION],
         cex=3)

  arrows(DATA_ADD_DVH_FIG$TIME_EXP,
         DATA_ADD_DVH_FIG$ETHYLENE-DATA_ADD_DVH_FIG$SDEt,
         DATA_ADD_DVH_FIG$TIME_EXP,
         DATA_ADD_DVH_FIG$ETHYLENE+DATA_ADD_DVH_FIG$SDEt,
         code=0,col=col[DATA_ADD_DVH_FIG$CONDITION],
         lwd = 2 )


}


if(BAND==F){
  plot(OD~Time, pch=16, data=tab[[1]],
       cex=3, col=col[1],type="l",
       lwd=2, xlim=xlim1,ylim=ylim2)

  for (i in 2: length(tab)){
    lines(OD~Time, data=tab[[i]],
          pch=16,cex=2, col=col[i],
          lwd=2, xlim=xlim1,ylim=ylim2)

  }
  lines(OD~Time, data=tab0, cex=2,
        xlim=c(0,285), ylim=c(0,1),
        pch=16, lwd=2, col=col[4],
        xlim=xlim1,ylim=ylim2)

  if(COMPARE==TRUE){
    points(OD600~TIME_EXP,
           data=DATA_ADD_DVH_FIG,
           pch=21, col=col[DATA_ADD_DVH_FIG$CONDITION],
           cex=3)

    arrows(DATA_ADD_DVH_FIG$TIME_EXP,
```

```
          DATA_ADD_DVH_FIG$OD600-DATA_ADD_DVH_FIG$SD,
          DATA_ADD_DVH_FIG$TIME_EXP,
          DATA_ADD_DVH_FIG$OD600+DATA_ADD_DVH_FIG$SD,
          code=0,col=col[DATA_ADD_DVH_FIG$CONDITION],
          lwd = 2 )
  }

}

if(BAND==T){
  ######UNCERTAINTY BAND

  plot(OD~Time, pch=16, data=tab[[2]],
       cex=3, col=col[1],type="l", lwd=2,
       xlim=xlim1,ylim=ylim2, xlab="Time since ARA start)",
       ylab="OD600", cex.lab=2, cex.axis=2)

  polygon(c(tab[[2]]$Time,rev(tab[[2]]$Time)),
          c(tab[[1]]$OD,rev(tab[[3]]$OD)),
          col=alpha(col[1], alpha=0.2),border=NA)



  for (i in 1:(length(conc)/3)){
    lines(OD~Time, data=tab[[i*3-1]],
          col=col[i],xlim=xlim1,ylim=ylim2,type="l",
          lwd=2, cex=2, xlab="Time since ARA start)",
          ylab="OD600", cex.lab=2, cex.axis=2)
    polygon(c(tab[[i*3-1]]$Time,rev(tab[[i*3-1]]$Time)),
            c(tab[[i*3-2]]$OD,rev(tab[[i*3]]$OD)),
            col=alpha(col[i], alpha=0.2),border=NA)

  }




if(COMPARE==TRUE){
  points(OD600~TIME_EXP,
         data=DATA_ADD_DVH_FIG,
         pch=21, col=col[DATA_ADD_DVH_FIG$CONDITION],
         cex=3)

  arrows(DATA_ADD_DVH_FIG$TIME_EXP,
         DATA_ADD_DVH_FIG$OD600-DATA_ADD_DVH_FIG$SD,
         DATA_ADD_DVH_FIG$TIME_EXP,
         DATA_ADD_DVH_FIG$OD600+DATA_ADD_DVH_FIG$SD,
         code=0,col=col[DATA_ADD_DVH_FIG$CONDITION],
         lwd = 2 )
}


}
```

```
#####FIGURE 1D DARNAJOUX ET AL 2022 PANEL C

##############comparison FIGURE 1C SRB DARNAJOUX 2022


conc_test=c(0,10,30,100,300, 3000)   ####CONCENTRATION OF NH4 TO BE ADDED

tabaa=tab




for (i in 1:(length(tabaa))){
  tabaa[[i]]=cbind(tabaa[[i]],
                   slope=as.double(NA),
                   Time_slope=as.double(NA),
                   rel_slope=as.double(NA))

  for (j in 1: length(tabaa[[i]])-1){
    dataa=tabaa[[i]]
    tabaa[[i]]$slope[j]=(dataa$HS_Et[j+1]-dataa$HS_Et[j])/(dataa$Time[j+1]-dataa$Time[j])
        #ppm.h-1
    tabaa[[i]]$Time_slope[j]=(dataa$Time[j+1]-dataa$Time[2])
  }

}

for (i in 1:(length(tabaa))){
  for(j in 1: length(tabaa[[i]])-1){
    tabaa[[i]]$rel_slope[j]= tabaa[[i]]$slope[j]/mean(c(tabaa[[1]]$slope[j],
                             tabaa[[2]]$slope[j], tabaa[[3]]$slope[j]))
  }
}


relslope=list(data.frame())
c="T_"

for (j in 1:(length(tabaa[[1]]))){
  relslope[[j]]<-data.frame(rel_slope=double(),
                            Time=factor(), NH4=double(),
                            NH4init=double(),
                            stringsAsFactors=FALSE)

  relslope[[j]]<-data.frame(rel_slope=as.double(tabaa[[1]]$rel_slope[j]),
          Time=as.factor((paste(c,
            tabaa[[1]]$Time_slope[j]))),
            NH4=as.double(tabaa[[1]]$NH4[j]),
            NH4init=as.double(conc[1]))

  for ( i in 2:(length(tabaa))){
    relslope[[j]]=rbind.data.frame(relslope[[j]],
       list(rel_slope=tabaa[[i]]$rel_slope[j],
```

```r
            Time=(paste(c, tabaa[[i]]$Time_slope[j])),
                    NH4=tabaa[[i]]$NH4[j],
                    NH4init=conc[i]))

  }

  names(relslope)[j]=paste(c, tabaa[[i]]$Time_slope[j])

}
relslope[[length(relslope)]]<-NULL



####AVERAGE VALUE AND SD###

relslope_mean=list(data.frame())



for (i in 1:length(relslope)){


  mean_slope=tapply(relslope[[i]]$rel_slope,
                    as.factor(relslope[[i]]$NH4init),
                    FUN=mean)

  sd_slope=tapply(relslope[[i]]$rel_slope,
                  as.factor(relslope[[i]]$NH4init),
                  FUN=sd)

  relslope_mean[[i]]=data.frame(mean_rel_slope=as.double(mean_slope),
                   SD=as.double(sd_slope),
                   Time=as.factor(relslope[[i]]$Time[1]),
                   NH4init=as.double(conc_test))
  names(relslope_mean)[i]=names(relslope)[i]
}



#######Bande de confidence



attach(relslope_mean[[2]], warn.conflicts = F)
plot(mean_rel_slope~NH4init,
     data=relslope_mean[[2]],
     xlim=c(0,3000), type="l",
     lwd=2,ylim=c(0,1.5), col=col[2],
     pch=16, cex=3, xlab="[NH4+]initial (uM)",
     ylab="Relative BNF (% of Control)",
     cex.lab=2, cex.axis=2)

polygon(c(NH4init,rev(NH4init)),
```

```
        c(mean_rel_slope-SD,rev(mean_rel_slope+SD)),
        col=alpha(col[2], alpha=0.2),border=NA)



for (i in 3:4){
  attach(relslope_mean[[i]], warn.conflicts = F)
  lines(mean_rel_slope~NH4init,
        xlim=c(0,300),
        type="l", lwd=2,
        ylim=c(0,1.5), col=col[i],pch=16, cex=2)
  polygon(c(NH4init,rev(NH4init)),
          c(mean_rel_slope-SD,rev(mean_rel_slope+SD)),
          col=alpha(col[i], alpha=0.2),border=NA)

}



DATA_ARA_DvH_ADD <- read.csv("20221025_DATA_ARA_DvH_ADD.xlsx.csv",
                             sep = ";")
#
DATA_ARA_DvH_ADD$TIME_SLOT<-factor(DATA_ARA_DvH_ADD$TIME_SLOT,
              levels=c("0hr", "3hr", "6hr", "26hr", ">45hr"))

dat1=as.data.frame(DATA_ARA_DvH_ADD)

attach(dat1, warn.conflicts = F)
for (i in 2:(length(levels(DATA_ARA_DvH_ADD$TIME_SLOT))-1)){
  dat2=dat1[which(DATA_ARA_DvH_ADD$TIME_SLOT==levels(DATA_ARA_DvH_ADD$TIME_SLOT)[i]),]
  points(Rel_slope/100~NH4init,
         data=dat2, xlim=c(0,300),
         ylim=c(0,1.5), col=col[i],
         pch=16, cex=3)

}
legend(750,1.5, c("DAD-CM_ARA", "Measured"),
       col=c(alpha(col[2], alpha=0.5),col[2]),
       lty=c(1,0),lwd=2, pch=c(15, 16),cex=1)
```

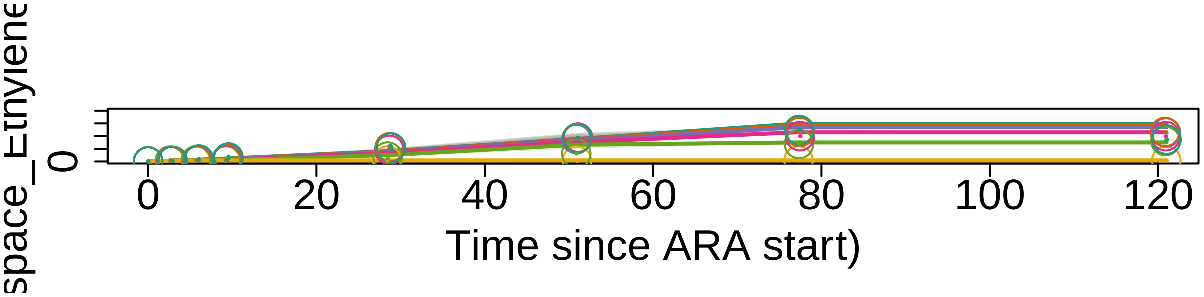

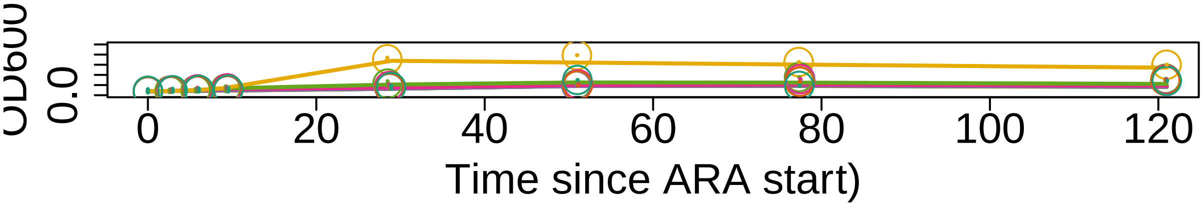

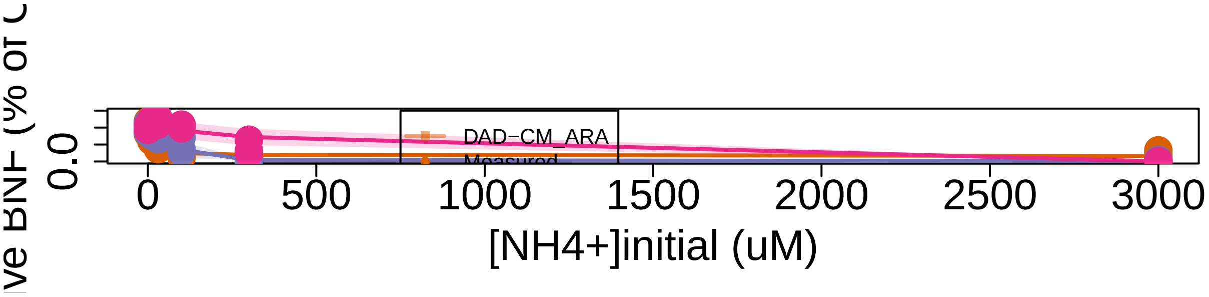


```
detach(dat1)
#




# dev.off()
```

####FITTING ESTIMATE####

```
###MESSQ for DATA addition


###Ethylene

MESQ_LAC=data.frame(NH4=as.double(),exp=as.double(), theo=as.double())


for(k in 1:(length(attributes(DATA_ADD_DVH_FIG$CONDITION)$levels))){

   data_lac=DATA_ADD_DVH_FIG[which(DATA_ADD_DVH_FIG$CONDITION
                  ==attributes(DATA_ADD_DVH_FIG$CONDITION)$levels[k]),]

for (i in 1:nrow(data_lac)){

  MESQ_LAC_x=data.frame(NH4=as.double(),exp=as.double(),
```

```
                        theo=as.double())



  MESQ_LAC_x[1,1]=data_lac$TIME_EXP[i]
  MESQ_LAC_x[1,2]=tab[[k*3-1]]$HS_Et[i]
  MESQ_LAC_x[1,3]=data_lac$ETHYLENE[i]

 MESQ_LAC=rbind.data.frame(MESQ_LAC,MESQ_LAC_x)
 MESQ_LAC_x=0




  }
}

plot(MESQ_LAC$exp~MESQ_LAC$theo,
     main="Predict vs Measured Maximal Biomass Yield",
     xlab="Measured values", ylab="Modeled values")


abline(0,1, col="red")
```

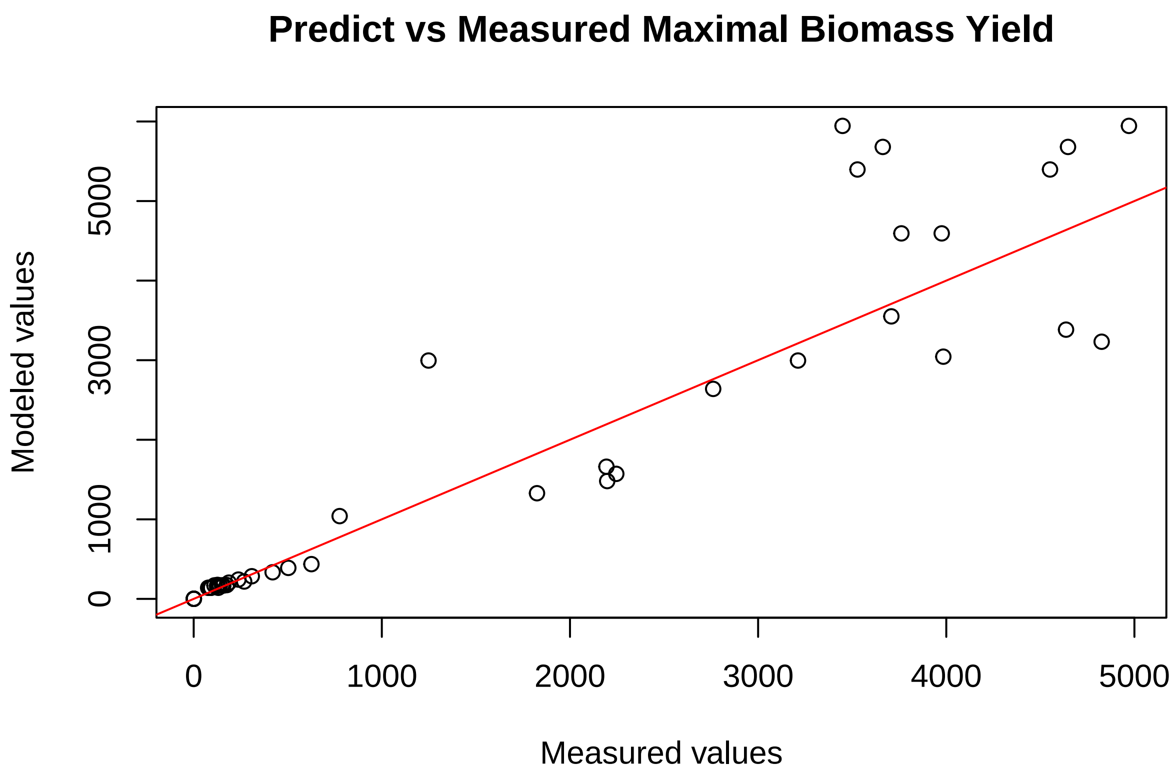


```
print("ETHYLENE")
```

```
## [1] "ETHYLENE"
```

```r
##Is the regrssion good? Adj-R2=
summary(lm((MESQ_LAC$exp)~MESQ_LAC$theo))
```

```
##
## Call:
## lm(formula = (MESQ_LAC$exp) ~ MESQ_LAC$theo)
##
## Residuals:
##      Min       1Q   Median       3Q      Max
## -1973.57  -141.46   -15.93    35.04  2220.12
##
## Coefficients:
##                Estimate Std. Error t value Pr(>|t|)
## (Intercept)    16.18725  141.56310   0.114    0.909
## MESQ_LAC$theo   1.07528    0.06221  17.286   <2e-16 ***
## ---
## Signif. codes:  0 '***' 0.001 '**' 0.01 '*' 0.05 '.' 0.1 ' ' 1
##
## Residual standard error: 748.8 on 46 degrees of freedom
## Multiple R-squared:  0.8666, Adjusted R-squared:  0.8637
## F-statistic: 298.8 on 1 and 46 DF,  p-value: < 2.2e-16
```

```r
##Is prediction different from measured (is measured~prediction a 1:1 line),
## p value>0.05 and slope value if not.
plot((MESQ_LAC$exp-MESQ_LAC$theo)~MESQ_LAC$theo)
```

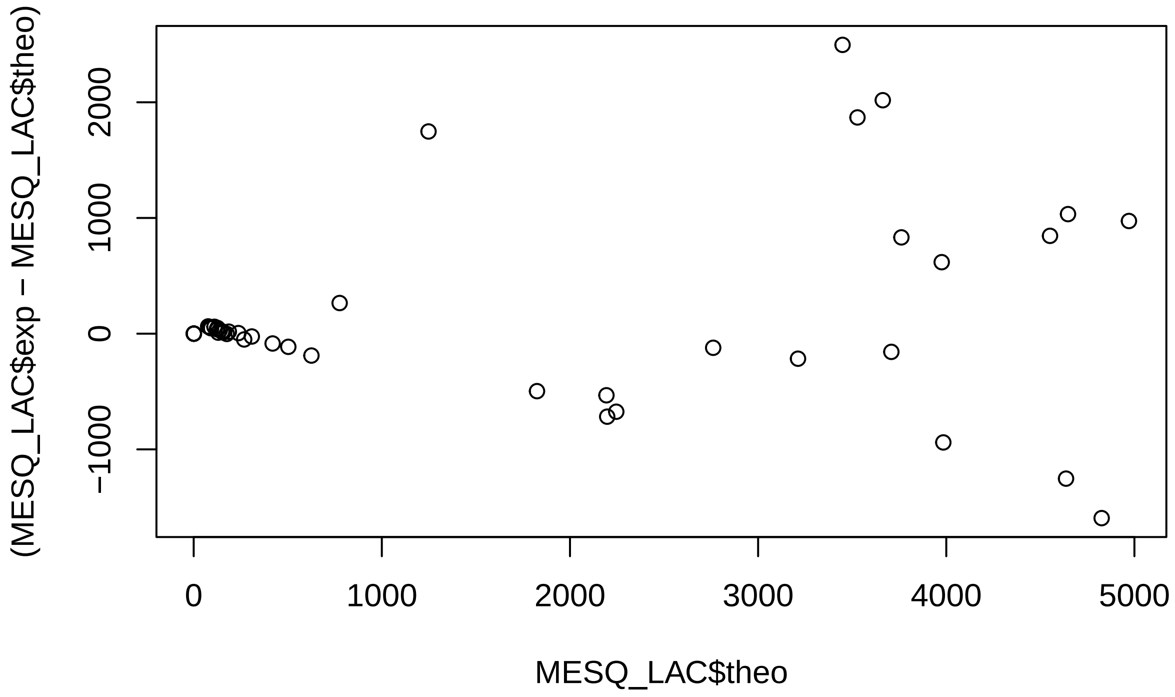


```
summary(lm((MESQ_LAC$exp-MESQ_LAC$theo)~MESQ_LAC$theo))
```

```
##
## Call:
## lm(formula = (MESQ_LAC$exp - MESQ_LAC$theo) ~ MESQ_LAC$theo)
##
## Residuals:
##      Min       1Q   Median       3Q      Max
## -1973.57  -141.46   -15.93    35.04  2220.12
##
## Coefficients:
##                Estimate Std. Error t value Pr(>|t|)
## (Intercept)    16.18725  141.56310   0.114    0.909
## MESQ_LAC$theo   0.07528    0.06221   1.210    0.232
##
## Residual standard error: 748.8 on 46 degrees of freedom
## Multiple R-squared:  0.03086,    Adjusted R-squared:  0.009789
## F-statistic: 1.465 on 1 and 46 DF,  p-value: 0.2324
```

```
###OD_LAC

MESQ_LAC=data.frame(NH4=as.double(),exp=as.double(), theo=as.double())


for(k in 1:(length(attributes(DATA_ADD_DVH_FIG$CONDITION)$levels))){
```

```
   data_lac=DATA_ADD_DVH_FIG[which(DATA_ADD_DVH_FIG$CONDITION
                        ==attributes(DATA_ADD_DVH_FIG$CONDITION)$levels[k]),]

for (i in 1:nrow(data_lac)){

  MESQ_LAC_x=data.frame(NH4=as.double(),exp=as.double(),
                        theo=as.double())



  MESQ_LAC_x[1,1]=data_lac$TIME_EXP[i]
  MESQ_LAC_x[1,2]=tab[[k*3-1]]$OD[i]
  MESQ_LAC_x[1,3]=data_lac$OD600[i]

 MESQ_LAC=rbind.data.frame(MESQ_LAC,MESQ_LAC_x)
 MESQ_LAC_x=0

  }
}

plot(MESQ_LAC$exp~MESQ_LAC$theo,
     main="Predict vs Measured Maximal Biomass Yield",
     xlab="Measured values", ylab="Modeled values")


abline(0,1, col="red")
```

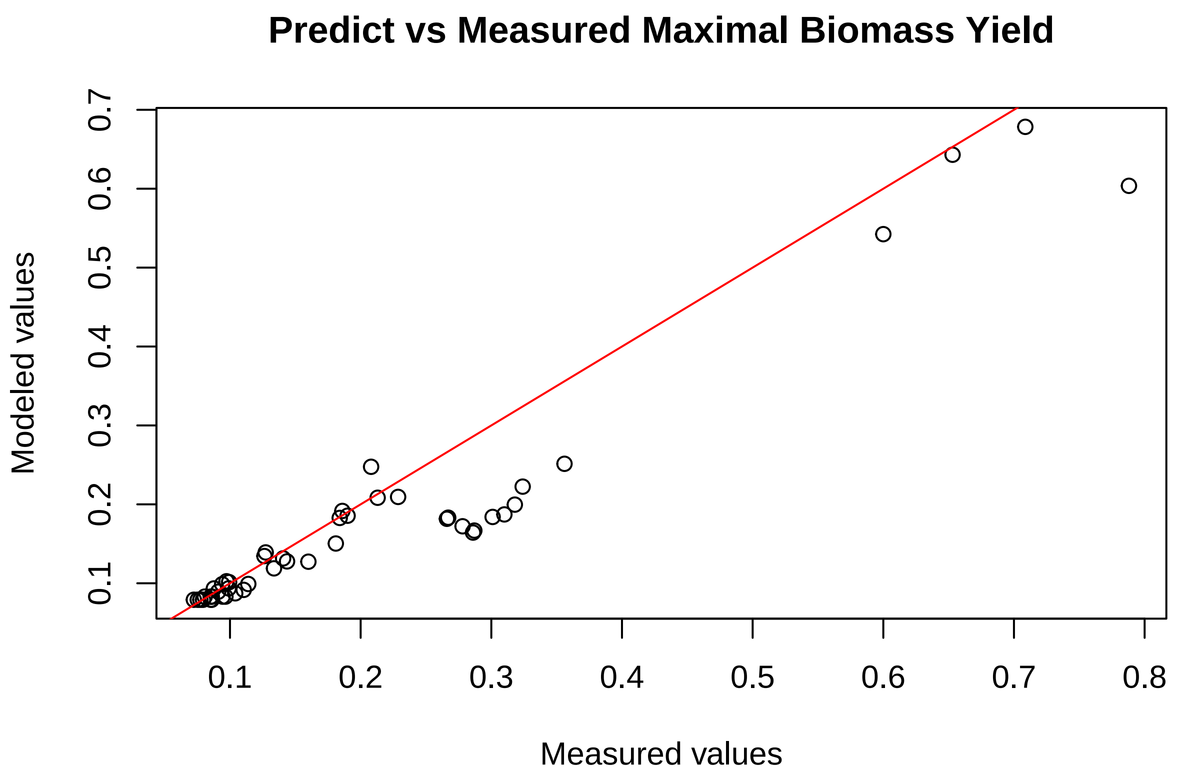


```r
print("BIOMASS")
```

```
## [1] "BIOMASS"
```

```r
##Is the regrssion good? Adj-R2=
summary(lm((MESQ_LAC$exp)~MESQ_LAC$theo))
```

```
##
## Call:
## lm(formula = (MESQ_LAC$exp) ~ MESQ_LAC$theo)
##
## Residuals:
##       Min        1Q    Median        3Q       Max
## -0.076074 -0.005864  0.006939  0.016116  0.101461
##
## Coefficients:
##               Estimate Std. Error t value Pr(>|t|)
## (Intercept)   0.005531   0.009077   0.609    0.545
## MESQ_LAC$theo 0.820906   0.034199  24.003   <2e-16 ***
## ---
## Signif. codes:  0 '***' 0.001 '**' 0.01 '*' 0.05 '.' 0.1 ' ' 1
##
## Residual standard error: 0.03979 on 46 degrees of freedom
## Multiple R-squared:  0.9261, Adjusted R-squared:  0.9245
## F-statistic: 576.2 on 1 and 46 DF,  p-value: < 2.2e-16
```

```
##Is prediction different from measured (is measured~prediction a 1:1 line)
##, p value>0.05 and slope value if not.
plot((MESQ_LAC$exp-MESQ_LAC$theo)~MESQ_LAC$theo)
```

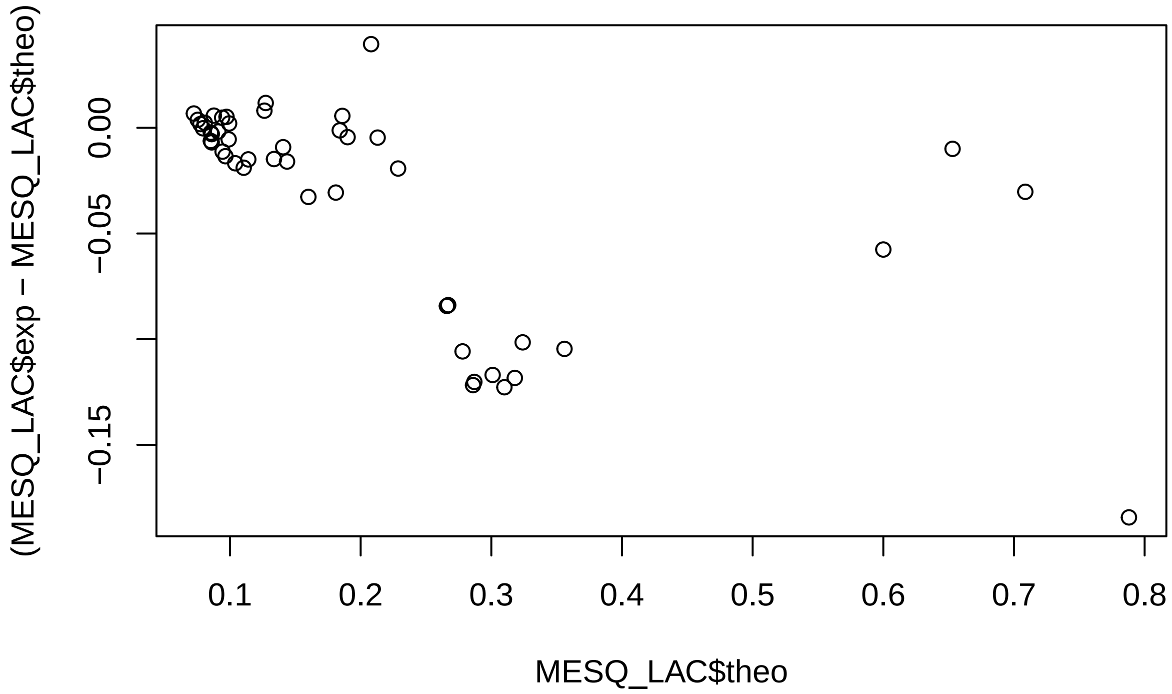


```
summary(lm((MESQ_LAC$exp-MESQ_LAC$theo)~MESQ_LAC$theo))
```

```
##
## Call:
## lm(formula = (MESQ_LAC$exp - MESQ_LAC$theo) ~ MESQ_LAC$theo)
##
## Residuals:
##       Min        1Q    Median        3Q       Max
## -0.076074 -0.005864  0.006939  0.016116  0.101461
##
## Coefficients:
##                Estimate Std. Error t value Pr(>|t|)
## (Intercept)    0.005531   0.009077   0.609    0.545
## MESQ_LAC$theo -0.179094   0.034199  -5.237 3.96e-06 ***
## ---
## Signif. codes:  0 '***' 0.001 '**' 0.01 '*' 0.05 '.' 0.1 ' ' 1
##
## Residual standard error: 0.03979 on 46 degrees of freedom
## Multiple R-squared:  0.3735, Adjusted R-squared:  0.3599
## F-statistic: 27.42 on 1 and 46 DF,  p-value: 3.955e-06
```

```
###%ACTIVITY

MESQ_LAC=data.frame(NH4=as.double(),exp=as.double(), theo=as.double())

# k=1
# i=1

for(k in 1:(length(attributes(DATA_ARA_DvH_ADD$TIME_SLOT)$levels)-1)){

   data_lac=DATA_ARA_DvH_ADD[which(DATA_ARA_DvH_ADD$TIME_SLOT
                         ==attributes(DATA_ARA_DvH_ADD$TIME_SLOT)$levels[k]),]

for (i in 1:nrow(data_lac)){

  MESQ_LAC_x=data.frame(NH4=as.double(),exp=as.double(),
                        theo=as.double())



  MESQ_LAC_x[1,1]=paste(data_lac$TIME_SLOT[i],"&", data_lac$NH4init[i])
  MESQ_LAC_x[1,2]=relslope_mean[[k]]$mean_rel_slope[
    which(relslope_mean[[k]]$NH4init
          ==data_lac$NH4init[i])]*100
  MESQ_LAC_x[1,3]=data_lac$Rel_slope[i]

 MESQ_LAC=rbind.data.frame(MESQ_LAC,MESQ_LAC_x)
 MESQ_LAC_x=0

  }


}

plot(MESQ_LAC$exp~MESQ_LAC$theo,
     main="Predict vs Measured Maximal Biomass Yield",
     xlab="Measured values", ylab="Modeled values")


abline(0,1, col="red")
```

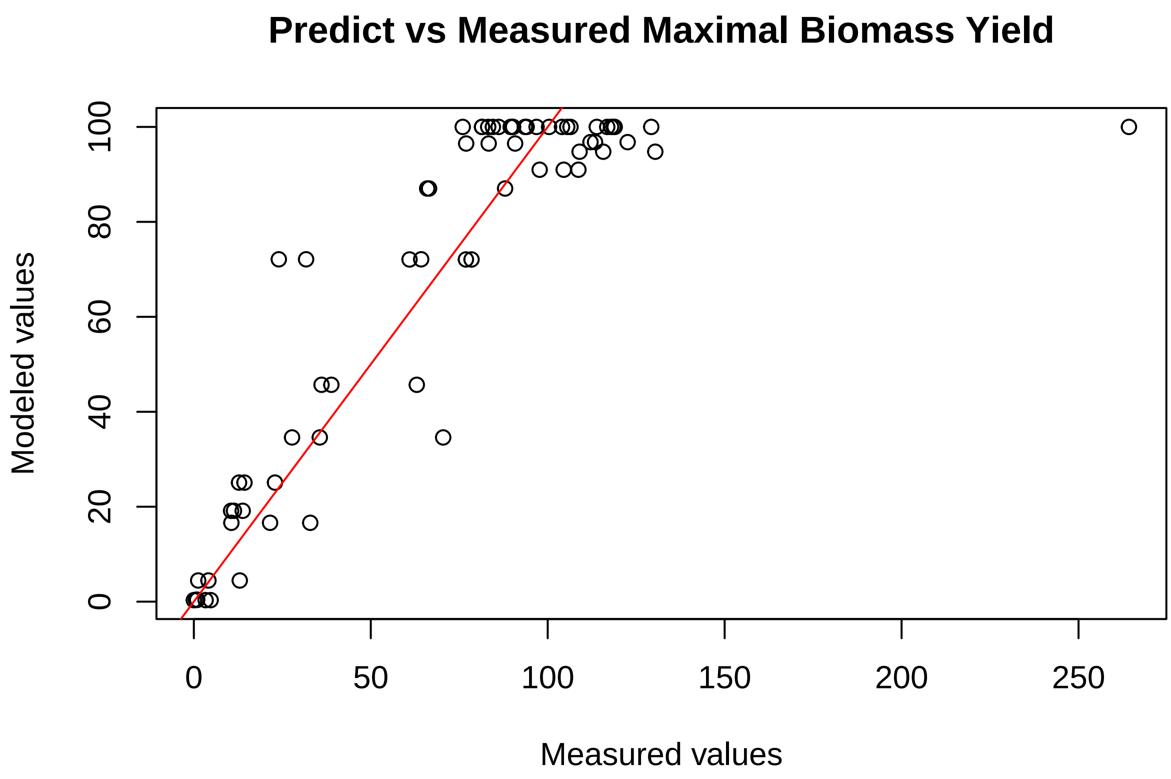


```
print("%ACTIVITY")
```

```
## [1] "%ACTIVITY"
```

```
##Is the regrssion good? Adj-R2=
summary(lm((MESQ_LAC$exp)~MESQ_LAC$theo))
```

```
##
## Call:
## lm(formula = (MESQ_LAC$exp) ~ MESQ_LAC$theo)
##
## Residuals:
##     Min      1Q  Median      3Q     Max
## -99.355 -10.464  -0.172  15.089  35.655
##
## Coefficients:
##                Estimate Std. Error t value Pr(>|t|)
## (Intercept)    20.17755    4.36345   4.624  1.8e-05 ***
## MESQ_LAC$theo  0.67806    0.05186  13.075  < 2e-16 ***
## ---
## Signif. codes:  0 '***' 0.001 '**' 0.01 '*' 0.05 '.' 0.1 ' ' 1
##
## Residual standard error: 20.38 on 66 degrees of freedom
## Multiple R-squared:  0.7215, Adjusted R-squared:  0.7173
## F-statistic:   171 on 1 and 66 DF,  p-value: < 2.2e-16
```

```r
##Is prediction different from measured (is measured~prediction a 1:1 line)
##, p value>0.05 and slope value if not.
plot((MESQ_LAC$exp-MESQ_LAC$theo)~MESQ_LAC$theo)
```

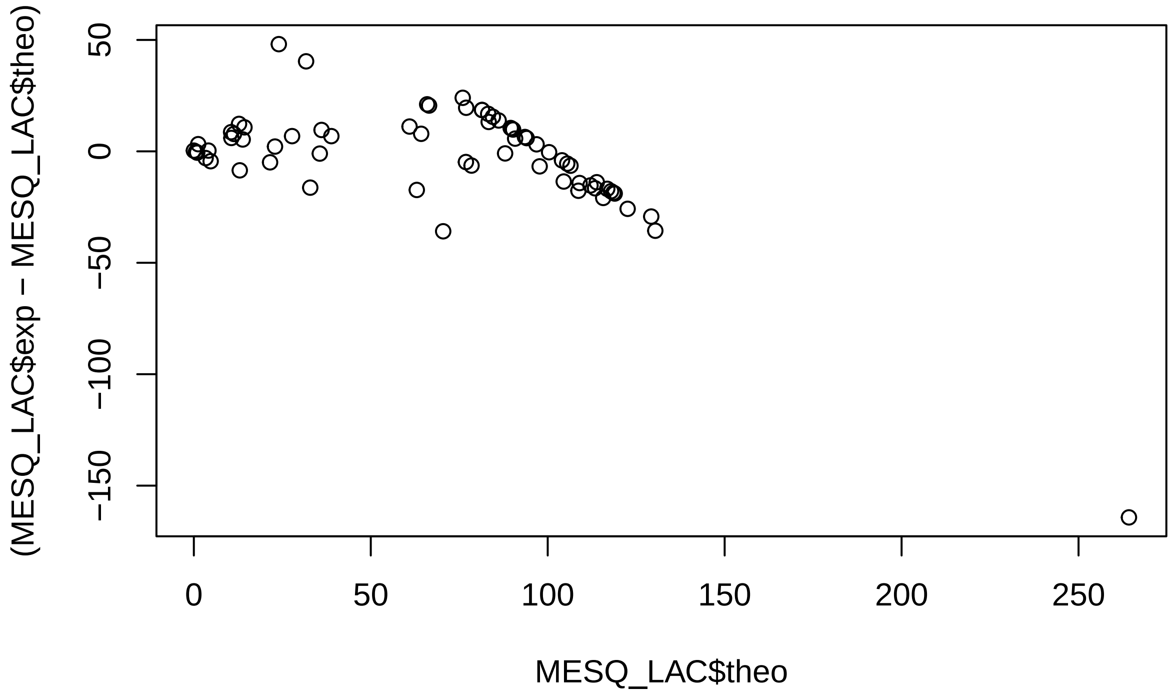


```r
summary(lm((MESQ_LAC$exp-MESQ_LAC$theo)~MESQ_LAC$theo))
```

```
##
## Call:
## lm(formula = (MESQ_LAC$exp - MESQ_LAC$theo) ~ MESQ_LAC$theo)
##
## Residuals:
##     Min      1Q  Median      3Q     Max
## -99.355 -10.464  -0.172  15.089  35.655
##
## Coefficients:
##               Estimate Std. Error t value Pr(>|t|)
## (Intercept)   20.17755    4.36345   4.624 1.80e-05 ***
## MESQ_LAC$theo -0.32194    0.05186  -6.208 4.03e-08 ***
## ---
## Signif. codes:  0 '***' 0.001 '**' 0.01 '*' 0.05 '.' 0.1 ' ' 1
##
## Residual standard error: 20.38 on 66 degrees of freedom
## Multiple R-squared:  0.3687, Adjusted R-squared:  0.3591
## F-statistic: 38.54 on 1 and 66 DF,  p-value: 4.032e-08
```

###GROWTH WITH AMMONIUM AND NO ACETYLENE ####METABOLIC AND CELLULAR PARAMETERS FOR GROWTH (NO ACETYLENE)###

```
metabolism$mu_NH4=0.105
  metabolism$mu_BNF=0.044
metabolism$Pyr_SO4_ratio=4
    metabolism$Pyr_ATP=4
metabolism$lim_growth=0.07


metabolism$mu_BNF=metabolism$mu_BNF+metabolism$rdeath
metabolism$mu_NH4=metabolism$mu_NH4+metabolism$rdeath

metabolism$v_NH4=metabolism$mu_NH4*metabolism$QNH4
metabolism$v_BNF=metabolism$mu_BNF*metabolism$QBNF
```

####EXPERIMENT BATCH CULTURE SIMULATION AND GROWTH RATE MEASUREMENT

```
par(mfrow=c(2,2))
conc=c(0,10,30,40,50,60,75,100,150,
       200,300,400,500,600,700,800,900,1000,1300,
       1500,1700,2000,2200,2300,2400,2500,2800,3000)

tab=data.frame(conc_NH4=as.double(),mu_NH4_true=as.double(),
               mu_BNF_true=as.double(), mu_NH4_app=as.double(),
               mu_BNF_app=as.double())
dat=list(data.frame())
a="growth_"

for (i in 1:length(conc)){
  media$conc_NH4_ext=metabolism$Conc_0+conc[i]
  dat[[i]]=Culture_lim(media, metabolism, method="addition")

  tab[i,]=c(conc[i],growthrate(dat[[i]],graph=F))
  #Graph =T shows the fitting for each individual curve in normal coordinate
  names(dat)[i]=paste(a,conc[i],"")
}

##Force growth rate NH4 to 0 when no ammonium present
tab$mu_NH4_true[1]=0
tab$mu_NH4_app[1]=0
```

####FIGURE 2 A,B,C

```
# pdf("2023_Figure2.pdf", height = 14, width=8) #unquote for files saving


# par(mfrow=c(3,1), mar=c(5,5,5,1))
par(mfrow=c(3,1), mar=c(5,5,1,1))

###PANEL A
plot(tab$mu_NH4_app~tab$conc_NH4,
     col=brewer.pal(n=9,name="RdBu")[1],
```

```
     ylim=c(0,0.12), xlim=c(0,3000),
     type="line", lwd=5,
     xlab="[NH4+] initial (uM)",
     ylab="Growth rate (hr-1)",
     cex.axis=2, cex.lab=2)
```

```
## Warning in plot.xy(xy, type, ...): Le type de graphique 'line' sera tronqué au
## premier caractère
```

```
lines(tab$mu_BNF_app ~ tab$conc_NH4,
      col=brewer.pal(n=9,name="RdBu")[9],
      lwd=5)

points(DATA$GR_NH4~DATA$`[NH4]`,
       pch=c(16,15,17)[DATA$EXP],
       cex=3,
       col=brewer.pal(n=8,
                  name="Dark2")[DATA$EXP])
arrows(DATA$`[NH4]`,
       DATA$GR_NH4-DATA$SD_GR_NH4,DATA$`[NH4]`,
       DATA$GR_NH4+DATA$SD_GR_NH4,code=0,
       col=brewer.pal(n=8, name="Dark2")[DATA$EXP],
       lwd = 2 )

points(DATA$GR_BNF~DATA$`[NH4]`,
       pch=c(21, 22,24)[DATA$EXP],
       cex=3,lwd=2,
       col=brewer.pal(n=8, name="Dark2")[DATA$EXP])
arrows(DATA$`[NH4]`,
       DATA$GR_BNF-DATA$SD_GR_BNF,DATA$`[NH4]`,
       DATA$GR_BNF+DATA$SD_GR_BNF,code=0,
       col=brewer.pal(n=8, name="Dark2")[DATA$EXP],
       lwd=2 )

# legend(2000,0.05,legend = c("Rep1", "Rep2","Rep3"),
#pch=c(21,22,24), cex=1.5,lwd=2,bty = "n")

legend(2000,0.09,legend = c("NH4+-trophy", "N2-trophy"),
       lty = c(1,1), pch=c(16, 21),
       cex=1.5, lwd=3,bty = "n",
       col=brewer.pal(n=9,name="RdBu")[c(1,9)])


DvH=data.frame(NH4_init=as.double(),
               NH4_biomass=as.double(),
               BNF_biomass=as.double(),
               Tot_biomass=as.double(),
               H2S=as.double(), SO4=as.double(),
               Dead_biomass=as.double(),
               lim_nut=as.double(),
               Acetate=as.double())

for (i in 1:length(dat)) {
```

```
  data=dat[[i]]

  attach(data, warn.conflicts = F)
  DvH[i,]=cbind(NH4_init=as.double(conc[i]),
              NH4_biomass=as.double(max(data$OD_NH4)),
              BNF_biomass=as.double(max(data$OD_BNF)),
              Tot_biomass=as.double(max(data$OD_cell_active)),
              H2S=as.double(max(data$conc_H2S_ext)),
              SO4=as.double(min(data$conc_SO4_ext)),
              Dead_biomass=as.double(max(data$OD_dead)),
              lim_nut=as.double(max(data$lim_nut)),
    Acetate=as.double(data$conc_Pyr_ext[1]-min(data$conc_Pyr_ext)))


}
attach(DvH, warn.conflicts = F)


###Panel B

plot(Tot_biomass ~ NH4_init, ylim=c(0,0.9),
     type="line",col=brewer.pal(n=8,name="Dark2")[8],
     lwd=5,
     xlab="[NH4+] initial (uM)",
     ylab="Biomass density (OD600)",
     cex.axis=2, cex.lab=2)
```

```
## Warning in plot.xy(xy, type, ...): Le type de graphique 'line' sera tronqué au
## premier caractère
```

```
lines(NH4_biomass~ NH4_init,
      col=brewer.pal(n=9,name="RdBu")[1],
      lwd=5)
points(DATA$ODMax~DATA$`[NH4]`,
       pch=c(16,15,17)[DATA$EXP],
       cex=3,lwd=2,
       col=brewer.pal(n=8, name="Dark2")[DATA$EXP])

arrows(DATA$`[NH4]`,
       DATA$ODMax-DATA$OD_SD,DATA$`[NH4]`,
       DATA$ODMax+DATA$OD_SD,code=0,
       col=brewer.pal(n=8, name="Dark2")[DATA$EXP],
       lwd=2 )

points(DATA$OD_NH4~DATA$`[NH4]`,
       pch=c(21,22,24)[DATA$EXP],cex=3,
       lwd=2,col=brewer.pal(n=8, name="Dark2")[DATA$EXP])
arrows(DATA$`[NH4]`,
       DATA$OD_NH4-DATA$SD_OD_NH4,DATA$`[NH4]`,
       DATA$OD_NH4+DATA$SD_OD_NH4,code=0,
       col=brewer.pal(n=8, name="Dark2")[DATA$EXP],
       lwd=2)
```

```r
# legend(2000,0.6,legend = c("NH4+-trophy", "N2-trophy"),
##lty = c(1,1), pch=c(16, 21),
##cex=1.5, lwd=3,bty = "n",
##col=brewer.pal(n=9,name="RdBu")[c(1,9)])

legend(2000,0.3,legend = c("Rep1", "Rep2","Rep3"),
       pch=c(21,22,24), cex=1.5, lwd=3,bty = "n")

summary(lm(NH4_biomass~ NH4_init+0, data=DvH[1:24,]))
```

```
##
## Call:
## lm(formula = NH4_biomass ~ NH4_init + 0, data = DvH[1:24, ])
##
## Residuals:
##       Min        1Q    Median        3Q       Max
## -0.012365  0.001737  0.001893  0.002028  0.002229
##
## Coefficients:
##           Estimate Std. Error t value Pr(>|t|)
## NH4_init 3.489e-04  6.434e-07   542.3   <2e-16 ***
## ---
## Signif. codes:  0 '***' 0.001 '**' 0.01 '*' 0.05 '.' 0.1 ' ' 1
##
## Residual standard error: 0.003207 on 23 degrees of freedom
## Multiple R-squared:  0.9999, Adjusted R-squared:  0.9999
## F-statistic: 2.94e+05 on 1 and 23 DF,  p-value: < 2.2e-16
```

```r
### PANEL C
plot(H2S/1000 ~ NH4_init,
     col=brewer.pal(n=8,name="Dark2")[8],
     lwd=5, ylim=c(0,12), type="line",
     xlab="[NH4+] initial (uM)",
     ylab="[H2S] produced (mM)",
     cex.axis=2, cex.lab=2)
```

```
## Warning in plot.xy(xy, type, ...): Le type de graphique 'line' sera tronqué au
## premier caractère
```

```r
points(DATA$H2S_max~DATA$`[NH4]`,
       pch=c(16,15,17)[DATA$EXP],
       cex=3,lwd=2,
       col=brewer.pal(n=8, name="Dark2")[DATA$EXP])
arrows(DATA$`[NH4]`,
       DATA$H2S_max-DATA$H2S_SD,DATA$`[NH4]`,
       DATA$H2S_max+DATA$H2S_SD,code=0,
       col=brewer.pal(n=8, name="Dark2")[DATA$EXP],lwd=2  )

legend(1000,12,legend = c("Model", "Data"),
       lty = c(1,0), pch=c(NA, 21),
       cex=1.5, lwd=3,bty = "n",
       col="black")
```

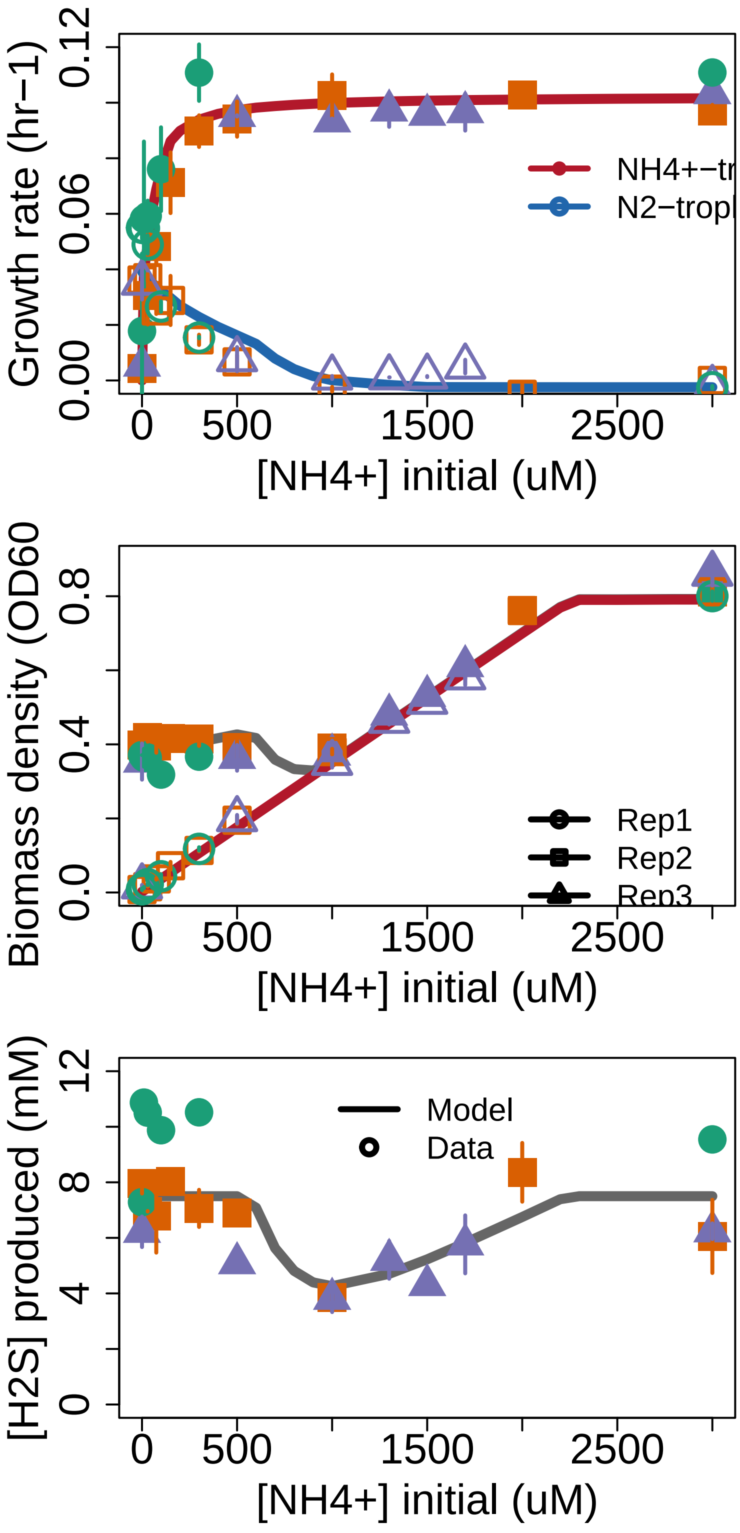


```
# legend(2000,3500,legend = c("Rep1", "Rep2","Rep3"),
# pch=c(16,15,17), col=brewer.pal(n=8, name="Dark2")[1:3],
# cex=1.5,bty = "n")
```

```r
#####Estimation of Ks_NH4 and Corrected biomass and growth rate ratio
nls_Ks=nls(tab$mu_NH4_app ~ mu_NH4/(1+Km_NH4/(tab$conc_NH4)),
           start=c(mu_NH4=0.1,Km_NH4=20), data=tab)
confint2(nls_Ks,  level = 0.95, method = "asymptotic")
```

```
##              2.5 %     97.5 %
## mu_NH4  0.1010813  0.1045978
## Km_NH4 28.2252504 36.1085865
```

```r
####Table of parameters
parameter=data.frame(Variable=as.character('Apparent Growth rate NH4'),
                     Value=as.double(max(tab$mu_NH4_app)),
                     SD=as.double(0))
parameter=rbind(parameter,
                c("Apparent Growth rate BNF",
                  max(tab$mu_BNF_app), 0))
parameter=rbind(parameter,
                c("Apparent growth rate yield ratio",
                  max(tab$mu_NH4_app)/max(tab$mu_BNF_app),
                  0))

parameter=rbind(parameter,
                c("Corrected Growth rate NH4",
                  max(tab$mu_NH4_true), 0))
parameter=rbind(parameter,
                c("Corrected Growth rate BNF",
                  max(tab$mu_BNF_true), 0))
parameter=rbind(parameter,
                c("Corrected growth rate yield ratio",
                  max(tab$mu_NH4_true)/max(tab$mu_BNF_true),
                  0))



parameter=rbind(parameter,
                c("Corrected Biomass yield NH4",
 (max(dat[[length(dat)]]$OD_NH4)+max(dat[[length(dat)]]$OD_dead)),
                  0))
parameter=rbind(parameter,
   c("Corrected Biomass yield BNF",
  (max(dat[[1]]$OD_cell_active)
   +dat[[1]]$OD_dead[which(dat[[1]]$OD_cell_active
                           ==max(dat[[1]]$OD_cell_active))]), 0))

parameter=rbind(parameter, c("Corrected Biomass yield ratio",
     (max(dat[[length(dat)]]$OD_NH4))/(max(dat[[1]]$OD_BNF)),
     0))

parameter=rbind(parameter,
                c("Apparent Biomass yield NH4",
                  max(dat[[length(dat)]]$OD_cell_active),
                  0))
```

```
parameter=rbind(parameter,
                c("Apparent Biomass yield BNF",
                  max(dat[[1]]$OD_cell_active),
                  0))
parameter=rbind(parameter,
                c("Corrected Biomass yield ratio",
  (max(dat[[length(dat)]]$OD_NH4)
   +max(dat[[length(dat)]]$OD_dead))/(max(dat[[1]]$OD_BNF)
                                   +max(dat[[1]]$OD_dead)),
                  0))

parameter
```

```
##                            Variable              Value SD
## 1           Apparent Growth rate NH4  0.101594658777455  0
## 2           Apparent Growth rate BNF 0.0391715257469798  0
## 3   Apparent growth rate yield ratio   2.59358441725463  0
## 4          Corrected Growth rate NH4  0.113060401472291  0
## 5          Corrected Growth rate BNF 0.0435229835630254  0
## 6  Corrected growth rate yield ratio     2.597717164968  0
## 7        Corrected Biomass yield NH4   1.15984264786316  0
## 8        Corrected Biomass yield BNF  0.399935679747306  0
## 9      Corrected Biomass yield ratio   2.10851645905504  0
## 10        Apparent Biomass yield NH4  0.792551000355311  0
## 11        Apparent Biomass yield BNF  0.376670352835881  0
## 12     Corrected Biomass yield ratio   2.23036547855288  0
```

```
# dev.off()
```

###FITTING ESTIMATE

```
###MESSQ for DATA

###OD_max
SE=0
MESQ=data.frame(NH4=as.double(),exp=as.double(),
                theo=as.double(), ERR=as.double())

for (i in 1:nrow(DATA)){

  MESQ[i,1]=DATA$`[NH4]`[i]
  MESQ[i,2]=DATA$ODMax[i]
  MESQ[i,3]=DvH$Tot_biomass[which(DvH$NH4_init==DATA$`[NH4]`[i])]
  MESQ[i,4]=(DATA$ODMax[i]
             -DvH$Tot_biomass[which(DvH$NH4_init==DATA$`[NH4]`[i])])


  SE=SE+((DATA$ODMax[i]-
            DvH$Tot_biomass[which(DvH$NH4_init
                                  ==DATA$`[NH4]`[i])])/DvH$Tot_biomass[which(DvH$NH4_init==DATA$`[NH4]`

}
```

```
plot(MESQ$exp~MESQ$theo, ylim=c(0,1), xlim=c(0,1),
     main="Predict vs Measured Maximal Biomass Yield",
     xlab="Measured values", ylab="Modeled values")
# arrows(MESQ$theo,MESQ$exp-DATA$OD_SD,MESQ$theo,
#        MESQ$exp+DATA$OD_SD,code=0, lwd=1.5 )

abline(0,1, col="red")
```

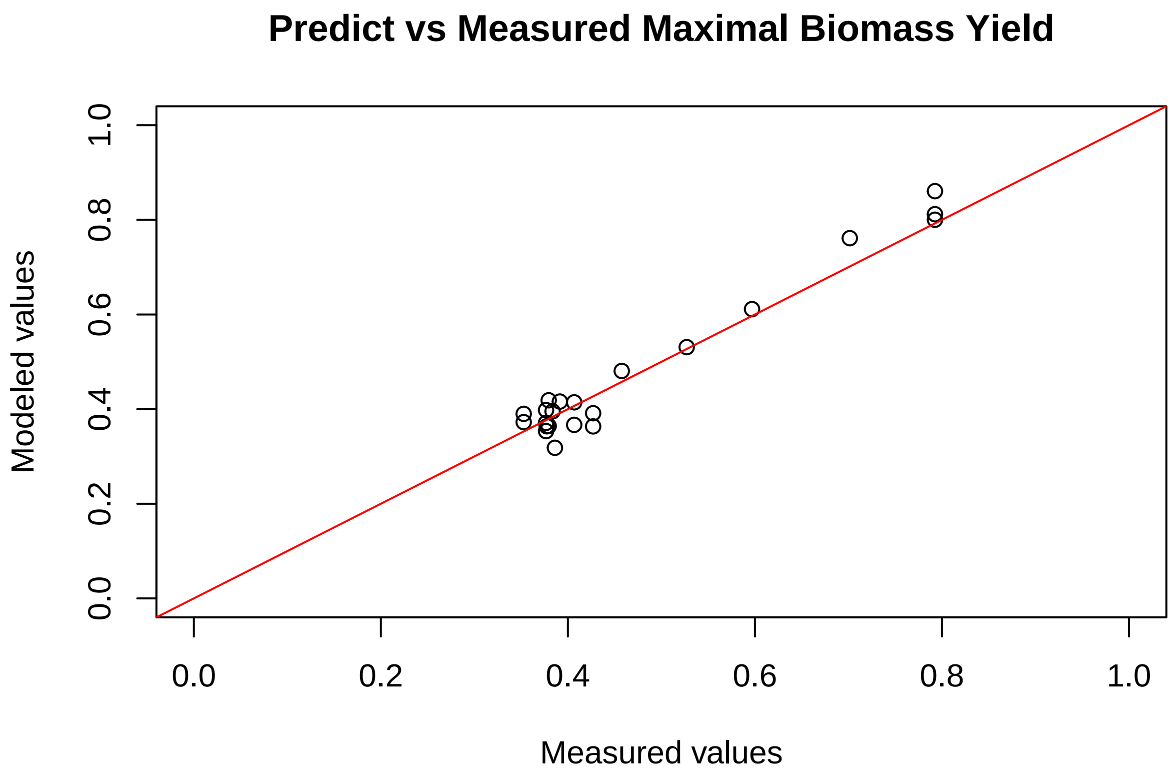


```
print("OD_MAX")
```

```
## [1] "OD_MAX"
```

```
summary(lm((MESQ$exp)~MESQ$theo))
```

```
##
## Call:
## lm(formula = (MESQ$exp) ~ MESQ$theo)
##
## Residuals:
##       Min        1Q    Median        3Q       Max
## -0.063446 -0.017250 -0.000964  0.027222  0.044907
##
## Coefficients:
```

```
##              Estimate Std. Error t value Pr(>|t|)
## (Intercept) -0.04184    0.02319  -1.804   0.0863 .
## MESQ$theo    1.09690    0.04651  23.586 4.54e-16 ***
## ---
## Signif. codes:  0 '***' 0.001 '**' 0.01 '*' 0.05 '.' 0.1 ' ' 1
##
## Residual standard error: 0.03274 on 20 degrees of freedom
## Multiple R-squared:  0.9653, Adjusted R-squared:  0.9636
## F-statistic: 556.3 on 1 and 20 DF,  p-value: 4.536e-16
```

```
##Is prediction different from measured
##(is measured~prediction a 1:1 line)

plot(MESQ$exp-MESQ$theo~MESQ$theo,
     main="Bias",
     xlab="Measured values", ylab="Residual")
abline(h=0, lw=2)
lines(MESQ$theo,predict(lm((MESQ$exp-MESQ$theo)~MESQ$theo)), col="red", lw=2)
```

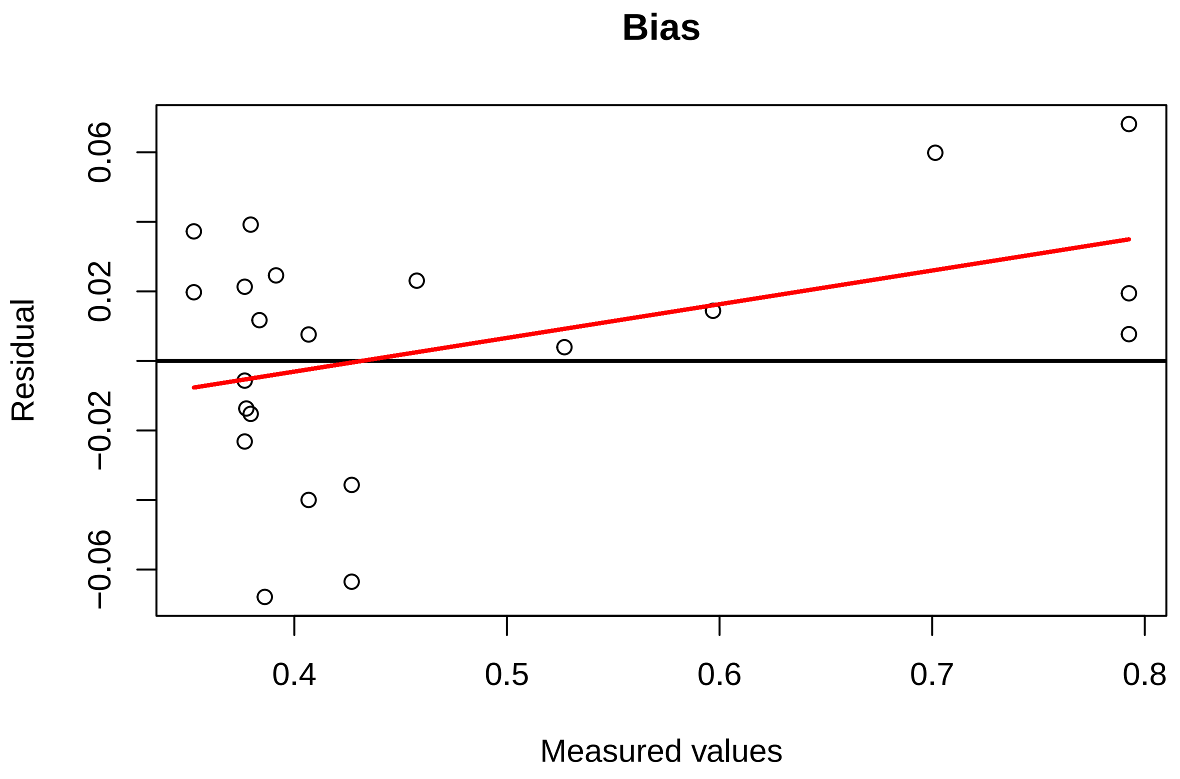


```
summary(lm((MESQ$exp-MESQ$theo)~MESQ$theo))
```

```
##
## Call:
## lm(formula = (MESQ$exp - MESQ$theo) ~ MESQ$theo)
##
```

```
## Residuals:
##       Min        1Q    Median        3Q       Max
## -0.063446 -0.017250 -0.000964  0.027222  0.044907
##
## Coefficients:
##             Estimate Std. Error t value Pr(>|t|)
## (Intercept) -0.04184    0.02319  -1.804   0.0863 .
## MESQ$theo    0.09690    0.04651   2.084   0.0502 .
## ---
## Signif. codes:  0 '***' 0.001 '**' 0.01 '*' 0.05 '.' 0.1 ' ' 1
##
## Residual standard error: 0.03274 on 20 degrees of freedom
## Multiple R-squared:  0.1783, Adjusted R-squared:  0.1373
## F-statistic: 4.341 on 1 and 20 DF,  p-value: 0.05024
```

```
###OD_NH4
# SE=0
MESQ2=data.frame(NH4=as.double(),exp=as.double(),
                 theo=as.double(), ERR=as.double())

for (i in 1:nrow(DATA)){

  MESQ2[i,1]=DATA$`[NH4]`[i]
  MESQ2[i,2]=DATA$OD_NH4[i]
  MESQ2[i,3]=DvH$NH4_biomass[which(DvH$NH4_init==DATA$`[NH4]`[i])]
  MESQ2[i,4]=(DATA$OD_NH4[i]
              -DvH$NH4_biomass[which(DvH$NH4_init==DATA$`[NH4]`[i])])


  #
}

plot(MESQ2$exp~MESQ2$theo, ylim=c(0,1), xlim=c(0,1),
     main="Plateau NH4 biomass")
# arrows(MESQ2$theo,MESQ2$exp-DATA$SD_OD_NH4,MESQ2$theo,
#        MESQ2$exp+DATA$SD_OD_NH4,code=0, lwd=1.5 )
abline(0,1, col="red")
```

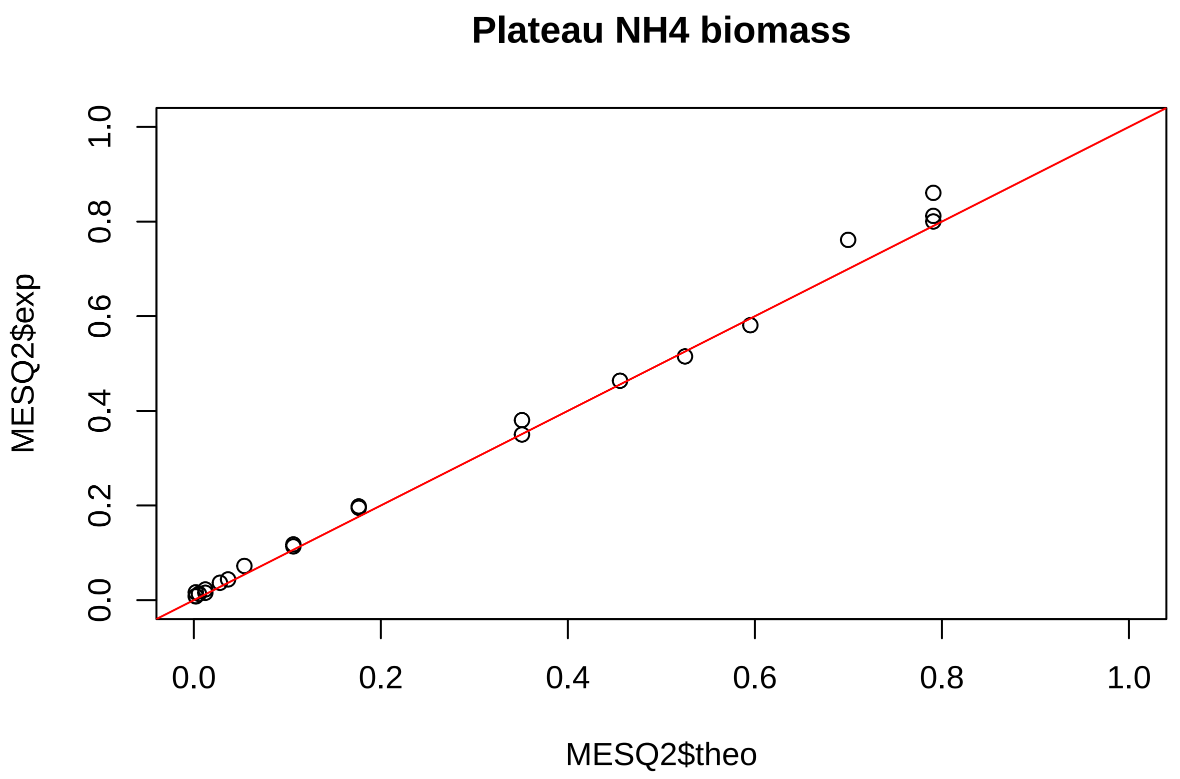


```
print("OD_AMMONIUM")
```

```
## [1] "OD_AMMONIUM"
```

```
summary(lm((MESQ2$exp)~MESQ2$theo))
```

```
##
## Call:
## lm(formula = (MESQ2$exp) ~ MESQ2$theo)
##
## Residuals:
##       Min        1Q    Median        3Q       Max
## -0.036389 -0.005567 -0.000406  0.006945  0.042752
##
## Coefficients:
##             Estimate Std. Error t value Pr(>|t|)
## (Intercept) 0.007467   0.005379   1.388     0.18
## MESQ2$theo  1.024844   0.013410  76.423   <2e-16 ***
## ---
## Signif. codes:  0 '***' 0.001 '**' 0.01 '*' 0.05 '.' 0.1 ' ' 1
##
## Residual standard error: 0.01831 on 20 degrees of freedom
## Multiple R-squared:  0.9966, Adjusted R-squared:  0.9964
## F-statistic:  5841 on 1 and 20 DF,  p-value: < 2.2e-16
```

```r
##is prediction different from measured (is measured~prediction a 1:1 line)
plot((MESQ2$exp-MESQ2$theo)~MESQ2$theo,
     main="Bias",
     xlab="Measured values", ylab="Residual")
abline(h=0, lw=2)
lines(MESQ2$theo,predict(
  lm((MESQ2$exp-MESQ2$theo)~MESQ2$theo)), col="red", lw=2)
```

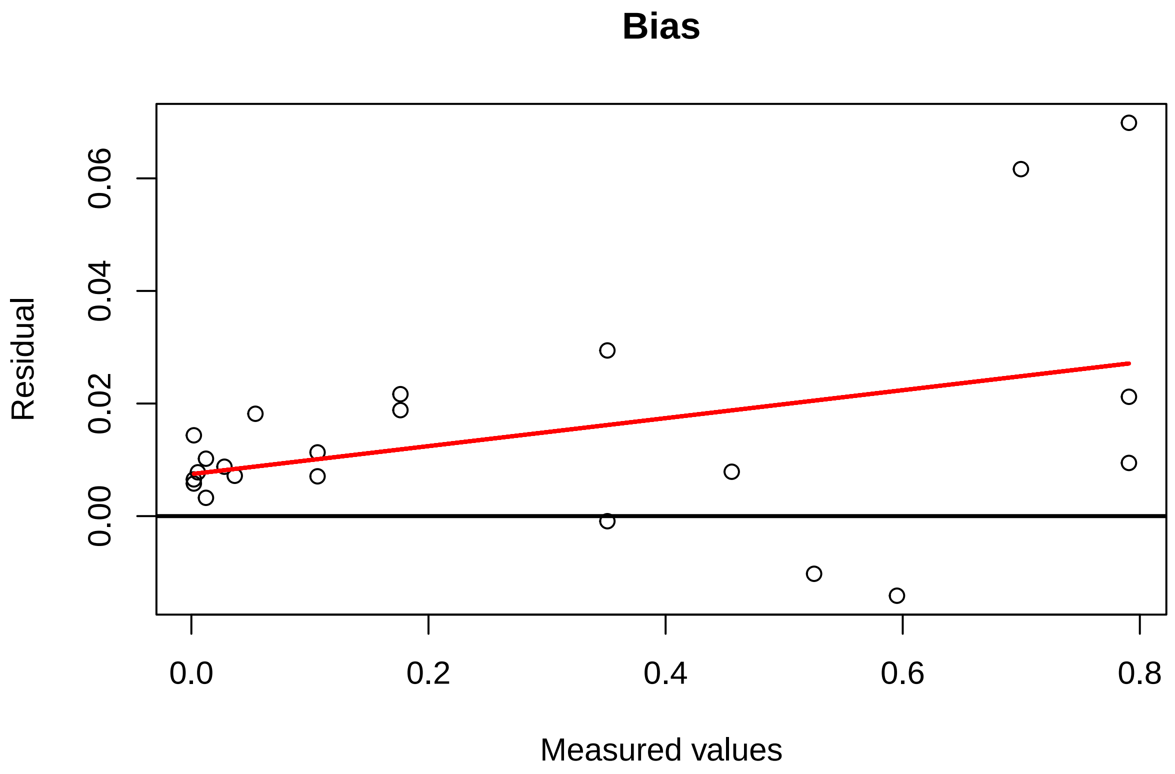


```r
summary(lm((MESQ2$exp-MESQ2$theo)~MESQ2$theo))
```

```
##
## Call:
## lm(formula = (MESQ2$exp - MESQ2$theo) ~ MESQ2$theo)
##
## Residuals:
##       Min        1Q    Median        3Q       Max
## -0.036389 -0.005567 -0.000406  0.006945  0.042752
##
## Coefficients:
##             Estimate Std. Error t value Pr(>|t|)
## (Intercept) 0.007467   0.005379   1.388   0.1804
## MESQ2$theo  0.024844   0.013410   1.853   0.0788 .
## ---
## Signif. codes:  0 '***' 0.001 '**' 0.01 '*' 0.05 '.' 0.1 ' ' 1
##
```

```
## Residual standard error: 0.01831 on 20 degrees of freedom
## Multiple R-squared:  0.1465, Adjusted R-squared:  0.1038
## F-statistic: 3.432 on 1 and 20 DF,  p-value: 0.07875
```

```
###H2S
# SE=0
MESQ3=data.frame(NH4=as.double(),
                 exp=as.double(),
                 theo=as.double(),
                 ERR=as.double())

for (i in 1:nrow(DATA)){

  MESQ3[i,1]=DATA$`[NH4]`[i]
  MESQ3[i,2]=DATA$H2S_max[i]*1000
  MESQ3[i,3]=DvH$H2S[which(DvH$NH4_init==DATA$`[NH4]`[i])]
  MESQ3[i,4]=(DATA$H2S_max[i]
              -DvH$H2S[which(DvH$NH4_init==DATA$`[NH4]`[i])])



}

plot(MESQ3$exp~MESQ3$theo, ylim=c(0,12000),
     xlim=c(0,12000), main="H2S production")
# arrows(MESQ3$theo,MESQ3$exp-DATA$H2S_SD*1000,
#        MESQ3$theo,MESQ3$exp+DATA$H2S_SD*1000,code=0, lwd=1.5 )
abline(0,1, col="red")
```

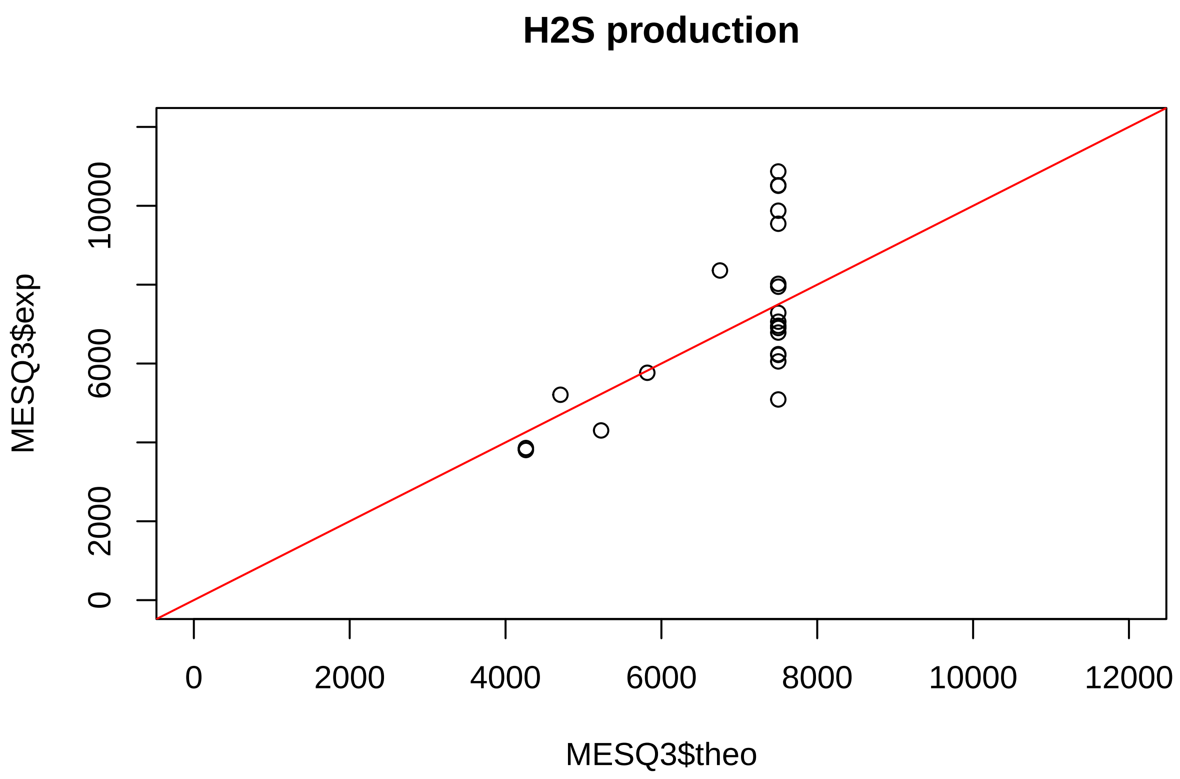


```
print("H2S")
```

```
## [1] "H2S"
```

```
summary(lm((MESQ3$exp)~MESQ3$theo))
```

```
##
## Call:
## lm(formula = (MESQ3$exp) ~ MESQ3$theo)
##
## Residuals:
##     Min      1Q  Median      3Q     Max
## -2831.0 -1004.7  -135.9  1190.0  2950.4
##
## Coefficients:
##              Estimate Std. Error t value Pr(>|t|)
## (Intercept) -1231.359   2129.535  -0.578 0.569564
## MESQ3$theo      1.220      0.306   3.987 0.000726 ***
## ---
## Signif. codes:  0 '***' 0.001 '**' 0.01 '*' 0.05 '.' 0.1 ' ' 1
##
## Residual standard error: 1633 on 20 degrees of freedom
## Multiple R-squared:  0.4428, Adjusted R-squared:  0.415
## F-statistic: 15.89 on 1 and 20 DF,  p-value: 0.0007255
```

```r
##is prediction different from measured (is measured~prediction a 1:1 line)
plot((MESQ3$exp-MESQ3$theo)~MESQ3$theo,
     main="Bias",
     xlab="Measured values", ylab="Residual")
abline(h=0, lw=2)
lines(MESQ3$theo,
      predict(lm((MESQ3$exp-MESQ3$theo)~MESQ3$theo)), col="red", lw=2)
```

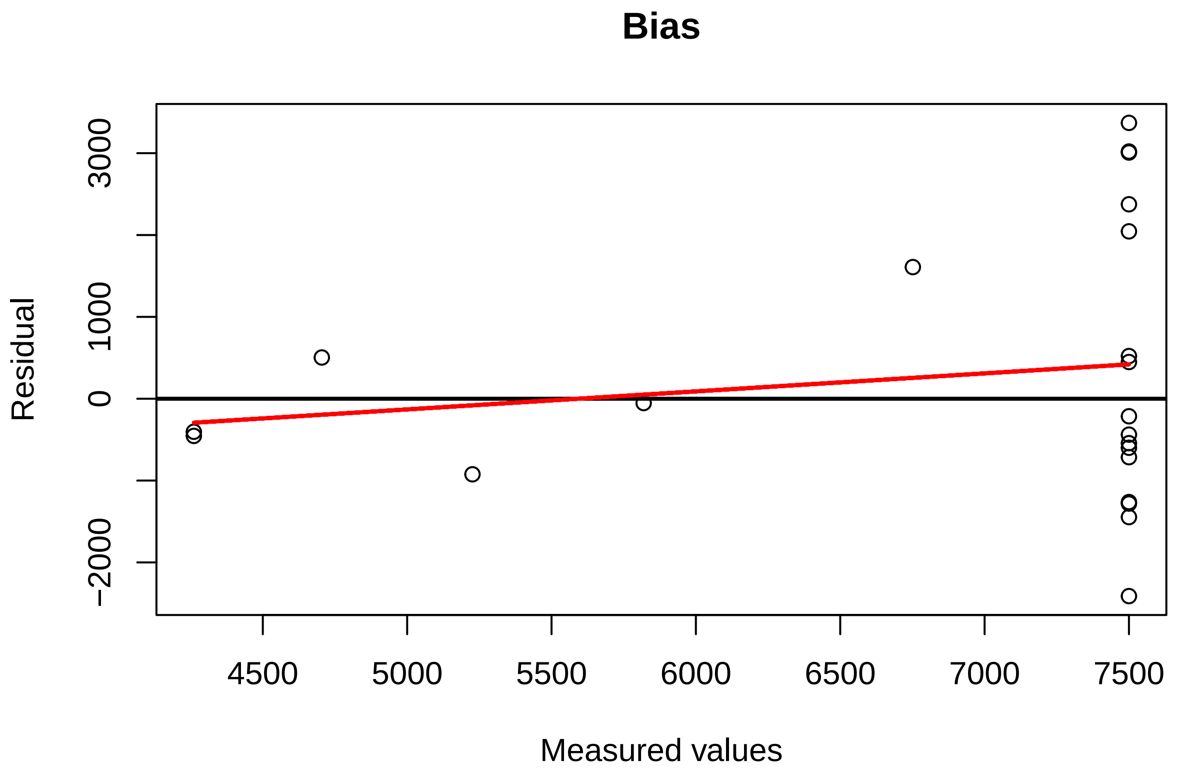


```r
summary(lm((MESQ3$exp-MESQ3$theo)~MESQ3$theo))
```

```
##
## Call:
## lm(formula = (MESQ3$exp - MESQ3$theo) ~ MESQ3$theo)
##
## Residuals:
##     Min      1Q  Median      3Q     Max
## -2831.0 -1004.7  -135.9  1190.0  2950.4
##
## Coefficients:
##              Estimate Std. Error t value Pr(>|t|)
## (Intercept) -1231.3586  2129.5352  -0.578     0.57
## MESQ3$theo      0.2201     0.3060   0.719     0.48
##
## Residual standard error: 1633 on 20 degrees of freedom
## Multiple R-squared:  0.02522,    Adjusted R-squared:  -0.02352
```

```
## F-statistic: 0.5174 on 1 and 20 DF,  p-value: 0.4803
```

```
###Growth rate BNF
# SE=0
MESQ4=data.frame(NH4=as.double(),
                 exp=as.double(),
                 theo=as.double(),
                 ERR=as.double())

for (i in 1:nrow(DATA)){

  MESQ4[i,1]=DATA$`[NH4]`[i]
  MESQ4[i,2]=DATA$GR_BNF[i]
  MESQ4[i,3]=tab$mu_BNF_app[which(tab$conc_NH4==DATA$`[NH4]`[i])]
  MESQ4[i,4]=(DATA$GR_BNF[i]
              -tab$mu_BNF_app[which(tab$conc_NH4==DATA$`[NH4]`[i])])


}

plot(MESQ4$exp~MESQ4$theo, main="Growth rate during BNF")
# arrows(MESQ4$theo,MESQ4$exp-DATA$SD_GR_BNF,
#         MESQ4$theo,MESQ4$exp+DATA$SD_GR_BNF,code=0, lwd=1.5 )

abline(0,1, col="red")
```

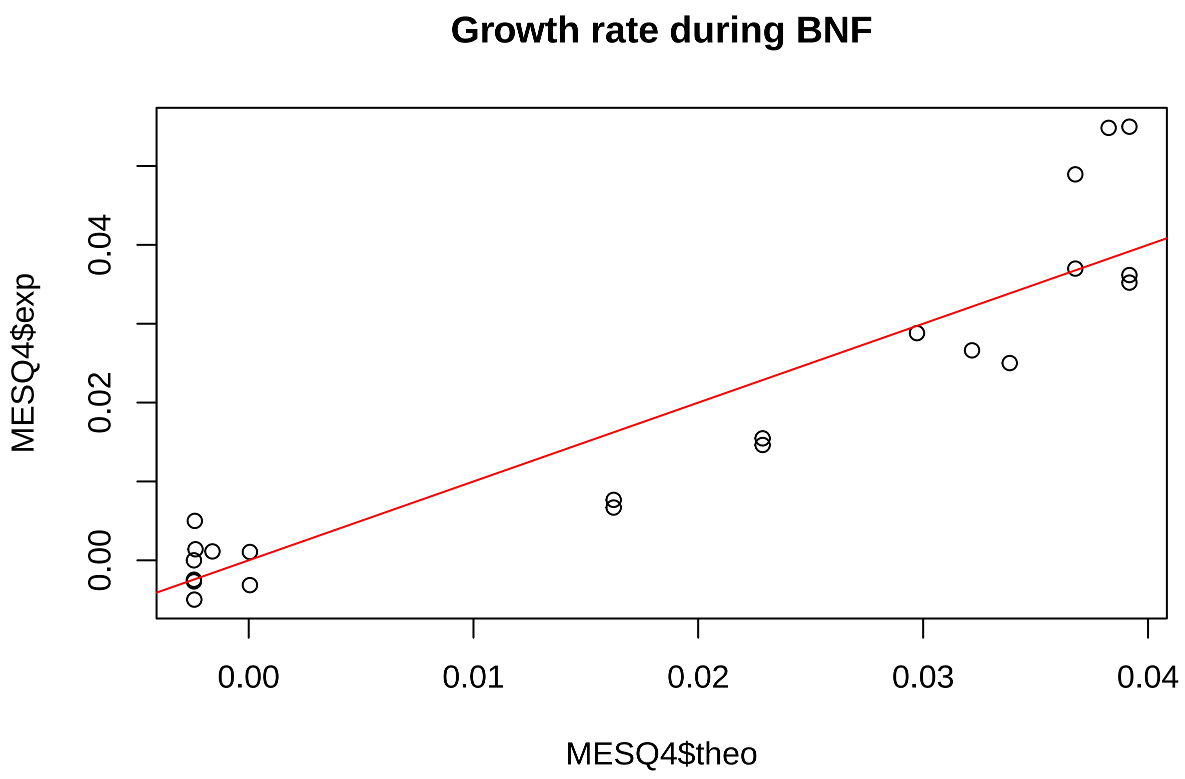

```r
print("GROWTH RATE BNF")
```

```
## [1] "GROWTH RATE BNF"
```

```r
summary(lm((MESQ4$exp)~MESQ4$theo))
```

```
##
## Call:
## lm(formula = (MESQ4$exp) ~ MESQ4$theo)
##
## Residuals:
##        Min         1Q     Median         3Q        Max
## -0.0094939 -0.0057675 -0.0009362  0.0033865  0.0158049
##
## Coefficients:
##               Estimate Std. Error t value Pr(>|t|)
## (Intercept) -0.0006592  0.0023378  -0.282    0.781
## MESQ4$theo   1.0375458  0.0945713  10.971 6.51e-10 ***
## ---
## Signif. codes:  0 '***' 0.001 '**' 0.01 '*' 0.05 '.' 0.1 ' ' 1
##
## Residual standard error: 0.007698 on 20 degrees of freedom
## Multiple R-squared:  0.8575, Adjusted R-squared:  0.8504
## F-statistic: 120.4 on 1 and 20 DF,  p-value: 6.515e-10
```

```r
##is prediction different from measured (is measured~prediction a 1:1 line)
plot((MESQ4$exp-MESQ4$theo)~MESQ4$theo,
     main="Bias",
     xlab="Measured values", ylab="Residual")
abline(h=0, lw=2)
lines(MESQ4$theo,predict(
  lm((MESQ4$exp-MESQ4$theo)~MESQ4$theo)), col="red", lw=2)
```

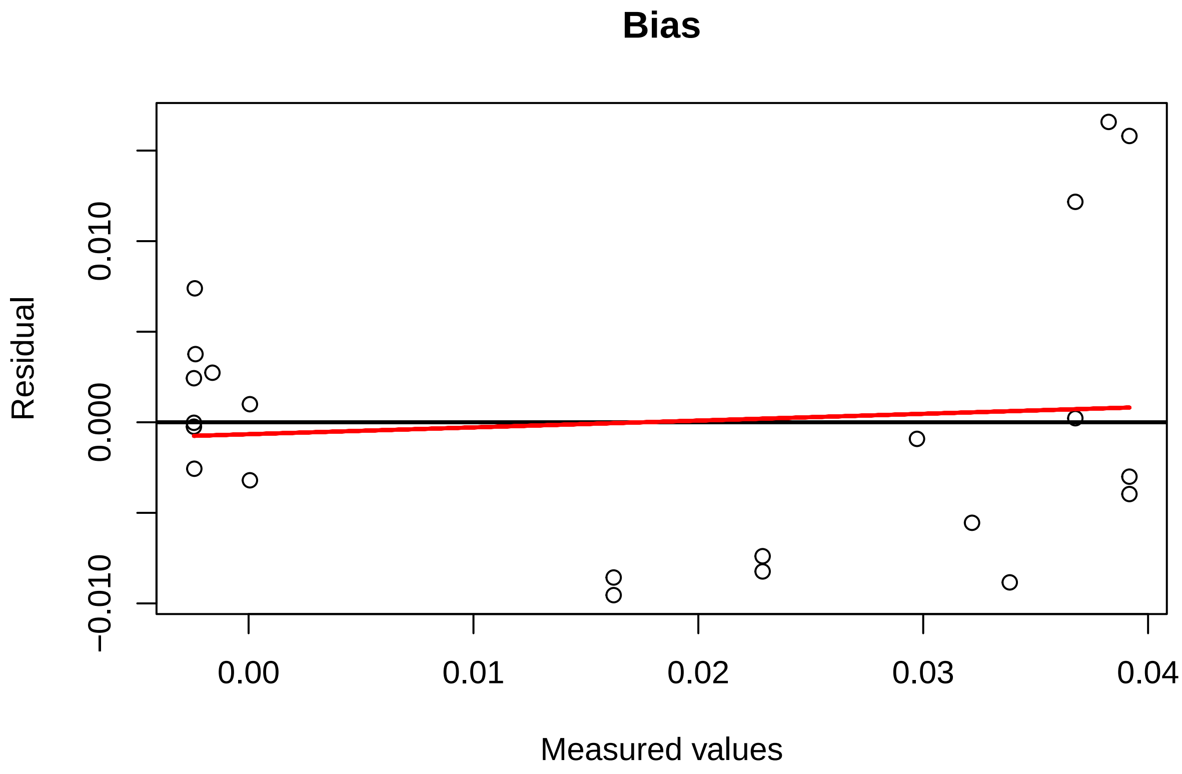


```
summary(lm((MESQ4$exp-MESQ4$theo)~MESQ4$theo))
```

```
##
## Call:
## lm(formula = (MESQ4$exp - MESQ4$theo) ~ MESQ4$theo)
##
## Residuals:
##        Min         1Q     Median         3Q        Max
## -0.0094939 -0.0057675 -0.0009362  0.0033865  0.0158049
##
## Coefficients:
##               Estimate Std. Error t value Pr(>|t|)
## (Intercept) -0.0006592  0.0023378  -0.282    0.781
## MESQ4$theo   0.0375458  0.0945713   0.397    0.696
##
## Residual standard error: 0.007698 on 20 degrees of freedom
## Multiple R-squared:  0.007819,   Adjusted R-squared:  -0.04179
## F-statistic: 0.1576 on 1 and 20 DF,  p-value: 0.6956
```

```
###Growth rate NH4
# SE=0
MESQ5=data.frame(NH4=as.double(),exp=as.double(),
                 theo=as.double(), ERR=as.double())

for (i in 1:nrow(DATA)){
```

```r
  MESQ5[i,1]=DATA$`[NH4]`[i]
  MESQ5[i,2]=DATA$GR_NH4[i]
  MESQ5[i,3]=tab$mu_NH4_app[which(tab$conc_NH4==DATA$`[NH4]`[i])]
  MESQ5[i,4]=(DATA$GR_NH4[i]
              -tab$mu_NH4_app[which(tab$conc_NH4==DATA$`[NH4]`[i])])



}

plot(MESQ5$exp~MESQ5$theo,
     main="Growth rate during Ammoniotrophy")
# arrows(MESQ5$theo,MESQ5$exp-DATA$SD_GR_NH4,
#         MESQ5$theo,MESQ5$exp+DATA$SD_GR_NH4,code=0,lwd=1.5 )

abline(0,1, col="red")
```

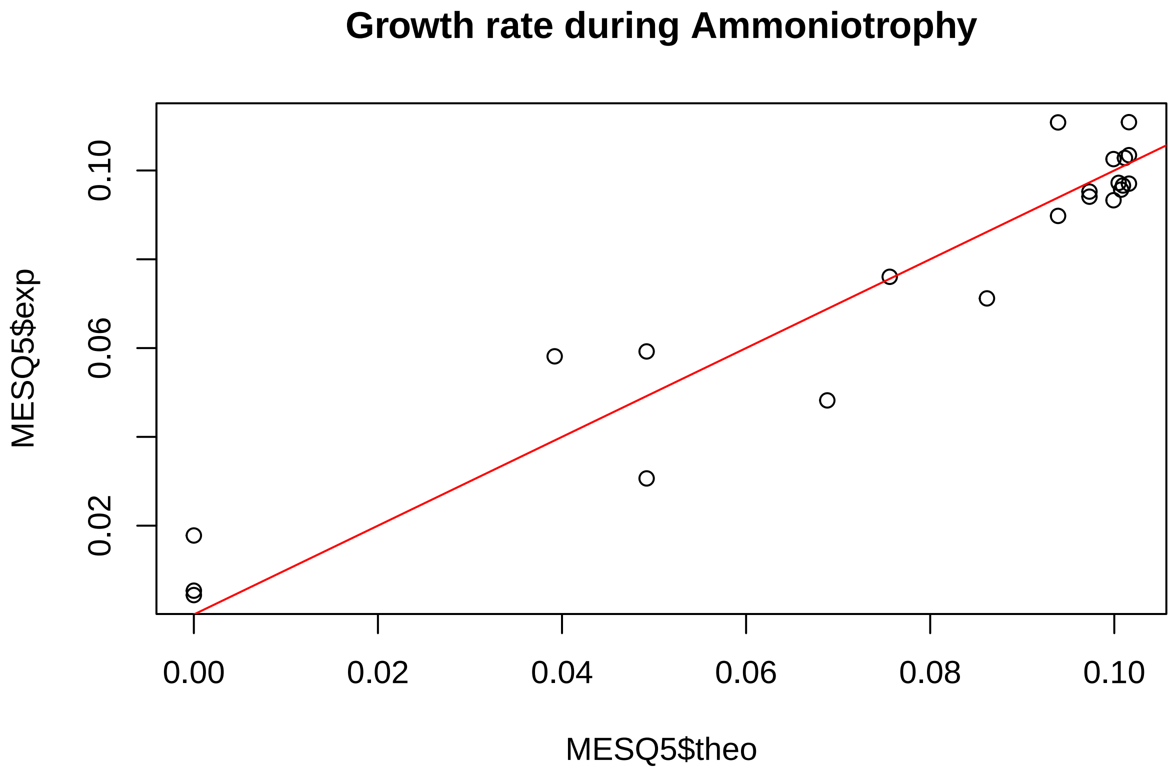


```r
print("GROWTH_NH4")
```

```
## [1] "GROWTH_NH4"
```

```r
summary(lm((MESQ5$exp)~MESQ5$theo))
```

```
##
```

```
## Call:
## lm(formula = (MESQ5$exp) ~ MESQ5$theo)
##
## Residuals:
##       Min        1Q    Median        3Q       Max
## -0.021330 -0.002637 -0.001058  0.004769  0.018627
##
## Coefficients:
##             Estimate Std. Error t value Pr(>|t|)
## (Intercept) 0.007367   0.005152    1.43    0.168
## MESQ5$theo  0.903490   0.061899   14.60 3.98e-12 ***
## ---
## Signif. codes:  0 '***' 0.001 '**' 0.01 '*' 0.05 '.' 0.1 ' ' 1
##
## Residual standard error: 0.01024 on 20 degrees of freedom
## Multiple R-squared:  0.9142, Adjusted R-squared:  0.9099
## F-statistic: 213.1 on 1 and 20 DF,  p-value: 3.976e-12
```

```
##is prediction different from measured (is measured~prediction a 1:1 line)
 plot((MESQ5$exp-MESQ5$theo)~MESQ5$theo,
     main="Bias",
     xlab="Measured values", ylab="Residual")
abline(h=0, lw=2)
lines(MESQ5$theo,predict(
  lm((MESQ5$exp-MESQ5$theo)~MESQ5$theo)), col="red", lw=2)
```

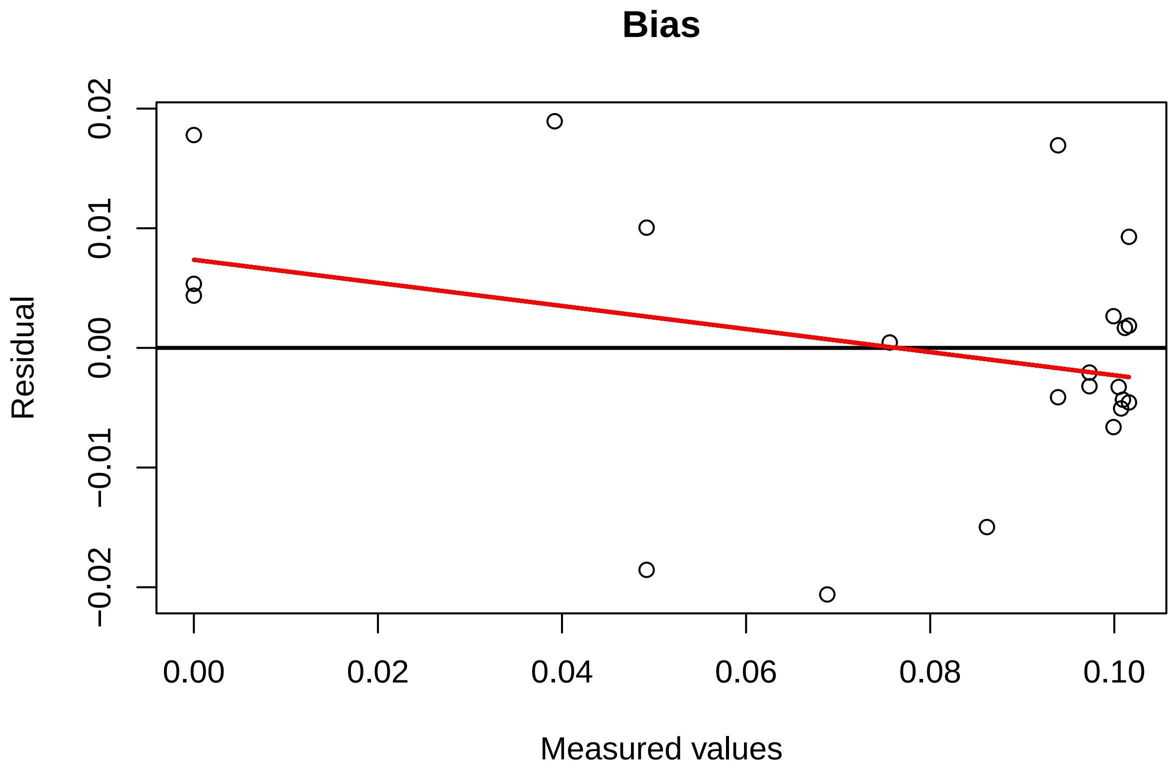

```
summary(lm((MESQ5$exp-MESQ5$theo)~MESQ5$theo))
```

```
##
## Call:
## lm(formula = (MESQ5$exp - MESQ5$theo) ~ MESQ5$theo)
##
## Residuals:
##       Min        1Q    Median        3Q       Max
## -0.021330 -0.002637 -0.001058  0.004769  0.018627
##
## Coefficients:
##              Estimate Std. Error t value Pr(>|t|)
## (Intercept)  0.007367   0.005152   1.430    0.168
## MESQ5$theo  -0.096510   0.061899  -1.559    0.135
##
## Residual standard error: 0.01024 on 20 degrees of freedom
## Multiple R-squared:  0.1084, Adjusted R-squared:  0.06379
## F-statistic: 2.431 on 1 and 20 DF,  p-value: 0.1346
```

####GROWTH CURVES VALIDATION

```
# pdf("2023_Figure3.pdf", height=11, width=8)
# par(mfrow=c(5,3), mar=c(5,5,2,2))

 par(mfrow=c(2,3), mar=c(5,5,2,2))

conc=c(0,10, 30, 75, 100, 150, 300, 500,
       1000,1300, 1500,1700,2000, 3000)

index_conc=list()

index_conc[[2]]=cbind(3000,3000,3000,
                      2000,2000,2000,1000,1000,1000,
                      500,500,500, 300,300,300,
                      150,150,150, 75,75,75,
                      30,30,30, 0,0,0)

index_conc[[1]]=cbind(0,0,0,0,0,0,0,0,0,
                      500,500,1000,1000,1000,
                      1000,1000,1000,1000,1000,
                      1000,1500,1000,1300,1300,1300,
                      1700,1700,1700,3000,3000,3000)

index_conc[[3]]=cbind(3000,3000,3000,3000,
                      300,300,300,300,
                      100,100,100,100,
                      30,30,30,30,
                      10,10,10,10,0)

dat=list(data.frame())
a="[NH4+]init ="
```

```r
GR=data.frame(TIME=as.double(),
              exp=as.double(),
              theo=as.double(),
              ERR=as.double())

for (i in 1:length(conc)){
  GR_2=data.frame(TIME=as.double(),
              exp=as.double(),
              theo=as.double(),
              ERR=as.double())

  media$conc_NH4_ext=conc[i]
  dat[[i]]=Culture_lim(media,
                       metabolism,
                       method="addition")


  plot(OD_cell_active~Time,
       data=dat[[i]], main=paste(a,conc[i],""),
       ylim=c(0,0.9), cex.axis=2,
       type="l", lwd=5, ylab="Biomass density (OD600)")

  for (l in 1:3){


   g=15
   for (k in 1:length(index_conc[[l]])){

    if(index_conc[[l]][k]==conc[i]){

      points(DATA_GROWTH[[l]][,k+1]~DATA_GROWTH[[l]]$TIME,
             cex=2, pch=g, col=alpha(col[l],0.5) )

      GR_1=data.frame(TIME=as.double(),exp=as.double(),
                      theo=as.double(), REP=as.double())
      g=g+1

      for (j in 1:nrow(DATA_GROWTH[[l]])){
        if(!(length((which(dat[[i]]$Time
                   ==round(DATA_GROWTH[[l]]$TIME[j],0))))==0) & !(is.na(DATA_GROWTH[[l]][j,k+1]]


          GR_1[j,1]=DATA_GROWTH[[l]]$TIME[j]
          GR_1[j,2]=DATA_GROWTH[[l]][j,k+1]

           GR_1[j,3]=dat[[i]]$OD_cell_active[which(dat[[i]]$Time
                                 ==round((DATA_GROWTH[[l]]$TIME[j]),0))]

          GR_1[j,4]=l


        }
```

```
      }

      GR=rbind.data.frame(GR, GR_1)
      GR_2=rbind.data.frame(GR_2, GR_1)
      GR_1=0

     }

    }

  }


  print(conc[i])
  print(summary(lm((GR_2$exp)~GR_2$theo)))
  print(summary(lm((GR_2$exp-GR_2$theo)~GR_2$theo)))
  GR=rbind(GR,GR_2)
  GR_2=0

  ###UNCOMMENT FOR INDIVIDUAL PLOT FITTING

  # plot(GR$exp~GR$theo, main="Biomass over time", col="red")
  # # points(GR_2$exp~GR_2$theo, col="blue")
  # # points(GR_3$exp~GR_3$theo, col="green")
  # abline(0,1, col="red")

  #print("All graph")
  plot(GR$exp~GR$theo, main="Biomass over time", col="red")
  abline(0,1, col="black", lw=2)
  summary(lm((GR$exp)~GR$theo))
  # ##is prediction different from measured (is measured~prediction a 1:1 line)
  plot(GR$exp-GR$theo~GR$theo, main="Bias", col="red")
  abline(h=0, col="black", lw=2)
   summary(lm((GR$exp-GR$theo)~GR$theo))


}
```

```
## [1] 0
##
## Call:
## lm(formula = (GR_2$exp) ~ GR_2$theo)
##
## Residuals:
##       Min        1Q    Median        3Q       Max
## -0.287874 -0.028189 -0.007887  0.072715  0.188553
##
## Coefficients:
##             Estimate Std. Error t value Pr(>|t|)
## (Intercept)  0.02137    0.00825   2.591     0.01 *
## GR_2$theo    0.73418    0.03653  20.100   <2e-16 ***
## ---
## Signif. codes:  0 '***' 0.001 '**' 0.01 '*' 0.05 '.' 0.1 ' ' 1
```

```
##
## Residual standard error: 0.09371 on 301 degrees of freedom
##   (15 observations effacées parce que manquantes)
## Multiple R-squared:  0.573,  Adjusted R-squared:  0.5716
## F-statistic:   404 on 1 and 301 DF,  p-value: < 2.2e-16
##
##
## Call:
## lm(formula = (GR_2$exp - GR_2$theo) ~ GR_2$theo)
##
## Residuals:
##       Min        1Q    Median        3Q       Max
## -0.287874 -0.028189 -0.007887  0.072715  0.188553
##
## Coefficients:
##             Estimate Std. Error t value Pr(>|t|)
## (Intercept)  0.02137    0.00825   2.591     0.01 *
## GR_2$theo   -0.26582    0.03653  -7.277 2.98e-12 ***
## ---
## Signif. codes:  0 '***' 0.001 '**' 0.01 '*' 0.05 '.' 0.1 ' ' 1
##
## Residual standard error: 0.09371 on 301 degrees of freedom
##   (15 observations effacées parce que manquantes)
## Multiple R-squared:  0.1496, Adjusted R-squared:  0.1468
## F-statistic: 52.96 on 1 and 301 DF,  p-value: 2.976e-12

## [1] 10
##
## Call:
## lm(formula = (GR_2$exp) ~ GR_2$theo)
##
## Residuals:
##      Min       1Q   Median       3Q      Max
## -0.04360 -0.02958 -0.01960  0.02251  0.13657
##
## Coefficients:
##             Estimate Std. Error t value Pr(>|t|)
## (Intercept)  0.03164    0.00541   5.849 8.08e-08 ***
## GR_2$theo    0.95268    0.03588  26.551  < 2e-16 ***
## ---
## Signif. codes:  0 '***' 0.001 '**' 0.01 '*' 0.05 '.' 0.1 ' ' 1
##
## Residual standard error: 0.04236 on 89 degrees of freedom
##   (33 observations effacées parce que manquantes)
## Multiple R-squared:  0.8879, Adjusted R-squared:  0.8866
## F-statistic: 704.9 on 1 and 89 DF,  p-value: < 2.2e-16
##
##
## Call:
## lm(formula = (GR_2$exp - GR_2$theo) ~ GR_2$theo)
##
## Residuals:
##      Min       1Q   Median       3Q      Max
## -0.04360 -0.02958 -0.01960  0.02251  0.13657
```

```
##
## Coefficients:
##              Estimate Std. Error t value Pr(>|t|)
## (Intercept)  0.03164    0.00541   5.849 8.08e-08 ***
## GR_2$theo   -0.04732    0.03588  -1.319    0.191
## ---
## Signif. codes:  0 '***' 0.001 '**' 0.01 '*' 0.05 '.' 0.1 ' ' 1
##
## Residual standard error: 0.04236 on 89 degrees of freedom
##   (33 observations effacées parce que manquantes)
## Multiple R-squared:  0.01917,    Adjusted R-squared:  0.00815
## F-statistic:  1.74 on 1 and 89 DF,  p-value: 0.1906
```

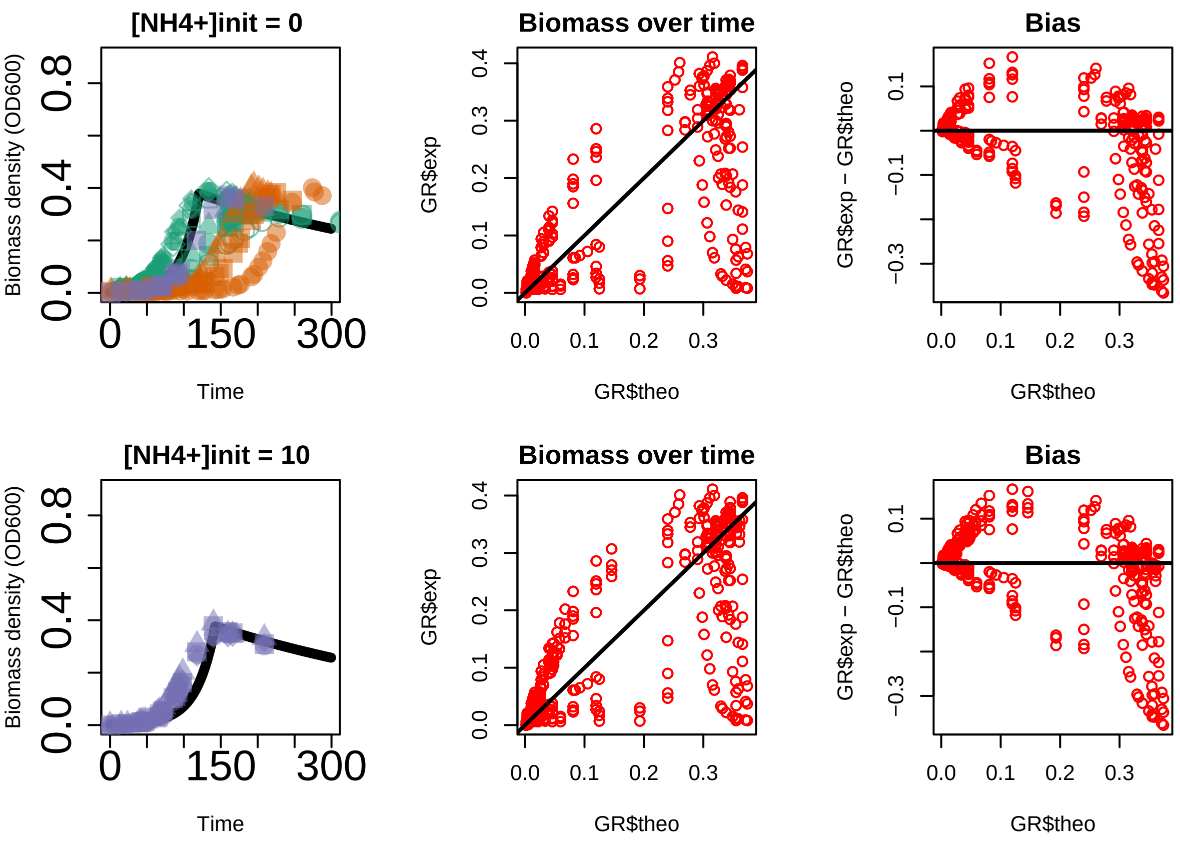


```
## [1] 30
##
## Call:
## lm(formula = (GR_2$exp) ~ GR_2$theo)
##
## Residuals:
##      Min       1Q   Median       3Q      Max
## -0.29966 -0.03653 -0.02698  0.05160  0.20683
##
## Coefficients:
##              Estimate Std. Error t value Pr(>|t|)
## (Intercept) 0.039518   0.007914   4.993 1.41e-06 ***
```

```
## GR_2$theo   0.827143   0.041741  19.816  < 2e-16 ***
## ---
## Signif. codes:  0 '***' 0.001 '**' 0.01 '*' 0.05 '.' 0.1 ' ' 1
##
## Residual standard error: 0.07988 on 178 degrees of freedom
##   (32 observations effacées parce que manquantes)
## Multiple R-squared:  0.6881, Adjusted R-squared:  0.6863
## F-statistic: 392.7 on 1 and 178 DF,  p-value: < 2.2e-16
##
##
## Call:
## lm(formula = (GR_2$exp - GR_2$theo) ~ GR_2$theo)
##
## Residuals:
##      Min       1Q   Median       3Q      Max
## -0.29966 -0.03653 -0.02698  0.05160  0.20683
##
## Coefficients:
##              Estimate Std. Error t value Pr(>|t|)
## (Intercept)  0.039518   0.007914   4.993 1.41e-06 ***
## GR_2$theo   -0.172857   0.041741  -4.141 5.33e-05 ***
## ---
## Signif. codes:  0 '***' 0.001 '**' 0.01 '*' 0.05 '.' 0.1 ' ' 1
##
## Residual standard error: 0.07988 on 178 degrees of freedom
##   (32 observations effacées parce que manquantes)
## Multiple R-squared:  0.08788,    Adjusted R-squared:  0.08276
## F-statistic: 17.15 on 1 and 178 DF,  p-value: 5.328e-05
```

```
## [1] 75
##
## Call:
## lm(formula = (GR_2$exp) ~ GR_2$theo)
##
## Residuals:
##      Min       1Q   Median       3Q      Max
## -0.20155 -0.03716  0.00006  0.01245  0.20122
##
## Coefficients:
##             Estimate Std. Error t value Pr(>|t|)
## (Intercept) 0.005418   0.014267    0.38    0.705
## GR_2$theo   0.697120   0.061445   11.35   <2e-16 ***
## ---
## Signif. codes:  0 '***' 0.001 '**' 0.01 '*' 0.05 '.' 0.1 ' ' 1
##
## Residual standard error: 0.08659 on 94 degrees of freedom
## Multiple R-squared:  0.5779, Adjusted R-squared:  0.5734
## F-statistic: 128.7 on 1 and 94 DF,  p-value: < 2.2e-16
##
##
## Call:
## lm(formula = (GR_2$exp - GR_2$theo) ~ GR_2$theo)
##
## Residuals:
```

```
##      Min       1Q   Median       3Q      Max
## -0.20155 -0.03716  0.00006  0.01245  0.20122
##
## Coefficients:
##              Estimate Std. Error t value Pr(>|t|)
## (Intercept)  0.005418   0.014267   0.380    0.705
## GR_2$theo   -0.302880   0.061445  -4.929 3.54e-06 ***
## ---
## Signif. codes:  0 '***' 0.001 '**' 0.01 '*' 0.05 '.' 0.1 ' ' 1
##
## Residual standard error: 0.08659 on 94 degrees of freedom
## Multiple R-squared:  0.2054, Adjusted R-squared:  0.1969
## F-statistic:  24.3 on 1 and 94 DF,  p-value: 3.54e-06
```

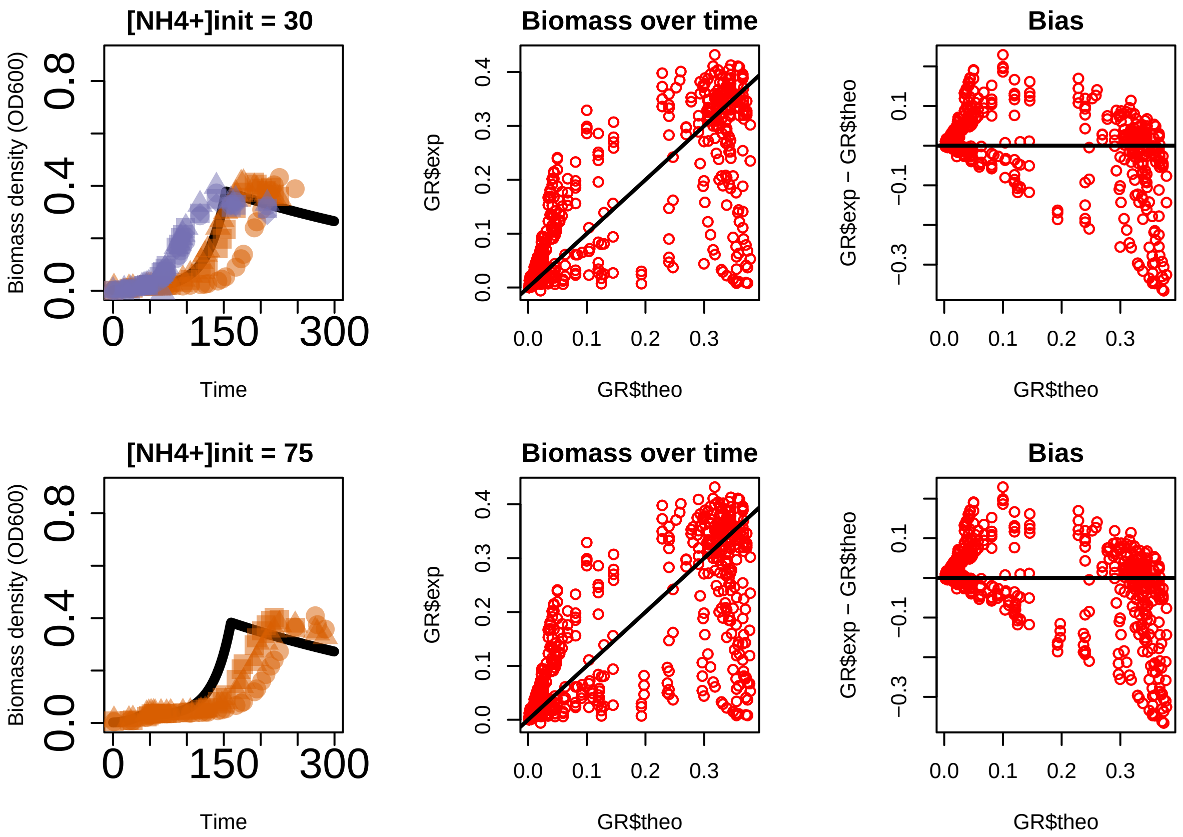


```
## [1] 100
##
## Call:
## lm(formula = (GR_2$exp) ~ GR_2$theo)
##
## Residuals:
##       Min        1Q    Median        3Q       Max
## -0.045292 -0.009992 -0.004104  0.003859  0.134964
##
## Coefficients:
##             Estimate Std. Error t value Pr(>|t|)
```

```
## (Intercept) 0.013979   0.002862   4.884 4.05e-06 ***
## GR_2$theo   0.819461   0.016601  49.362  < 2e-16 ***
## ---
## Signif. codes:  0 '***' 0.001 '**' 0.01 '*' 0.05 '.' 0.1 ' ' 1
##
## Residual standard error: 0.02194 on 98 degrees of freedom
##   (24 observations effacées parce que manquantes)
## Multiple R-squared:  0.9613, Adjusted R-squared:  0.9609
## F-statistic:  2437 on 1 and 98 DF,  p-value: < 2.2e-16
##
##
## Call:
## lm(formula = (GR_2$exp - GR_2$theo) ~ GR_2$theo)
##
## Residuals:
##       Min        1Q    Median        3Q       Max
## -0.045292 -0.009992 -0.004104  0.003859  0.134964
##
## Coefficients:
##              Estimate Std. Error t value Pr(>|t|)
## (Intercept)  0.013979   0.002862   4.884 4.05e-06 ***
## GR_2$theo   -0.180539   0.016601 -10.875  < 2e-16 ***
## ---
## Signif. codes:  0 '***' 0.001 '**' 0.01 '*' 0.05 '.' 0.1 ' ' 1
##
## Residual standard error: 0.02194 on 98 degrees of freedom
##   (24 observations effacées parce que manquantes)
## Multiple R-squared:  0.5469, Adjusted R-squared:  0.5422
## F-statistic: 118.3 on 1 and 98 DF,  p-value: < 2.2e-16
```

```
## [1] 150
##
## Call:
## lm(formula = (GR_2$exp) ~ GR_2$theo)
##
## Residuals:
##       Min        1Q    Median        3Q       Max
## -0.247827 -0.020780  0.004822  0.040170  0.153411
##
## Coefficients:
##             Estimate Std. Error t value Pr(>|t|)
## (Intercept)  0.01543    0.01464   1.054    0.295
## GR_2$theo    0.78745    0.06274  12.551   <2e-16 ***
## ---
## Signif. codes:  0 '***' 0.001 '**' 0.01 '*' 0.05 '.' 0.1 ' ' 1
##
## Residual standard error: 0.08435 on 89 degrees of freedom
## Multiple R-squared:  0.639,  Adjusted R-squared:  0.6349
## F-statistic: 157.5 on 1 and 89 DF,  p-value: < 2.2e-16
##
##
## Call:
## lm(formula = (GR_2$exp - GR_2$theo) ~ GR_2$theo)
##
```

```
## Residuals:
##       Min        1Q    Median        3Q       Max
## -0.247827 -0.020780  0.004822  0.040170  0.153411
##
## Coefficients:
##             Estimate Std. Error t value Pr(>|t|)
## (Intercept)  0.01543    0.01464   1.054  0.29477
## GR_2$theo   -0.21255    0.06274  -3.388  0.00105 **
## ---
## Signif. codes:  0 '***' 0.001 '**' 0.01 '*' 0.05 '.' 0.1 ' ' 1
##
## Residual standard error: 0.08435 on 89 degrees of freedom
## Multiple R-squared:  0.1142, Adjusted R-squared:  0.1043
## F-statistic: 11.48 on 1 and 89 DF,  p-value: 0.001051
```

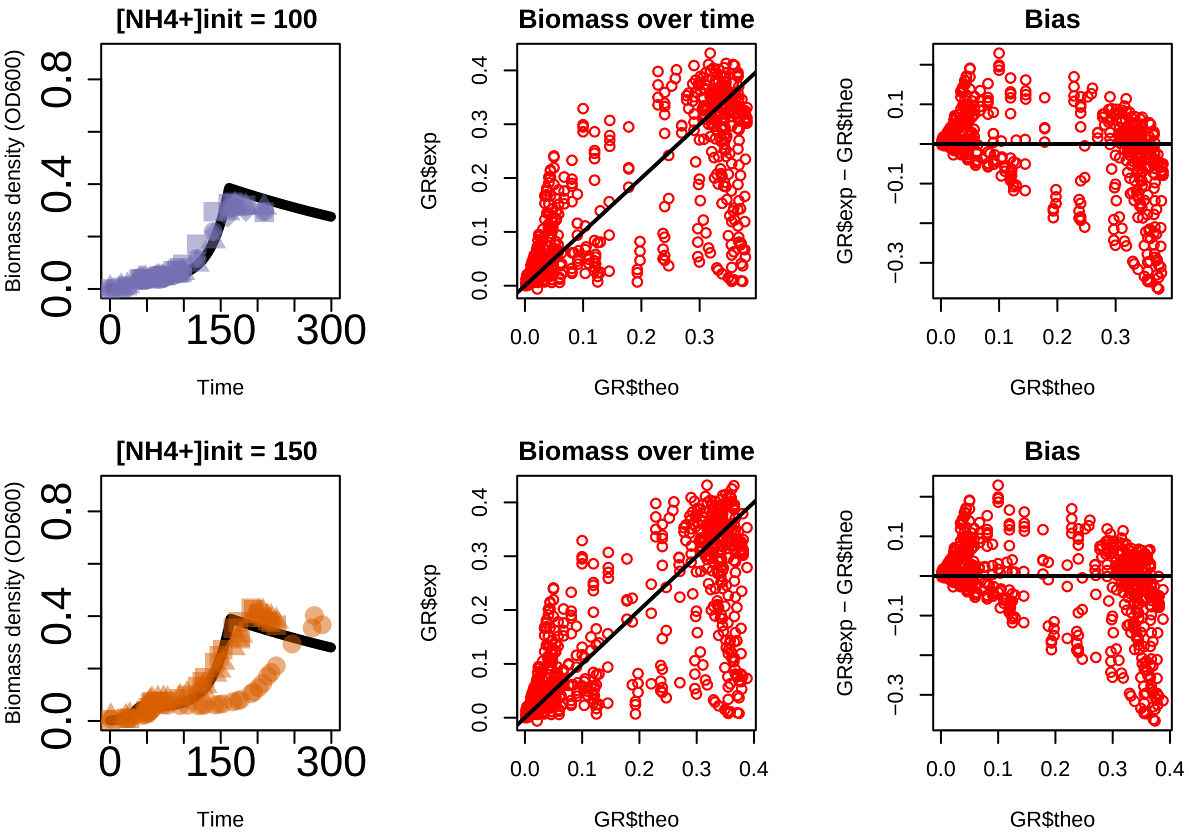


```
## [1] 300
##
## Call:
## lm(formula = (GR_2$exp) ~ GR_2$theo)
##
## Residuals:
##       Min        1Q    Median        3Q       Max
## -0.171375 -0.024504  0.007517  0.022009  0.154042
##
## Coefficients:
```

```
##             Estimate Std. Error t value Pr(>|t|)
## (Intercept)  0.01657    0.00658   2.518   0.0126 *
## GR_2$theo    0.74347    0.02917  25.486   <2e-16 ***
## ---
## Signif. codes:  0 '***' 0.001 '**' 0.01 '*' 0.05 '.' 0.1 ' ' 1
##
## Residual standard error: 0.05291 on 190 degrees of freedom
##   (24 observations effacées parce que manquantes)
## Multiple R-squared:  0.7737, Adjusted R-squared:  0.7725
## F-statistic: 649.5 on 1 and 190 DF,  p-value: < 2.2e-16
##
##
## Call:
## lm(formula = (GR_2$exp - GR_2$theo) ~ GR_2$theo)
##
## Residuals:
##       Min        1Q    Median        3Q       Max
## -0.171375 -0.024504  0.007517  0.022009  0.154042
##
## Coefficients:
##             Estimate Std. Error t value Pr(>|t|)
## (Intercept)  0.01657    0.00658   2.518   0.0126 *
## GR_2$theo   -0.25653    0.02917  -8.794 8.67e-16 ***
## ---
## Signif. codes:  0 '***' 0.001 '**' 0.01 '*' 0.05 '.' 0.1 ' ' 1
##
## Residual standard error: 0.05291 on 190 degrees of freedom
##   (24 observations effacées parce que manquantes)
## Multiple R-squared:  0.2893, Adjusted R-squared:  0.2855
## F-statistic: 77.33 on 1 and 190 DF,  p-value: 8.666e-16
```

```
## [1] 500
##
## Call:
## lm(formula = (GR_2$exp) ~ GR_2$theo)
##
## Residuals:
##      Min       1Q   Median       3Q      Max
## -0.14452 -0.04905  0.01975  0.05097  0.10472
##
## Coefficients:
##             Estimate Std. Error t value Pr(>|t|)
## (Intercept)  0.01414    0.01194   1.184    0.239
## GR_2$theo    0.78078    0.04348  17.957   <2e-16 ***
## ---
## Signif. codes:  0 '***' 0.001 '**' 0.01 '*' 0.05 '.' 0.1 ' ' 1
##
## Residual standard error: 0.06356 on 133 degrees of freedom
##   (1 observation effacée parce que manquante)
## Multiple R-squared:  0.708,  Adjusted R-squared:  0.7058
## F-statistic: 322.5 on 1 and 133 DF,  p-value: < 2.2e-16
##
##
## Call:
```

```
## lm(formula = (GR_2$exp - GR_2$theo) ~ GR_2$theo)
##
## Residuals:
##      Min       1Q   Median       3Q      Max
## -0.14452 -0.04905  0.01975  0.05097  0.10472
##
## Coefficients:
##             Estimate Std. Error t value Pr(>|t|)
## (Intercept)  0.01414    0.01194   1.184    0.239
## GR_2$theo   -0.21922    0.04348  -5.042 1.48e-06 ***
## ---
## Signif. codes:  0 '***' 0.001 '**' 0.01 '*' 0.05 '.' 0.1 ' ' 1
##
## Residual standard error: 0.06356 on 133 degrees of freedom
##   (1 observation effacée parce que manquante)
## Multiple R-squared:  0.1605, Adjusted R-squared:  0.1541
## F-statistic: 25.42 on 1 and 133 DF,  p-value: 1.478e-06
```

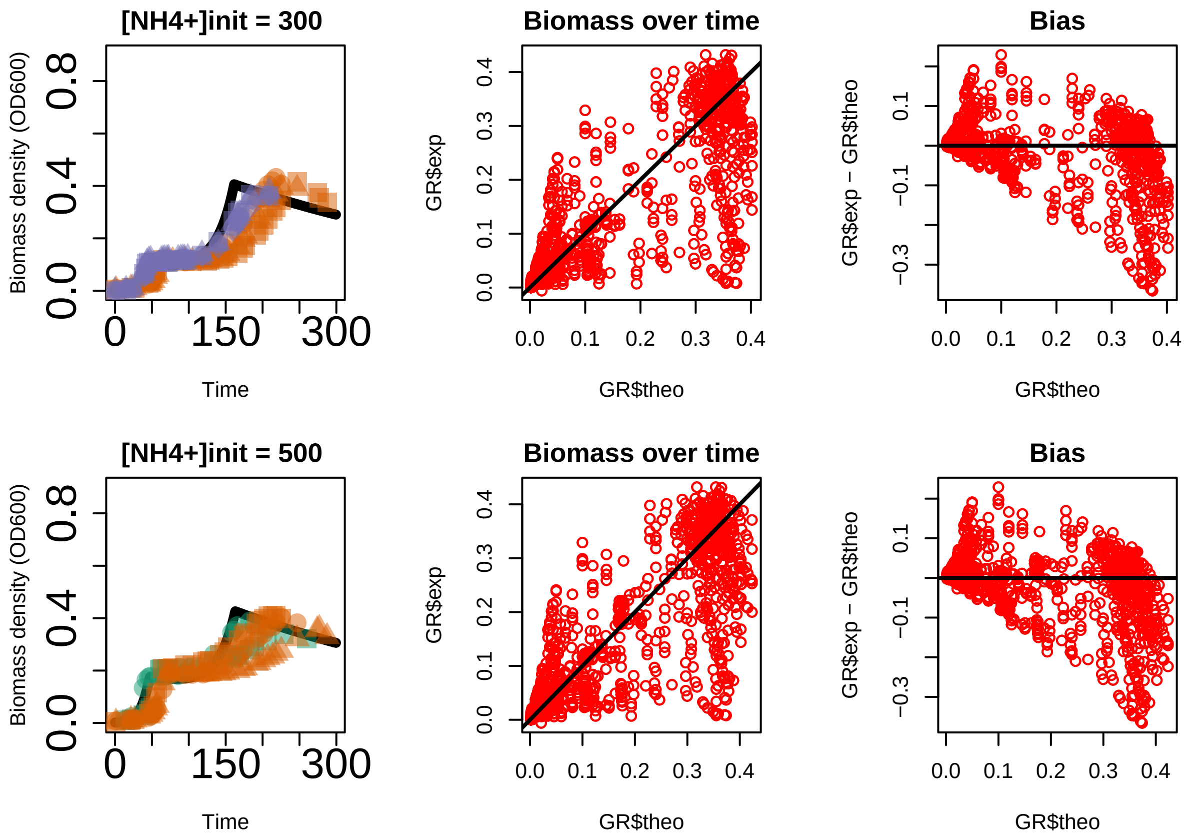


```
## [1] 1000
##
## Call:
## lm(formula = (GR_2$exp) ~ GR_2$theo)
##
## Residuals:
##       Min        1Q    Median        3Q       Max
```

```
## -0.276920 -0.006408  0.023417  0.054913  0.158247
##
## Coefficients:
##               Estimate Std. Error t value Pr(>|t|)
## (Intercept) -0.0001441  0.0144249   -0.01    0.992
## GR_2$theo    0.9824084  0.0514631   19.09   <2e-16 ***
## ---
## Signif. codes:  0 '***' 0.001 '**' 0.01 '*' 0.05 '.' 0.1 ' ' 1
##
## Residual standard error: 0.09009 on 236 degrees of freedom
##   (39 observations effacées parce que manquantes)
## Multiple R-squared:  0.6069, Adjusted R-squared:  0.6053
## F-statistic: 364.4 on 1 and 236 DF,  p-value: < 2.2e-16
##
##
## Call:
## lm(formula = (GR_2$exp - GR_2$theo) ~ GR_2$theo)
##
## Residuals:
##       Min        1Q    Median        3Q       Max
## -0.276920 -0.006408  0.023417  0.054913  0.158247
##
## Coefficients:
##               Estimate Std. Error t value Pr(>|t|)
## (Intercept) -0.0001441  0.0144249  -0.010    0.992
## GR_2$theo   -0.0175916  0.0514631  -0.342    0.733
##
## Residual standard error: 0.09009 on 236 degrees of freedom
##   (39 observations effacées parce que manquantes)
## Multiple R-squared:  0.0004949,  Adjusted R-squared:  -0.00374
## F-statistic: 0.1168 on 1 and 236 DF,  p-value: 0.7328

## [1] 1300
##
## Call:
## lm(formula = (GR_2$exp) ~ GR_2$theo)
##
## Residuals:
##       Min        1Q    Median        3Q       Max
## -0.075949 -0.022473  0.000874  0.020078  0.110051
##
## Coefficients:
##             Estimate Std. Error t value Pr(>|t|)
## (Intercept) 0.005495   0.011896   0.462    0.646
## GR_2$theo   1.059887   0.034947  30.328   <2e-16 ***
## ---
## Signif. codes:  0 '***' 0.001 '**' 0.01 '*' 0.05 '.' 0.1 ' ' 1
##
## Residual standard error: 0.03809 on 43 degrees of freedom
## Multiple R-squared:  0.9553, Adjusted R-squared:  0.9543
## F-statistic: 919.8 on 1 and 43 DF,  p-value: < 2.2e-16
##
##
## Call:
```

```
## lm(formula = (GR_2$exp - GR_2$theo) ~ GR_2$theo)
##
## Residuals:
##       Min        1Q    Median        3Q       Max
## -0.075949 -0.022473  0.000874  0.020078  0.110051
##
## Coefficients:
##             Estimate Std. Error t value Pr(>|t|)
## (Intercept) 0.005495   0.011896   0.462   0.6465
## GR_2$theo   0.059887   0.034947   1.714   0.0938 .
## ---
## Signif. codes:  0 '***' 0.001 '**' 0.01 '*' 0.05 '.' 0.1 ' ' 1
##
## Residual standard error: 0.03809 on 43 degrees of freedom
## Multiple R-squared:  0.06393,    Adjusted R-squared:  0.04216
## F-statistic: 2.937 on 1 and 43 DF,  p-value: 0.09379
```

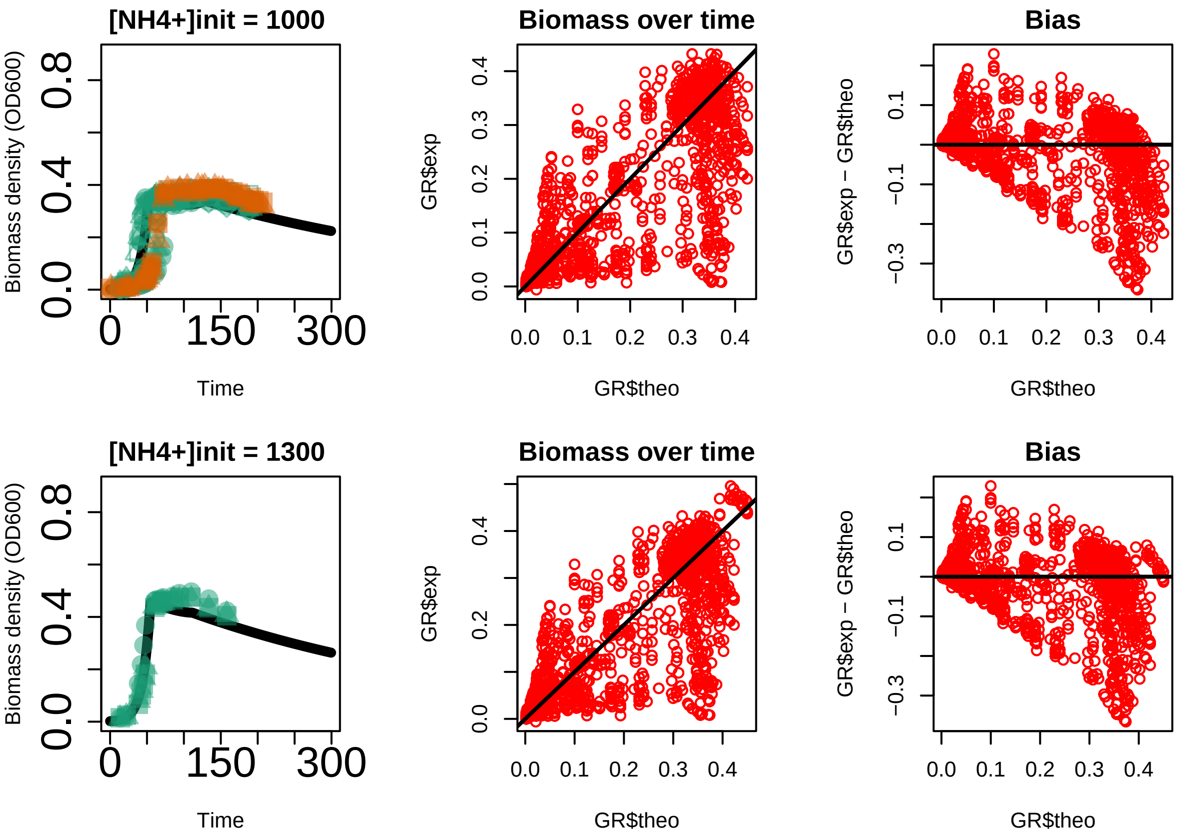


```
## [1] 1500
##
## Call:
## lm(formula = (GR_2$exp) ~ GR_2$theo)
##
## Residuals:
##       Min        1Q    Median        3Q       Max
## -0.051152 -0.024054 -0.001842  0.020364  0.070441
```

```
##
## Coefficients:
##             Estimate Std. Error t value Pr(>|t|)
## (Intercept)  0.05680    0.01818   3.125  0.00746 **
## GR_2$theo    0.92812    0.04682  19.822 1.21e-11 ***
## ---
## Signif. codes:  0 '***' 0.001 '**' 0.01 '*' 0.05 '.' 0.1 ' ' 1
##
## Residual standard error: 0.03488 on 14 degrees of freedom
##   (4 observations effacées parce que manquantes)
## Multiple R-squared:  0.9656, Adjusted R-squared:  0.9631
## F-statistic: 392.9 on 1 and 14 DF,  p-value: 1.214e-11
##
##
## Call:
## lm(formula = (GR_2$exp - GR_2$theo) ~ GR_2$theo)
##
## Residuals:
##       Min        1Q    Median        3Q       Max
## -0.051152 -0.024054 -0.001842  0.020364  0.070441
##
## Coefficients:
##             Estimate Std. Error t value Pr(>|t|)
## (Intercept)  0.05680    0.01818   3.125  0.00746 **
## GR_2$theo   -0.07188    0.04682  -1.535  0.14706
## ---
## Signif. codes:  0 '***' 0.001 '**' 0.01 '*' 0.05 '.' 0.1 ' ' 1
##
## Residual standard error: 0.03488 on 14 degrees of freedom
##   (4 observations effacées parce que manquantes)
## Multiple R-squared:  0.1441, Adjusted R-squared:  0.08292
## F-statistic: 2.356 on 1 and 14 DF,  p-value: 0.1471
```

```
## [1] 1700
##
## Call:
## lm(formula = (GR_2$exp) ~ GR_2$theo)
##
## Residuals:
##      Min       1Q   Median       3Q      Max
## -0.35754 -0.04486  0.02148  0.06096  0.13518
##
## Coefficients:
##             Estimate Std. Error t value Pr(>|t|)
## (Intercept) -0.01190    0.03048   -0.39    0.698
## GR_2$theo    0.96498    0.06980   13.82   <2e-16 ***
## ---
## Signif. codes:  0 '***' 0.001 '**' 0.01 '*' 0.05 '.' 0.1 ' ' 1
##
## Residual standard error: 0.104 on 43 degrees of freedom
## Multiple R-squared:  0.8163, Adjusted R-squared:  0.8121
## F-statistic: 191.1 on 1 and 43 DF,  p-value: < 2.2e-16
##
##
```

```
## Call:
## lm(formula = (GR_2$exp - GR_2$theo) ~ GR_2$theo)
##
## Residuals:
##      Min       1Q   Median       3Q      Max
## -0.35754 -0.04486  0.02148  0.06096  0.13518
##
## Coefficients:
##             Estimate Std. Error t value Pr(>|t|)
## (Intercept) -0.01190    0.03048  -0.390    0.698
## GR_2$theo   -0.03502    0.06980  -0.502    0.618
##
## Residual standard error: 0.104 on 43 degrees of freedom
## Multiple R-squared:  0.005821,   Adjusted R-squared:  -0.0173
## F-statistic: 0.2518 on 1 and 43 DF,  p-value: 0.6184
```

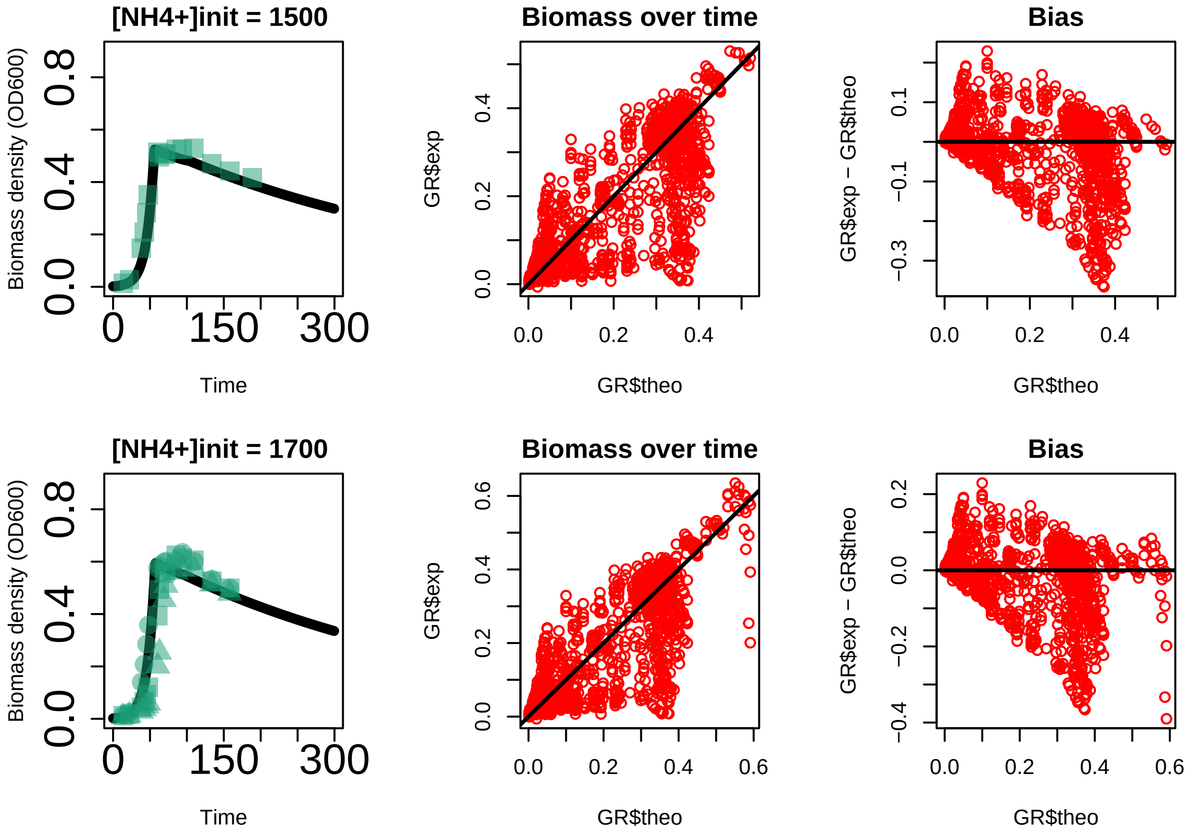


```
## [1] 2000
##
## Call:
## lm(formula = (GR_2$exp) ~ GR_2$theo)
##
## Residuals:
##      Min       1Q   Median       3Q      Max
## -0.42896 -0.11903  0.07497  0.11230  0.22570
##
```

```
## Coefficients:
##             Estimate Std. Error t value Pr(>|t|)
## (Intercept) -0.07247    0.04990  -1.452    0.151
## GR_2$theo    0.95316    0.09646   9.881 3.68e-15 ***
## ---
## Signif. codes:  0 '***' 0.001 '**' 0.01 '*' 0.05 '.' 0.1 ' ' 1
##
## Residual standard error: 0.1733 on 74 degrees of freedom
## Multiple R-squared:  0.5689, Adjusted R-squared:  0.563
## F-statistic: 97.63 on 1 and 74 DF,  p-value: 3.675e-15
##
##
## Call:
## lm(formula = (GR_2$exp - GR_2$theo) ~ GR_2$theo)
##
## Residuals:
##      Min       1Q   Median       3Q      Max
## -0.42896 -0.11903  0.07497  0.11230  0.22570
##
## Coefficients:
##             Estimate Std. Error t value Pr(>|t|)
## (Intercept) -0.07247    0.04990  -1.452    0.151
## GR_2$theo   -0.04684    0.09646  -0.486    0.629
##
## Residual standard error: 0.1733 on 74 degrees of freedom
## Multiple R-squared:  0.003175,   Adjusted R-squared:  -0.0103
## F-statistic: 0.2357 on 1 and 74 DF,  p-value: 0.6287
```

```
## [1] 3000
##
## Call:
## lm(formula = (GR_2$exp) ~ GR_2$theo)
##
## Residuals:
##      Min       1Q   Median       3Q      Max
## -0.51698 -0.09248  0.04803  0.13448  0.22867
##
## Coefficients:
##             Estimate Std. Error t value Pr(>|t|)
## (Intercept) -0.04992    0.02455  -2.034   0.0433 *
## GR_2$theo    0.94663    0.04275  22.145   <2e-16 ***
## ---
## Signif. codes:  0 '***' 0.001 '**' 0.01 '*' 0.05 '.' 0.1 ' ' 1
##
## Residual standard error: 0.173 on 199 degrees of freedom
##   (43 observations effacées parce que manquantes)
## Multiple R-squared:  0.7113, Adjusted R-squared:  0.7099
## F-statistic: 490.4 on 1 and 199 DF,  p-value: < 2.2e-16
##
##
## Call:
## lm(formula = (GR_2$exp - GR_2$theo) ~ GR_2$theo)
##
## Residuals:
```

```
##       Min       1Q   Median       3Q      Max
## -0.51698 -0.09248  0.04803  0.13448  0.22867
##
## Coefficients:
##             Estimate Std. Error t value Pr(>|t|)
## (Intercept) -0.04992    0.02455  -2.034   0.0433 *
## GR_2$theo   -0.05337    0.04275  -1.248   0.2133
## ---
## Signif. codes:  0 '***' 0.001 '**' 0.01 '*' 0.05 '.' 0.1 ' ' 1
##
## Residual standard error: 0.173 on 199 degrees of freedom
##   (43 observations effacées parce que manquantes)
## Multiple R-squared:  0.007771,	Adjusted R-squared:  0.002785
## F-statistic: 1.559 on 1 and 199 DF,  p-value: 0.2133
```

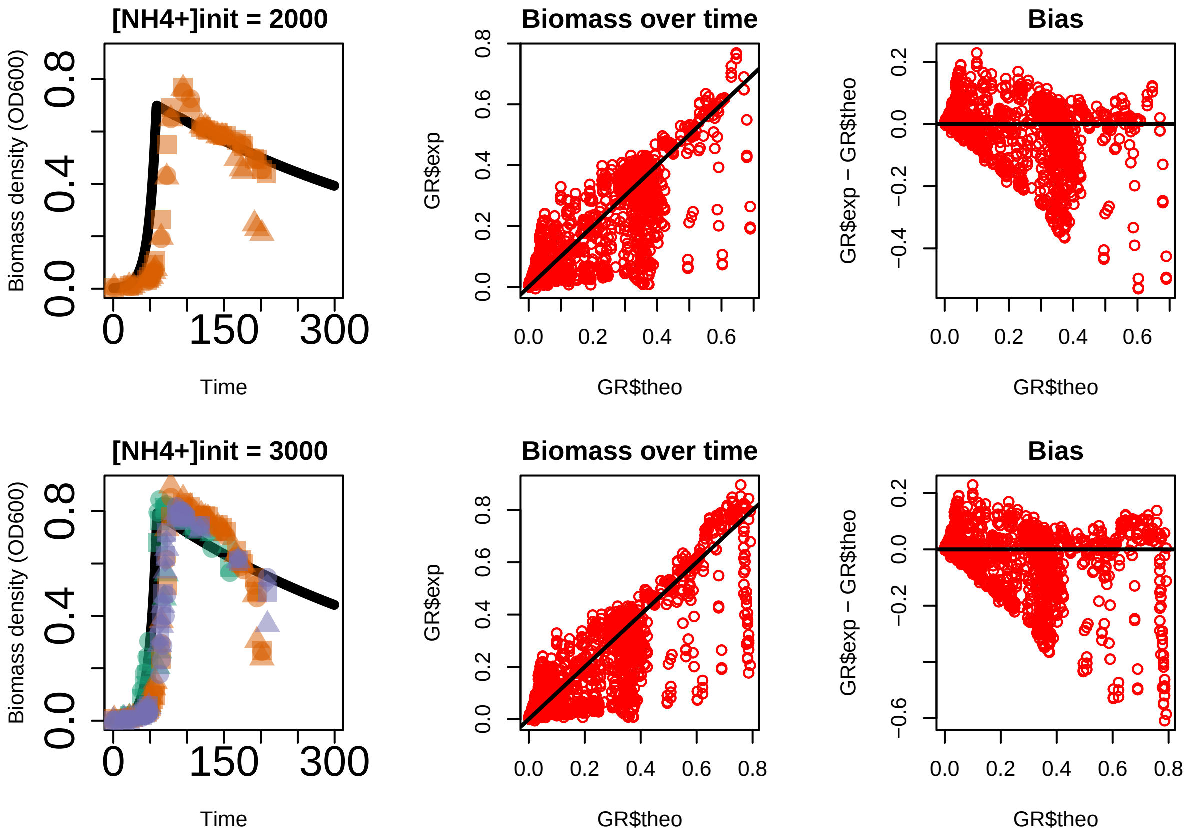


```
#

# dev.off()
```

######END OF DOCUMENT#####
