# Supplementary material for: A diazotrophy-ammoniotrophy dual growth model for the sulfate reducing bacterium Desulfovibrio vulgaris var. Hildenborough
Source: Comput Struct Biotechnol J. 2023 May 7;21:3136–48. doi: 10.1016/j.csbj.2023.05.007 (PMC10244686; doi:10.1016/j.csbj.2023.05.007)
Supplement: Supplementary file 3 — SI Supplementary Material [file mmc3.pdf]

Supplementary Information to "A Diazotrophy-Ammoniotrophy Dual Growth Model for the Sulfate Reducing Bacteria Desulfovibrio vulgaris var. Hildenborough"

Authors: Romain Darnajoux[1,2], Keisuke Inomura[3], and Xinning Zhang[1,2]

Affiliations: [1]Department of Geosciences, Princeton University, Princeton, NJ 08544

[2]High Meadow Environmental Institute, Princeton University, Princeton, NJ 08544

[3]Graduate School of Oceanography, University of Rhode Island, Kingston, RI 02881

*Corresponding author: Romain Darnajoux (romain.darnajoux@hotmail.fr, +1 609 379 9628)

ORCID: Romain Darnajoux (0000-0002-4996-0067), Keisuke Inomura (0000-0001-9232-7032), Xinning Zhang (0000-0003-2763-1526)

Key words: *Desulfovibrio vulgaris* var Hildenborough, Diazotrophy, Biological nitrogen fixation, Benthic sediments.

FIGURE S1

A

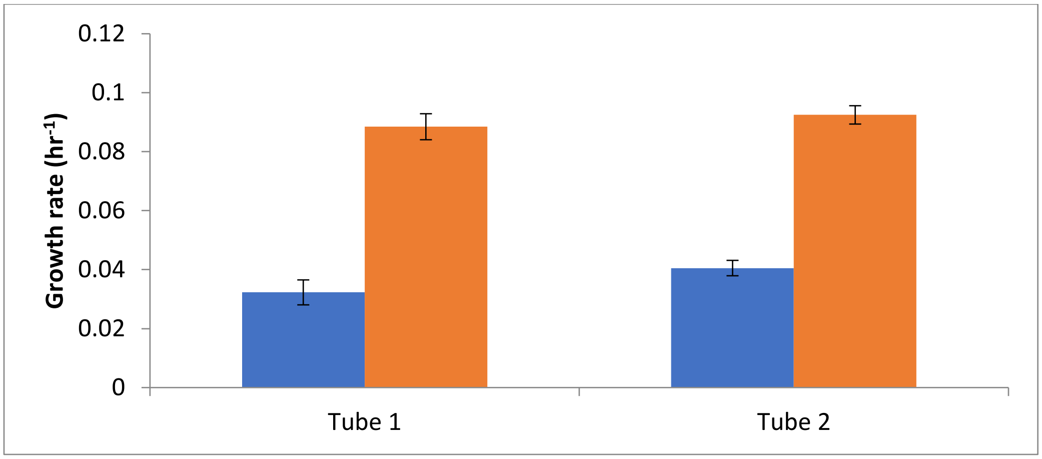

B

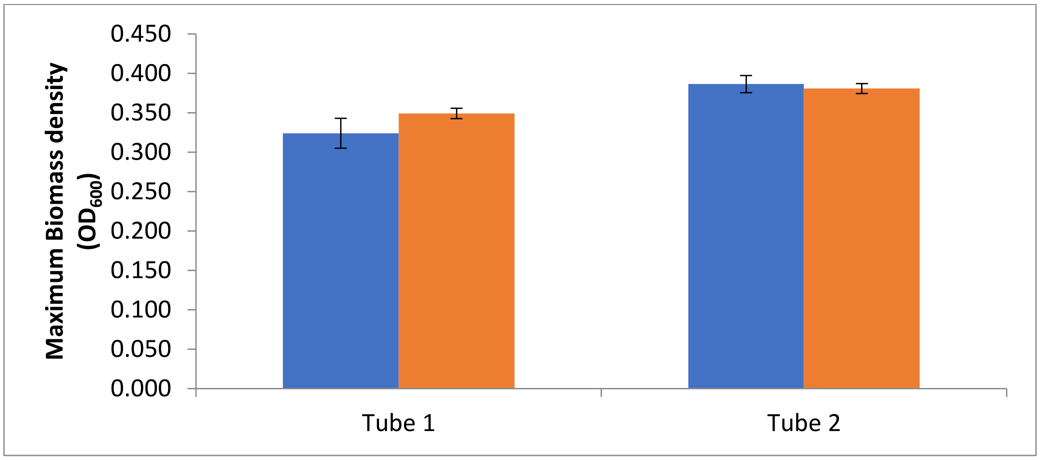

Figure S1. Effect of septa aging on growth of *Desulfovibrio vulgaris*, showing the comparison of growth rate (A) and maximum biomass density (B) between replicated culture with two different batches of septa, one of them (Tube 1) showing clear sign of wear. Culture was prepared with (orange) and without (blue) initial addition of NH_4^+ to reach 500µM.

Interpretation: The results clearly indicate septa quality (Tube 1 vs Tube 2) impact N_2-trophic growth (Blue bars) more than NH_4-trophic growth (orange bars). This is certainly due to small quantity of O_2 leaking into the culture and causing damaged to nitrogenase enzymes or additional cost for protection.

FIGURE S2

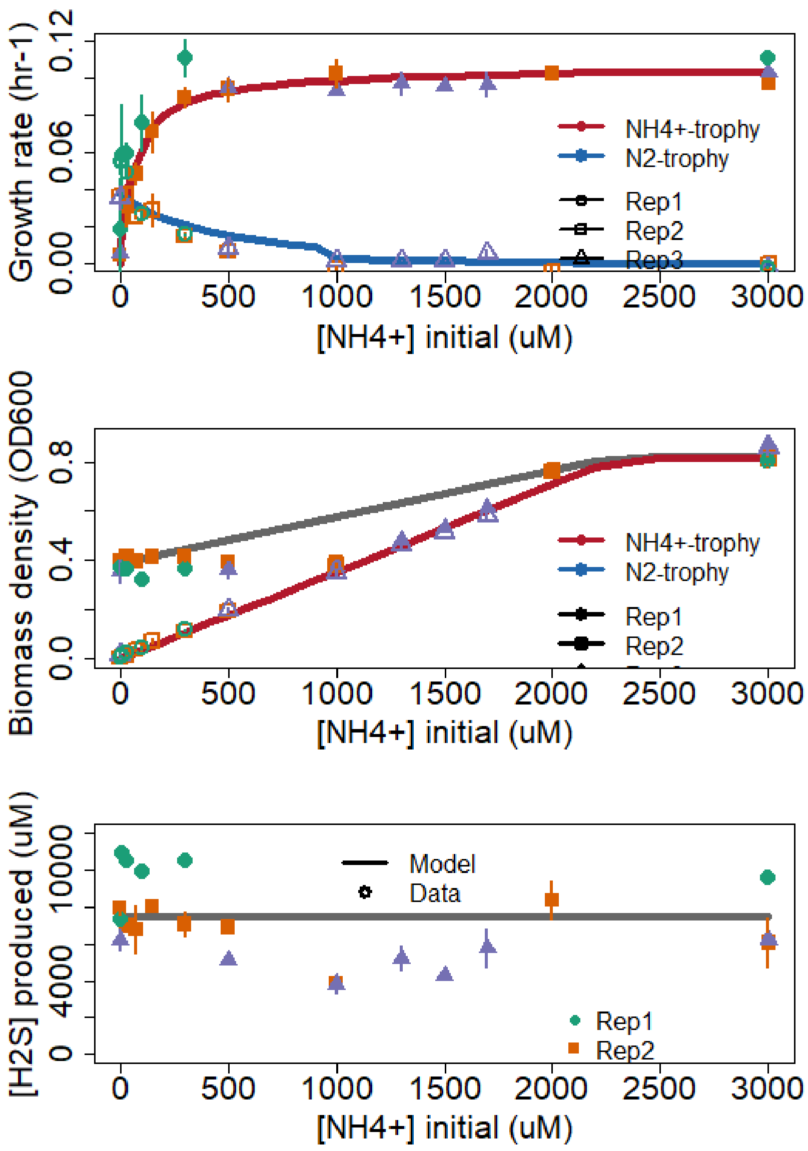

Figure S2. Comparison of model output with observed experimental data when no dead rate and no limiting step was implemented. Experimental data were acquired from three independent replicate conducted under similar condition with increasing $[NH_4^+]_{init}$. Panel (A) show evolution of growth rate for diazotrophic and ammoniotrophic phases, Panel (B) shows the maximum biomass obtained at the first growth plateau (i.e., under ammoniotrophic condition) and at the end of growth, and panel (C) shows maximum H_2S concentration produced during growth, a proxy of sulfate usage. Error bars are SD.

FIGURE S3

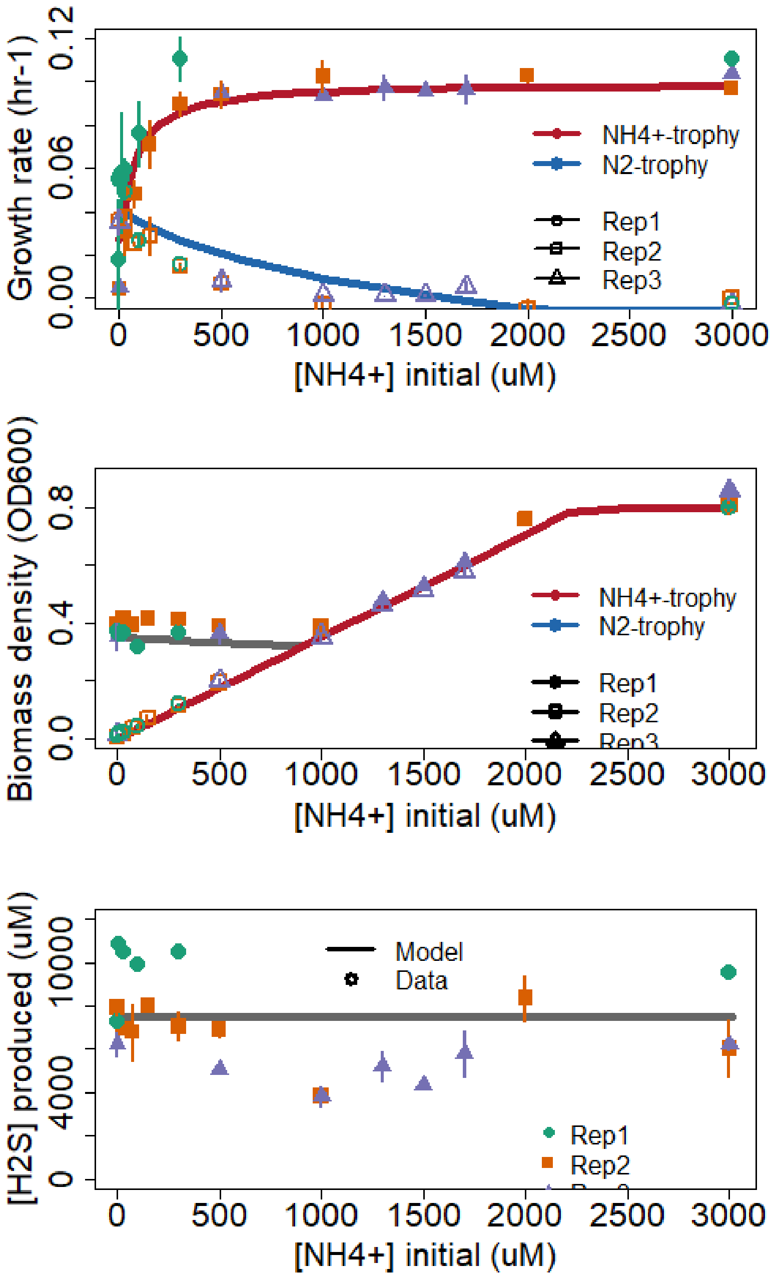

Figure S3. Comparison of model output with observed experimental data when no limiting step was implemented, showing a clear mismatch between [H_2S] production between model and experiment. Experimental data are identical to Main text Figure 3 and represent three independent replicates conducted under similar condition with increasing [NH_4^+]$_{init}$. Error bars are SD.

Interpretation: Figure S2 and S3 demonstrate that an arbitrary limiting step that prevents N_2-trophy over cell density of OD_{600}= 0.4 is require to reproduce all the feature of the data.

FIGURE S4

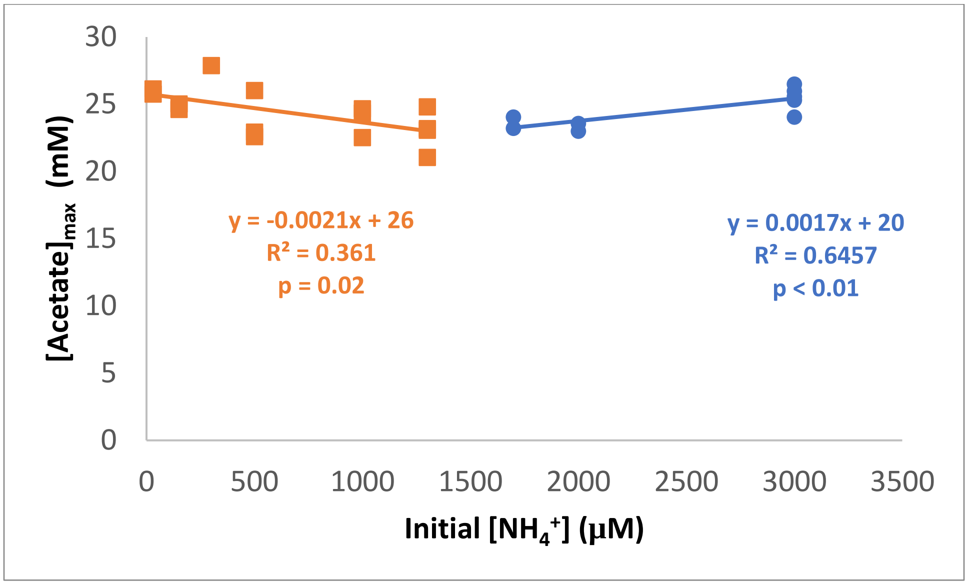

Figure S4. Evolution of acetate maximal concentration produced at the end of growth, showing a statistically significant decrease of $[Acetate]_{max}$ between 0 and 1500 µM and a statistically significant increase of $[Acetate]_{max}$ between 1500 and 3000 µM. Data originate from two replicate experiments. Acetate concentration was evaluated using HPLC-DAD following protocol from Zeppa et al 2001. Supernatant were kept for three years in different conditions (Close Butch Tube at 4°C, centrifuged supernatant at 20°C) before analysis, and actual value could represent an underestimation of

Giuseppe Zeppa, Lorenza Conterno, and Vincenzo Gerbi, 2001, "Determination of Organic Acids, Sugars, Diacetyl, and Acetoin in Cheese by High-Performance Liquid Chromatography", Journal of Agricultural and Food Chemistry 49 (6), 2722-2726, DOI: 10.1021/jf0009403

FIGURE S5

A

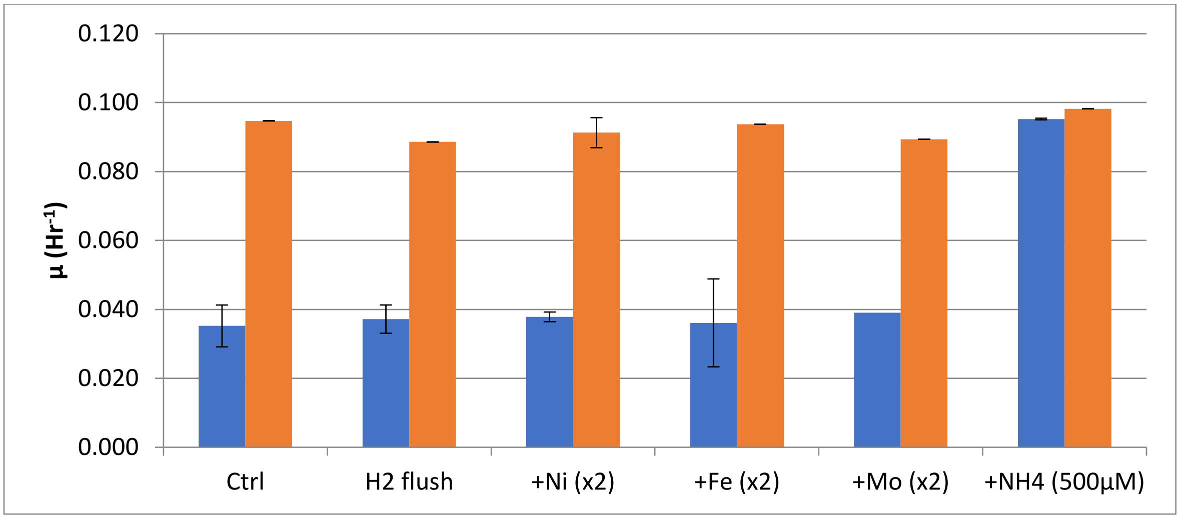

B

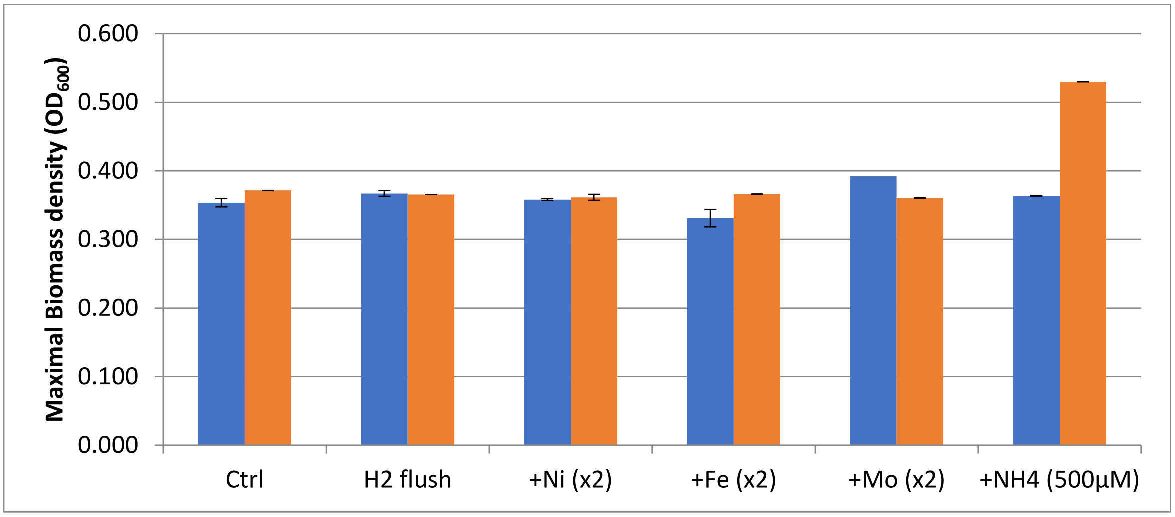

Figure S5. Effect of various component on the growth of Desulfovibrio vulgaris, showing no difference in growth rate (A) and maximum biomass (B) vs. control diazotrophic conditions ("Ctrl") when double concentration of nickel ("+Ni (x2)"), iron ("+Fe (x2)"), molybdenum ("+Mo (x2)") was used, or when initial 2% H_2 headspace was replace with 100% N_2 (« H2 flush"). Experiments were conducted using 30 mM pyruvate and 10 mM SO_4^{2-}, with (blue) and without (orange) presence of 500 µM ammonium in the media at the start of the experiment. Data for ammonium addition in the initial media ($[NH_4^+]$ = 500 µM, "+NH4 (500uM)") are shown for comparison.

FIGURE S6

A

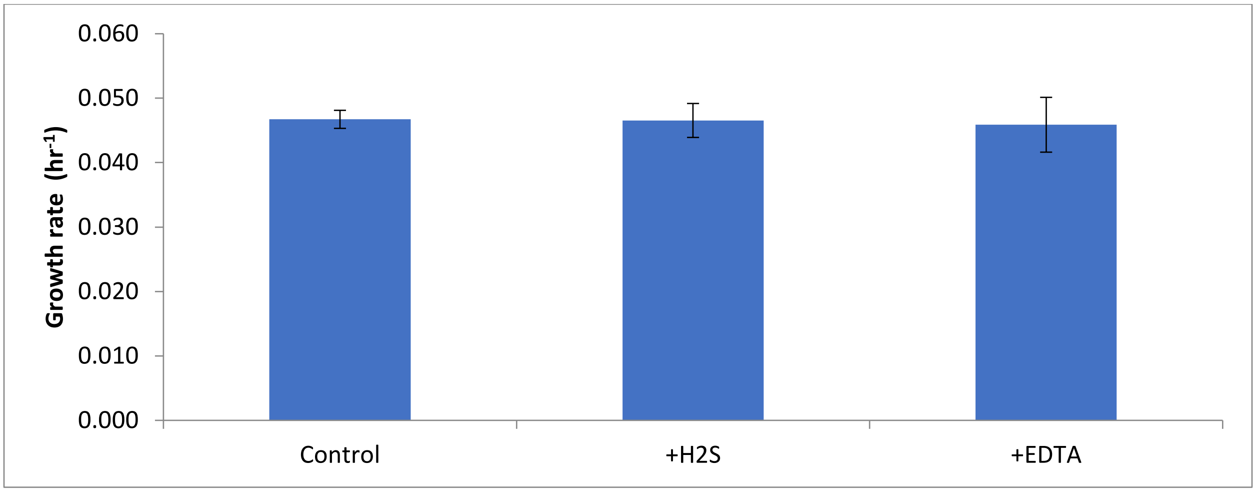

B

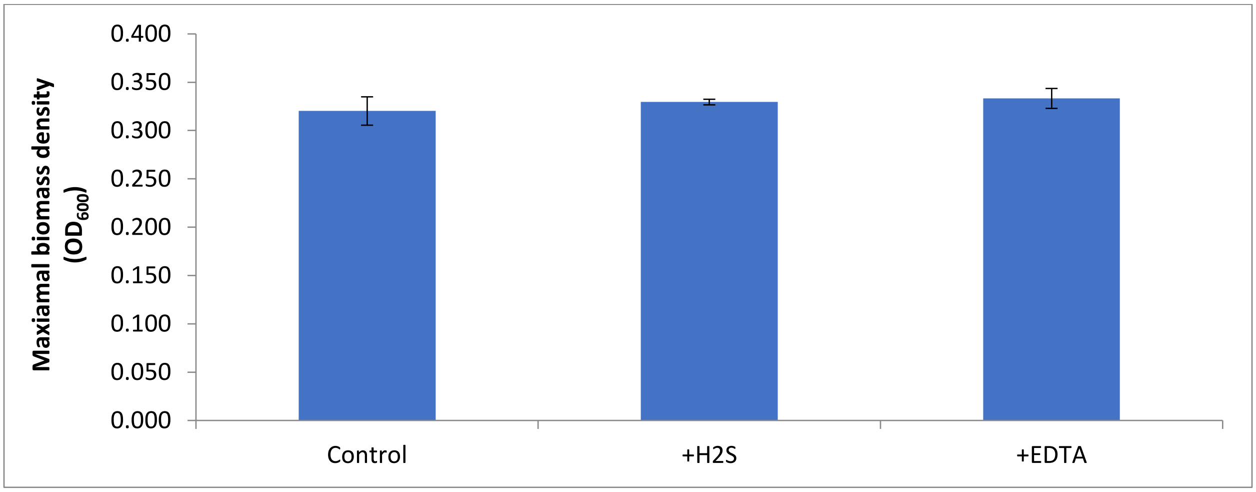

Figure S6. Effect of H_2S (4 mM), EDTA (0.05 mM) on the growth of *Desulfovibrio vulgaris*, showing no difference in growth rate (A) and maximum biomass density (B) vs. control diazotrophic conditions (Control) realized in the same condition. Experiments were conducted in four replicates using 30mM Pyruvate and 10mM SO_4^{2-}. Error bars are SD.

Interpretation: Addition at the onset of growth of H_2S at similar concentration than what is found at the end of batch culture growth when biomass yield appears to be limited the most ($[NH_4^+]_{init}$ = 1000 µM, $[H_2S]$ = 4mM) do not affect growth rate and maximal biomass density yield, indicating millimolar $[H_2S]$ do not affect N_2-trophy. Similarly, presence of a strong iron chelating agent (EDTA at 0.05 mM) does not influence the results.

Method S1

S1. Calculation of initial pyruvate concentration from sulfate usage data

$$[Pyr]_{init} = r_{SO4} \times Y_{\max} \times 4 \,/\, V_{culture}$$

With $[Pyr]_{init}$ is initial pyruvate concentration, r_{SO4} ($\mu mol_{SO4}.ODmL^{-1}$) is the specific sulfate usage (see Figure 2A and Table 2), Y_{max} is the maximum biomass yield (ODmL, Table 2), 4 is the theoretical stoichiometry between pyruvate and sulfate, and $V_{culture}$ is the volume of the media (10mL)
